# Fracture characteristics and outcomes in osteogenesis imperfecta: A systematic review

**DOI:** 10.1016/j.bonr.2026.101958

**Published:** 2026-10-10

**Authors:** Lothar Seefried, Rosa Bou, Judith Bubbear, Ruggero Lanzafame, Thomas Zoller, Samantha Prince, Maria Rapoport, Ogün Sazova, Clive Whitcher, Franco Antoniazzi

**Affiliations:** a University of Würzburg, Würzburg, Germany; b Hospital Sant Joan de Déu, Barcelona, Spain; c Royal National Orthopaedic Hospital, London, UK; d Università di Verona, Verona, Italy; e Mereo BioPharma Group plc, London, UK; f Wickenstones Ltd, Oxfordshire, Oxford, UK

**Keywords:** Osteogenesis imperfecta, Fractures, Healing time, Signs and symptoms

## Abstract

**Objectives:**

Osteogenesis imperfecta (OI) is a rare genetic disorder marked by bone fragility and increased fracture risk, adversely affecting quality of life (QoL). The overall fracture burden remains incompletely defined. This systematic review synthesised evidence on fracture characteristics (onset, frequency, anatomical location, risk factors) and outcomes (healing time and downstream signs, symptoms, and events [SSEs]).

**Methods:**

We sought English-language cohort studies, patient surveys, and trials enrolling ≥10 individuals with OI, reporting fracture characteristics and fracture outcomes, published in or after 2000. We searched MEDLINE, Embase, Cochrane CENTRAL, PsycINFO, ClinicalTrials.gov, ICTRP, and CPCIS-S through March 15, 2024 and relevant congresses through December 31, 2024. Outcomes were qualitatively synthesised.

**Results:**

Most included studies (*n* = 87) were retrospective. First fractures typically occurred in early childhood, with earlier onset in those with Type III versus Type I. Annual fracture rates ranged from 0 to >10, varying by OI type; one to several fractures per year were typical. Fractures affected long bones, vertebrae, and other bones without a predominant location, likely reflecting heterogeneous reporting. Fracture rates were slightly higher in children than adults, and highest in those with Type III across ages. Few healing-time data were available. Downstream SSEs included pain, impaired functioning and QoL, and risk of same-site refracture.

**Discussion:**

OI is associated with an early-onset, lifelong fracture burden, with considerable individual variability. Interpretation is limited by heterogeneous study designs, inconsistent fracture recording, and limited prospective data. Findings highlight a heavy fracture burden and a considerable unmet need for effective interventions to prevent OI-related fractures.

Registration: PROSPERO (CRD42024536760).

## Highlights

• OI causes an early-onset, lifelong fracture burden.
• First fractures occur in childhood, at a younger age in those with Type III vs Type I.
• Fracture rates vary widely; one to several fractures annually are typical.
• Fractures affect multiple sites, without a consistently predominant location.
• Data are limited, showing a high burden and unmet need for fracture prevention.

## 1 Introduction

### 1.1 Background

Osteogenesis imperfecta (OI) is a rare genetic condition characterised by low bone mass and bone fragility, leading to an increased susceptibility to fractures (Deguchi et al., 2021; Marini and Dang Do, 2020). Between 80 and 90% of cases in Western countries have been estimated to be caused by pathogenic variants in the type I collagen genes *COL1A1* and *COL1A2* and inherited in an autosomal dominant fashion (Marini et al., 2017; Jovanovic and Marini, 2024). Also known as brittle bone disease, OI is a rare condition, with an estimated incidence of 1 in 15,000–20,000 births (Subramanian and Viswanathan, 2019).

The condition is diverse. It can be classified by genetic background and by specific clinical phenotypes, which vary in severity (Deguchi et al., 2021; Rauch and Glorieux, 2004). OI caused by variants in *COL1A1* or *COL1A2* are classified Type I–IV by phenotype. Type I OI is the mildest and typically presents with blue sclerae but without bone deformities. By contrast, Type II is the most severe, typically resulting in perinatal lethality. Type III is the most severe to affect people who survive the neonatal period; these individuals develop severe progressive skeletal deformities and achieve very short stature. People with Type IV OI experience mild to moderate bone deformities and short stature; they have normal sclerae.

Further OI types are caused by a wide range of distinct gene variants other than *COL1A1* and *COL1A2*, resulting in multiple pathways toward bone fragility. For example, OI Types V and VI arise from, respectively, dominant and recessive genetic alterations affecting bone mineralization, while Types VII, VIII, and IX arise from recessive variants on three different genes leading to absent procollagen prolyl 3-hydroxylation complex components. OI types X, XI and XIII occur with variants in genes for collagen processing and cross-linking. Other types are caused by recessive variants leading to faulty osteoblast differentiation (Jovanovic and Marini, 2024).

### 1.2 Current management

The goals of OI management are to reduce fractures, treat fracture complications and non-skeletal symptoms, and improve mobility, function and quality of life (Hill et al., 2019). A multidisciplinary approach to management is strongly recommended. Treatment regimens include pharmacological interventions, surgeries, and physical rehabilitation, often alongside tailored vitamin D and calcium supplementation (Marini et al., 2017).

To reduce fracture risk in patients with OI, medications can be prescribed. However, they are predominantly used off-label and lack conclusive evidence to support their use or to describe their risk-benefit profiles in people with OI (Liu et al., 2023; Westerheim et al., 2024a; Polyzos et al., 2019; Dwan et al., 2016). Such pharmaceutical interventions include intravenous bisphosphonates such as pamidronate, zoledronate or neridronate, or oral bisphosphonates like alendronate and risedronate; further, hormone therapies such as the recombinant parathyroid hormone analogue teriparatide, and antibodies such as the anti-receptor activator of nuclear factor-kappaB ligand (RANKL) antibody denosumab and the anti-sclerostin antibody romosozumab, have been used in clinical studies and specific clinical settings (Botor et al., 2021). Analgesics are also used for pain management (Marr et al., 2017). Bisphosphonates, which inhibit bone resorption and increase bone mass, are most commonly used despite not being specifically approved for OI in most countries. They are the only class of drug routinely used to specifically treat children with OI. Only the bisphosphonates pamidronate and neridronate have been approved for OI in Japan (Harada et al., 2020) and in Italy (Gatti et al., 2013), respectively. In paediatric clinical trials, bisphosphonates have demonstrated inconsistent fracture responses; they have not been proven effective for reducing fractures in adults (Dwan et al., 2016; Hald et al., 2015). This lack of evidence-based pharmaceutical interventions to address fracture risk represents a substantial unmet need in OI.

### 1.3 Unmet need

OI profoundly affects individuals across physical, social, and emotional domains (Hill et al., 2014), and it imposes considerable burdens on these individuals and their caregivers (Abraham et al., 2025; Aratti and Zampini, 2024). However, this burden is uneven, as OI is a heterogenous disorder and many genetic and environmental factors can influence fracture risk. While some individuals show milder signs and symptoms and fewer clinical features, others experience frequent fractures, severe bone deformities, significant physical disabilities, and a shortened lifespan (Marini et al., 2017; Nijhuis et al., 2022).

Multiple studies have explored the effects of OI on the lives of people who live with it (Westerheim et al., 2024a; Rapoport et al., 2023). The IMPACT Survey showed that OI is not only associated with substantial financial costs, but also strongly affects physical, socioeconomic, and mental well-being, with more severe OI and increased fracture frequency driving greater quality of life (QoL) impact (Westerheim et al., 2024b; van Welzenis et al., 2024; Hart et al., 2024).

This burden demonstrates the need for an effective and targeted treatment for OI to reduce fracture rates.

## 2 Objectives

Despite the centrality of fracture burden to the QoL of people with OI, this burden is inconsistently documented due to the heterogeneity and rarity of the condition. Moreover, fracture frequency varies by OI type, severity, and age (Folkestad et al., 2017; Joshi et al., 2023). Data on fracture recovery are also scarce (Munk et al., 2022).

Therefore, this study aimed to review published evidence on fracture burden in people with OI. Its objectives were to assess the following:

• **Fracture characteristics,** including age at first fracture; fracture frequency measured annually and/or by lifetime; fracture location; and risk factors.
• **Fracture outcomes,** including fracture healing time and the frequency of downstream signs, symptoms, and events (SSEs) associated with fractures.

## 3 Methods

The systematic review sought to identify published studies relating to the clinical burden of fractures in people with OI. It was performed in line with Centre for Reviews and Dissemination (CRD) guidelines on conducting such reviews (CRD, 2013). The review protocol was registered with PROSPERO (CRD42024536760). We report it following 2020 Preferred Reporting Items for Systematic Reviews and Meta-Analyses (PRISMA) (Page et al., 2021).

### 3.1 Search and study selection

We first conducted a comprehensive, systematic search of the literature to find all potentially relevant studies, then screened them according to inclusion and exclusion criteria before extracting relevant data. Information sources included the following electronic bibliographic databases and congress proceedings:

o Electronic bibliographic databases: MEDLINE (via PubMed), Embase (via Embase.com), Cochrane CENTRAL, PsycINFO (via Dialog), ClinicalTrials.gov, ICTRP (via WHO), and CPCIS-S (via Clarivate).
o Additional sources from recent congresses: the QUALITYOFLIFE4All Conference (2023 and 2024), the International Conference on Osteogenesis Imperfecta (ICOI; 2022), the International Conference on Children's Bone Health (ICCBH; 2022), the European Calcified Tissue Society Congress (ECTS; 2021–2023), the World Congress on Osteoporosis, Osteoarthritis and Musculoskeletal Diseases (WCO-IOF-ESCEO; 2021–2024), and the annual meeting of the American Society for Bone and Mineral Research (ASBMR).

Searches of electronic bibliographic databases were from January 1, 2000, to March 15, 2024. Searches of congress proceedings were from January 1, 2021, to December 31, 2024.

Full search terms and strategies are provided in **Supplementary Table 1** to **Supplementary Table 8**. Bibliographies of all included studies were searched for relevant studies after the full text screening stage using citationchaser, an R package and Shiny application to conduct snowballing from a starting set of reports (Haddaway et al., 2022). Reports identified by citationchaser were then deduplicated using Endnote X8 against itself and records in the original database.

### 3.2 Study selection criteria

Inclusion and exclusion criteria appear in the PICOS (Population, Intervention, Comparator, Outcomes, Study design) table (Table 1). Briefly, the population of interest was adults and children with OI. Studies that reported fracture characteristics (fracture rate/frequency, age at first fracture; fracture type; fracture location) and fracture outcomes (time to radiographic fracture recovery; SSEs stratified by fracture history or using fracture history as an analytic factor) were included. Those reporting categorically/non-numerically reported fractures or fracture count data without a time horizon were excluded. We did not include a prenatal search term. We also excluded studies that evaluated mixed populations (i.e. those with and without OI) without providing subgroups and those that included fewer than 10 participants.

**Table 1:** PICOS criteria for inclusion and exclusion of studies.

Characteristics	Inclusion criteria	Exclusion criteria
Population	• People with OI (adults and children) • ≥10 participants included	• People without OI • Mixed populations (population including individuals without OI)^a^ • <10 study participants
Interventions	None or any	N/A
Comparators	None or any	N/A
Outcomes	Fracture characteristics: • Fracture rate/frequency • Age of first fracture • Numerically reported fracture type and location Fracture outcomes: • Time to radiographic fracture recovery b • Numerically reported SSEs stratified by fracture history or using fracture history as an analytic factor	• Categorically/non-numerically reported fractures • Fracture count data without a time horizon
Study design	• Cohort studies, including patient registry data • Patient surveys • Randomised controlled trials^c^	• Case reports • Reviews (including systematic reviews) • Cross-sectional studies other than surveys
Language	English	Non-English
Publication type and status	Publication type: • Full-text manuscripts • Conference abstracts and posters^d^ Publication status: • Published • *E*-publications ahead of print • In-process	Publication type: • Letters • Editorials • Notes • Lectures
Data of publication	2000 onwards	Pre-2000

Notes:

Abbreviations: OI, osteogenesis imperfecta; SSE, signs, symptoms, and events.

a Studies with OI-specific subgroup data were included.

b Radiographic healing time may be defined as bridging of fracture site by callus, obliteration of the fracture line and continuity (Bigham-Sadegh and Oryan, 2015).

c Data from randomised controlled trials were recorded to anchor real-world fracture rates and outcomes.

d Only conferences from the past 3 years were searched.

Study designs included cohort studies, patient surveys, and randomised controlled trials (RCTs). We included conference abstracts and posters in addition to full manuscripts. Case reports, systematic reviews, and cross-sectional studies were excluded, as were studies published prior to 2000. Only studies published in English or with at least an English-language abstract were included.

### 3.3 Screening of studies

Duplicate publications, including interim reports, were treated as one study. Deduplication took place before title and abstract screening, during title, abstract, and full text screening, and during data extraction using EndNote X8. Two independent researchers screened titles and abstracts, and two screened full texts. Any disagreements about study eligibility were resolved by a third researcher.

### 3.4 Data extraction and synthesis

Data extraction was conducted according to a list of pre-specified variables (**Supplementary Table 9**). It took place in two phases. First, we extracted background information from all relevant reports. Second, we extracted more details from studies that reported numeric fracture rates (as opposed to reports that provided data from which fracture rates could potentially be calculated) or other outcomes of interest. We prioritised studies that reported rates, as such studies provided more robust evidence than reports from which rates needed to be calculated and assumptions made. For both data extraction phases, one researcher extracted the data and a second checked the data extraction forms. Disagreements were resolved according to a pre-defined strategy using consensus and arbitration.

Extracted data were summarised qualitatively according to the following outcomes: age of first fracture, fracture rates, healing time, and SSEs. Fracture rates were further synthesised by the sub-categories of OI type, fracture location, and age group. Our objective to capture associations between risk factors and fracture frequency was met, if incompletely, by stratifying fracture rates by age and OI type.

All data were extracted as reported. No additional consultation with study authors was conducted, no automated tools were used, and no assumptions were made regarding any missing or unclear information.

We collated and summarised data in a qualitative synthesis and did not perform quantitative analysis. Data were set by our research questions (age of first fracture, fracture rates, healing time and downstream events and SSEs) and synthesised by appropriate units. Fracture rates reported per year were derived separately from rates reported per person-year. Where possible, values provided per numbers of person-years were adjusted to a common denominator of 100,000 person-years for ease of comparison. Mean and median values and estimates of variance (e.g. standard deviations [SD] or range) were presented as reported. Data relating to children and adults were synthesised separately to examine risks due to age-based differences, using the definitions provided by each study.

### 3.5 Risk of bias assessment

Following data extraction, one researcher conducted risk of bias assessments using the Institute of Health Economics (IHE) Quality Appraisal Checklist for Case Series Studies (Institute of Health Economics, 2014) to evaluate cohort studies and the Critical Appraisal Programme (CASP) RCT Checklist (Critical Appraisal Programme, 2023) to evaluate RCTs. The IHE checklist evaluates studies based on objective, design, population, intervention and co-intervention, outcome measures, statistical analysis, results and conclusion, and competing interests and sources of support. The CASP checklist evaluates study design, soundness of methodology, and application of study results. We adapted the checklists to align with our research questions and the types of studies we included. Certain items were not assessed, as they were considered irrelevant to the current review. For cohort studies, we did not assess intervention methods, co-intervention methods, or statistical analyses. For RCTs, we did not assess local applicability of the results.

Questions in both assessments were answered for each included study: ‘yes’, ‘no’, ‘unclear’, or ‘not applicable’. Scores were assigned to each study to assess quality. Quality assessment results were calculated as percentages of positive responses out of total questions (excluding ‘not applicable’) where positive responses were given for ‘yes’ and negative scores were given for ‘no’ or ‘unclear’. A study was considered high quality if it had a score of >66.66%, medium quality with a score of 33.33–66.66%, and low quality with a score of <33.33%.

## 4 Results

A total of 4859 publications were identified through database searches. An additional 678 records were identified through searches of congresses and backward citation chasing via reference lists. After removal of duplicates (*n* = 1583) and clearly irrelevant records (*n* = 261), 3693 publications were screened by title and abstract. Of the records sought for full report retrieval (*n* = 444), 438 reports were assessed for eligibility. Six records could not be retrieved.

Reasons for excluding 296 reports at full text screening were irrelevant publication type (*n* = 18); duplication (*n* = 1); no outcomes of interest (*n* = 244); not in English (*n* = 7); irrelevant population (*n* = 10); or not meeting minimum sample size (*n* = 16) (**Supplementary Table 10**).

A total of 142 reports met the eligibility criteria for first-phase data extraction, of which 87 studies were included for second-phase data extraction with results reported here (Fig. 1). Characteristics of the 87 included studies are shown in **Supplementary Table 11.**

**Fig. 1:**
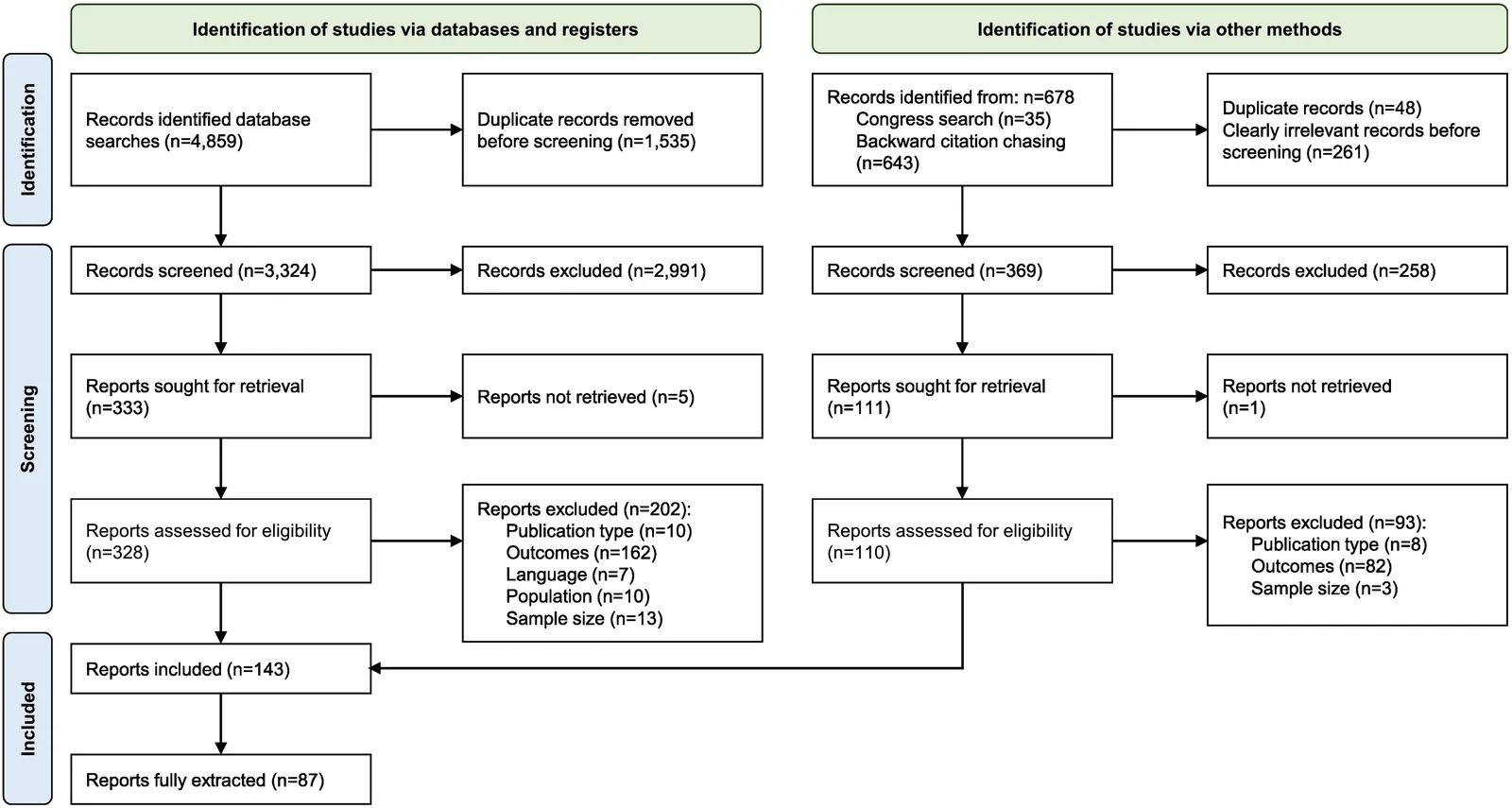
PRISMA diagram of included and excluded studies Abbreviations: PRISMA, Preferred Reporting Items for Systematic Reviews and Meta-Analyses.

Detailed data extraction was not conducted for the remaining 55 studies (listed in **Supplementary Table 12**), as studies that reported numerical values for specified outcomes were prioritised over studies that reported data from which relevant outcomes could be calculated.

### 4.1 Risk of bias assessment

Of the 87 included studies, 85 were assessed using the IHE Quality Appraisal Checklist for Case Series Studies (**Supplementary Table 13**), and two were assessed using the CASP Randomised Controlled Trial Checklist (**Supplementary Table 14**). Twenty-five of the 85 cohort studies were assessed as high quality, three as low quality, and the remainder as medium quality. Most cohort studies were retrospective, did not provide clear eligibility criteria, and were primarily single-centre studies. Low-quality studies generally lacked a precise description of participant selection and did not clearly define outcome measures. Both RCTs were assessed as high quality. One study did not clearly state whether investigators and participants were blinded to interventions.

### 4.2 Fracture characteristics

#### 4.2.1 Age at first fracture

Twenty-four studies reported age at first fracture (Table 2); sample sizes ranged from 10 to 414 participants. Most (19/24) were retrospective studies; the remainder were prospective cohort studies (one included both prospective and retrospective cohorts). Nineteen studies reported a total of 30 distinct ages at first fracture, expressed as mean or median age **(**Fig. 2**)**. The remaining five studies provided categorical data. Most studies (*n* = 20) either did not report OI type or included multiple OI types. Some studies reported age at first fracture for OI Type I (4 studies), OI Type III (2 studies), OI Type IV (2 studies), OI Type V (4 studies), OI Type VI (1 study), OI type VII (1 study), and OI Type XI (1 study). Only one study reported anatomical location of first fracture (Sepulveda et al., 2017).

**Table 2:** Age at first fracture reported in 24 studies.

Report	Study type	n participants	OI Type	Age at first fracture
Numerical data				
Sepulveda et al. (2017)	Retrospective cohort	67	I	Mean 6.7 yrs
Joshi et al. (2023)	Retrospective cohort	44	I	Median (IQR) 1.4 (0.2–2.6) yrs
el-Sobky et al. (2006)	Retrospective and prospective cohorts	13	I	Mean (range) 1.48 (0.17–4) yrs
		11	III	Mean (range) 7.5 (0.08–4) yrs
		14	IV	Mean (range) 1 (0.17–2) yrs
		1	V	0.33 yr
		1	VII	1 yr
Ozturk et al. (2022)	Retrospective cohort	43	I	Median (range) 2 (0.5–10) yrs
		36	III	Median (range) 0.1 (0–1.5) yrs
		3	IV	Median (range) 11.5 (9–13) yrs
		1	V	Median 2 yrs
Tan et al. (2023)	Retrospective cohort	9	V	Mean (range) 1.14 (0–3) yrs
Yuksel Ulker et al. (2021)	Retrospective cohort	12	XI	Median (range) 0.1 (0–1.5) yrs
(Zheng et al. (2022)	Retrospective cohort	20	V	Mean (range) 1.13 (0–3) yrs
Erbas et al. (2022)	Retrospective cohort	29	I, III, IV	Median (IQR) 1.5 (1–3) yrs
Aubry-Rozier et al. (2020)	Prospective cohort	50	I, III, IV, V	Mean (range) 3.8 (0–41) yrs
Harrington et al. (2021)	Retrospective cohort	17	Any	Mean (SD) 1.4 (1.5) yrs
Kizilcan Cetin et al. (2023)	Retrospective cohort	21	I, III, IV, VI	Median (range) 1 (0.08–10) yrs
(Kumar et al. (2023))	Prospective cohort	10	III, IV	Mean (range) 0.98 (0–3) yrs
(Kumar et al. (2016)	Retrospective cohort	29	I, II, III, IV, VII, VIII	Median (range) 3.42 (0.5–6.5) yrs
Li et al. (2011)	Prospective cohort	30	I, III, IV, V	Mean (SD) 2.3 (3.1) yrs
Lin et al. (2024b)	Retrospective cohort	414	I, III, IV, V	Median (range) 1.5 (0–59) yrs
Lv et al. (2016)	Prospective cohort	91	I, III, IV	Mean (SD) 2.3 (2.7) yrs
Mulpuri and Joseph (2000)	Retrospective cohort	9	NR	Mean (SD) 0.7 (0.9) yrs
Mulpuri and Joseph (2000)	Retrospective cohort	7	NR	Mean (SD) 2.7 (2.5) yrs
Wei et al. (2022)	Prospective cohort	56	I, III, IV	Median (IQR) 1.5 (0.11–2.38) yrs
		40	I, III, IV	Median (IQR) 0.5 (0.04–1.5) yrs
Zerfu et al. (2022)	Retrospective cohort	51	NR	Mean (SD) 2.25 (2.01) yrs
		39	NR	Mean (SD) 1.2 (2.12) yrs
		23	NR	Mean (SD) 1.9 (2.4) yrs
Categorical data				
De Wouters et al. (2022)	Prospective cohort	22	I, III, IV, IV	0 to 6 months: 32% 7 to 12 months: 18% 13 to 36 months: 23% 37 months and beyond: 27%
Herdea et al. (2021)	Retrospective cohort	15	I, III	Diagnosed at childbirth: 33.33%
Zhytnik et al. (2019)	Retrospective cohort	143	I, III, IV, V	0 to 1 yrs.: 22.7% 1 to 2 yrs.: 24.8% 3 to 6 yrs.: 17.0% 7 yrs. and beyond: 10.6%
Trejo et al. (2017)	Retrospective cohort	13	VI	0 to 1 yrs.: 69.2%
Tuysuz et al. (2022)	Retrospective cohort	78	I, II, III, IV	Prenatal: 14.1% 0 to 6 months: 34.6% 7 to 23 months: 24.3% 2 to 5 years: 21.8% 6 years and beyond: 5.1%

Abbreviations: IQR, interquartile range; mos, months; NR, not reported; OI, osteogenesis imperfecta; SD, standard deviation; yrs., years.

**Fig. 2:**
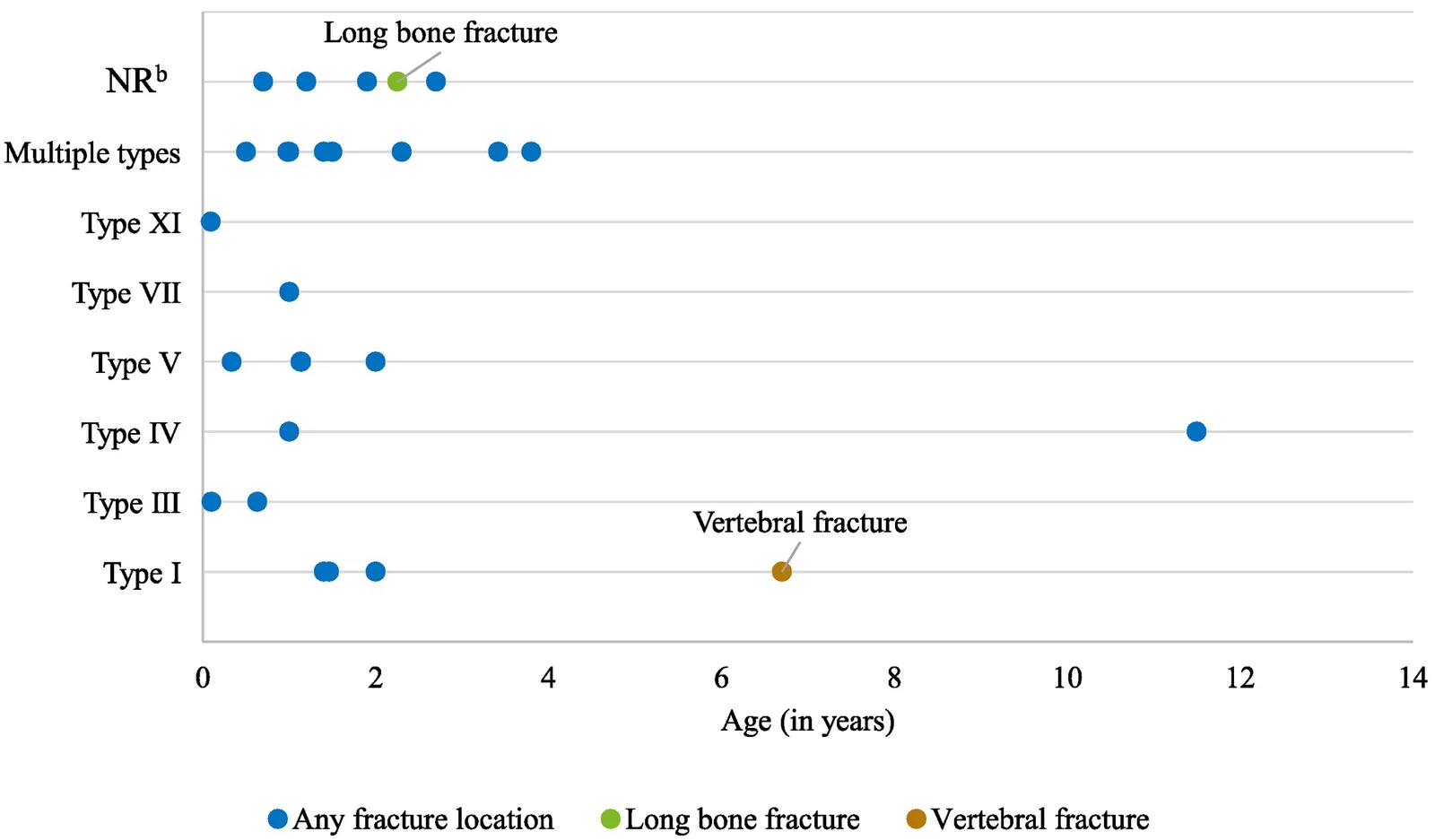
Average age of first fracture by OI type based on 19 studies that reported numerical data (Table 2)^a^ Notes: ^a^From 19 studies that reported numerical data, 28 average ages of first fracture were extracted. Each dot represents an average age of first fracture extracted from the 19 studies. Due to varied reporting by the included studies, datapoints in this graph may represent means, medians or unspecified averages. ^b^Patients' OI type was not reported in these studies. Abbreviations: NR, not reported.

Across OI types, average age at first fracture ranged from 0 to 11.5 years, with most studies reporting an average age of 0–3 years. Average (reported variously as median or mean) ages at first fracture ranged from 1.4 to 6.7 years for OI Type I, 0.1–0.63 years for OI Type III, 1–11.5 years for OI Type IV, and 0.33–2 years for OI Type V. For OI Type XI, the reported median age was 1.2 months (Yuksel Ulker et al., 2021). The youngest age at first fracture was 0.1 years (median), reported by both Ozturk et al. (2022) and Yuksel Ulker et al. (2021), for OI Types III and XI, respectively (Yuksel Ulker et al., 2021; Ozturk et al., 2022). Ozturk et al. (2022) reported the highest age at first fracture for participants with OI Type IV, at 11.5 years (median) (Ozturk et al., 2022).

Overall, the data revealed that people with OI tend to experience their first fracture in early childhood, often before age 3, with earlier onset in more severe OI types such as Type III. However, variability exists. Notably, some individuals with Type I OI fracture in the first year of life, as reported; for example, one study of 44 patients reported a median age at first fracture of 1.4 years (interquartile ratio [IQR], 0.2–2.6 years) (Joshi et al., 2023).

#### 4.2.2 Fracture rates

A total of 70 studies reported fracture rates; 68 were cohort studies and two were RCTs. Most reports (*n* = 48) provided fracture rates pre- or post-treatment, which are pooled in this analysis. Sample sizes ranged from 10 to 861 participants, and average age at enrolment ranged from 0.85 to >65 years.

Reports provided fracture rates in various ways: per year (65 studies reporting 189 rates), per person-year (1 study reporting 2 rates), per 1000 person-years (2 studies reporting 4 rates), or per 100,000 person-years (2 studies reporting 2 rates).

Where reported, per-year fracture rates ranged from 0 to 10.78. Most studies' rates averaged in the 0–4 fractures per year range.

Sixteen studies reported fracture rates by OI type: 12 studies for OI Type I, 9 studies for OI Type III, 9 studies for OI Type IV, 2 studies for OI Type V, 1 study for OI Type VI, and 1 study for OI Type XI. Participants' average age at enrolment ranged from 1.17 years to 39 years. In eight of these studies, participants received treatment and rates were reported before and after treatment or in treated and untreated populations.

Fifteen studies reported per-year fracture rates by OI type (Table 3**,** Fig. 3). These ranged from 0.12 to 3.11 fractures per year for OI Type I, 0.57–10.78 for OI Type III, 0.79–3.9 for OI Type IV, 0.5–1.5 for OI Type V, 1.4–3.1 OI Type VI, and 2–2.8 for OI Type XI.

**Table 3:** Fracture rate by OI type reported in 16 studies.

Report	Study duration	n participants	Age	OI Type	Fracture location	Treatment group	Fracture rate
Per yr							
Ben Amor et al. (2013)	18 yrs	25	Median 8.8 yrs	I	Long bone	Pre-intravenous bisphosphonate	Mean 0.77 per yr
				I		Post-intravenous bisphosphonate	Mean 0.44 per yr
(Bradbury et al. (2012)	2 yrs	32	Mean (range) 39 (18–76) yrs	I	Humerus, radius, ulna, clavicle, pelvis, femur, patella, tibia, fibula, ankle	Pre-risedronate	Mean 0.12 per yr
				I		Post-risedronate	Mean 0.18 per yr
Land et al. (2007)	3 yrs	10	Mean (SD) 7.1 (4.8) yrs	VI	Any	Pre-pamidronate	Mean (SD) 3.1 (0.5) per yr
				VI		Post-pamidronate	Mean (SD) 1.4 (0.4) per yr
Li et al. (2019a)	8 yrs	90	Median (IQR) 11.2 (5–16) yrs	I	Any	NA	Median (IQR) 0.7 (0.3–1.3) per yr
		36	Median (IQR) 12.3 (3.3–19.8) yrs	III		NA	Median (IQR) 1.5 (0.9–2.4) per yr
		35	Median (IQR) 5.8 (4.0–10.7) yrs	IV		NA	Median (IQR) 1 (0.8–1.8) per yr
Lin et al. (2009)	12 yrs	19	Range 2–48 yrs	I	Any	NA	Mean (SD) 0.8 (0.7) per yr
		10	Range 0.2–32 yrs	III		NA	Mean (SD) 4.4 (0.6) per yr
		19	Range 2–53 yrs	IV		NA	Mean (SD) 2 (0.7) per yr
Lindahl et al. (2015))	9 yrs	71	Mean (SD) 9.22 (4.71) yrs	I	Any	NA	Mean (SD) 0.69 (0.85) per yr
		79	Mean (SD) 34.18 (14.98) yrs	I		NA	Mean (SD) 0.48 (0.47) per yr
		15	Mean (SD) 9.27 (4.82) yrs	III		NA	Mean (SD) 4.94 (12.5) per yr
		14	Mean (SD) 36.79 (17.58) yrs	III		NA	Mean (SD) 2.44 (1.66) per yr
		16	Mean (SD) 9.25 (5.64) yrs	IV		NA	Mean (SD) 1.91 (2.02) per yr
		26	Mean (SD) 26.04 (10.35) yrs	IV		NA	Mean (SD) 1 (0.71) per yr
Lindahl et al. (2016)	9 yrs	33	Mean (SD) 8.69 (4.25) yrs	I	Any	Pre-pamidronate	Mean 3.11 per yr
		25	Mean (SD) 3.3 (4.9) yrs	III			Mean 8.05 per yr
		21	Mean (SD) 7.83 (5) yrs	IV			Mean 3.9 per yr
Oduah et al. (2017)	12 yrs	6	NR	I	Any	NA	Mean (SD) 2.2 (0.75) per yr
		36		III		NA	Mean (SD) 3.2 (1.5) per yr
		16		IV		NA	Mean (SD) 2.3 (1) per yr
Ohata et al. (2019)	7 yrs	34	Mean (SD) 10.8 (12.4) yrs	I	Any	NA	Mean (SD) 0.54 (0.36) per yr
		9	Mean (SD) 1.17 (1.62) yrs	III	Any	NA	Mean (SD) 10.78 (10.4) per yr
		10	Mean (SD) 3.61 (4.19) yrs	IV	Any	NA	Mean (SD) 2.88 (5.1) per yr
Rodriguez Celin et al. (2020)	8 yrs	70	Overall study sample: Mean (range) 12.4 (0–67.2) yrs.; subgroup ages NR	III	Femur	Rodded	Mean (range) 0.79 (0.25–2) per yr
		37^a^		III	Femur	Non-rodded	Mean (range) 1.31 (0.33–4) per yr
		99		IV	Femur	Rodded	Mean (range) 0.87 (0.25–2) per yr
		50^a^		IV	Femur	Non-rodded	Mean (range) 0.79 (0.25–1.5) per yr
		61		III	Tibia	Rodded	Mean (range) 0.57 (0.25–1) per yr
		37^a^		III	Tibia	Non-rodded	Mean (range) 0.84 (0.5–1) per yr
		64		IV	Tibia	Rodded	Mean (range) 0.93 (0.33–2.5) per yr
		50^a^		IV	Tibia	Non-rodded	Mean (range) 0.81 (0.25–1.5) per yr
Vuorimies et al. (2011)	5 yrs	17	Median (range) 10.1 (1.5–16.8) yrs	I	Any	Pre-zoledronic acid	Mean 0.52 per yr
		17		I	Any	Post-zoledronic acid	Mean 0.28 per yr
Yazan et al. (2021)	17 yrs	37	NR	I	Any	Pre-pamidronate	Mean (SD) 1.04 (1.26) per yr
		37		I	Any	Post-pamidronate	Mean (SD) 0.33 (0.58) per yr
		15		III	Any	Pre-pamidronate	Mean (SD) 2.39 (1.13) per yr
		15		III	Any	Post-pamidronate	Mean (SD) 0.69 (0.61) per yr
		13		IV	Any	Pre-pamidronate	Mean (SD) 2.21 (1.35) per yr
		13		IV	Any	Post-pamidronate	Mean (SD) 0.83 (0.54) per yr
Yuksel Ulker et al. (2021)	16.8 yrs	12	Median (range) 1.9 (0.6–13.8) yrs	XI	Any	Pre-bisphosphonates	Median (range) 2.8 (0.8–10) per yr
		12		XI	Any	Post-bisphosphonates	Median (range) 2 (0.8–6) per yr
Zeitlin et al. (2006)	10 yrs	11	Mean (range) 8.7 (1.8–15) yrs	V	Any	Pre-pamidronate	Median (range) 1.5 (0.5–3.5) per yr
		11		V	Any	Post-pamidronate	Median (range) 0.5 (0–3) per yr
Zhytnik et al. (2019)	NR	67	Mean (SD) 20.3 (15.9) yrs	I	Any	NA	Median (range) 0.41 (0–2) per yr
		24	Mean (SD) 16.3 (11) yrs	III	Any	NA	Median (range) 1.63 (0.14–30) per yr
		49	Mean (SD) 20 (13.9) yrs	IV	Any	NA	Median (range) 0.83 (0−10) per yr
		3	Mean (SD) 17.7 (15.2) yrs	V	Any	NA	Mean (SD) 0.77 (0.23) per yr
Per 100,000 person-yrs							
Joshi et al. (2023)	12 yrs	44	Mean (IQR) 11.3 (6.2–17) yrs	I	Any	NA	Mean 379 per 1000 person-yrs (95% CI 310–440)

Notes:

Abbreviations: CI, confidence interval; IQR, interquartile range; NA, not assessed; NR, not reported; OI, osteogenesis imperfecta; SD, standard deviation; yr, year.

a N numbers for patients with either femur or tibia fracture.

**Fig. 3:**
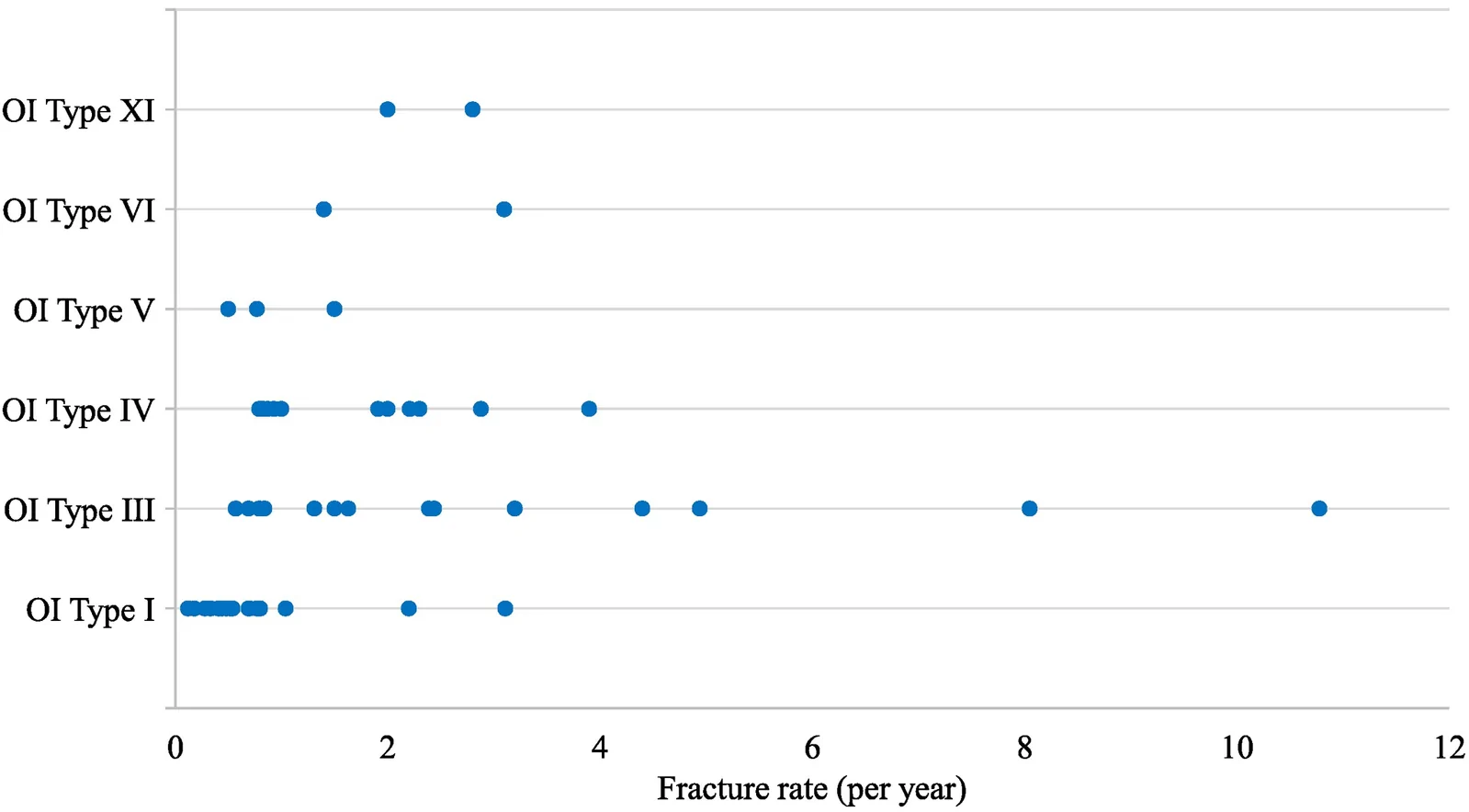
Fracture rates per year by OI type reported in 15 studies (Table 3)^a^ Notes: ^a^From 15 studies, 51 average rates were extracted. Due to varied reporting by the included studies, datapoints in this graph may represent means, medians or unspecified averages. Datapoints also represent a mixture of pre- and post-treatment fracture rates (please see Table 3 for details).

People with OI Type I or Type III experienced fracture most commonly between 0 and 1 and 0.5–5 times per year, respectively. Higher rates were reported by Lindahl et al. (2016; mean 3.11 and 8.05 fractures per year for individuals with OI Type I and Type III, respectively, before pamidronate treatment) (Lindahl et al., 2016) and Ohata et al. (2019; mean 10.78 fractures per year in children with OI Type III and an average age of 1.17 years, all prior to bisphosphonate treatment) (Ohata et al., 2019).

Few studies reported specific fracture rates for OI Types V, VI, or XI. Both reported rates for OI Type VI came from the same study (Land et al., 2007) and represented pre- and post-pamidronate treatment fracture rates (Land et al., 2007).

Overall, the data revealed that people with OI often fracture multiple times per year, and that those with OI Type III tend to fracture at a higher rate than those with OI Type 1 (Table 3**).**

#### 4.2.3 Fracture rates by age

Ten studies reported fracture rates in children and adults; study sizes ranged from 27 to 223 participants. In three studies, fracture rates were reported pre- and post-treatment (Arponen et al., 2015; Bradbury et al., 2012; Xu et al., 2016; Lin et al., 2024a). Seven studies reported a total of 22 annual fracture rates (Table 4. **and** Fig. 4).

**Table 4:** Fracture rates in adults and children reported in 10 studies.

Report	Study duration	n participants	Age group (definition); age	OI Type	Fracture location	Treatment group	Fracture rate
Per year							
Arponen et al. (2015)	NR	27	Children and young adults (≤25 yrs); Range 0–24.8 yrs	I, III, IV, VI, VII	Any	Pre-bisphosphonates	Mean 0.8 per yr
						Post-bisphosphonates	Mean 0.72 per yr
Bradbury et al. (2012)	24 months	27	Adults (≥18 yrs); Mean 39 yrs	I	Humerus, radius, ulna, clavicle, pelvis, femur, patella, tibia, fibula, ankle	Pre-risedronate	Mean 0.12 per yr
						Post-risedronate	Mean 0.18 per yr
Chen et al. (2022)	2 yrs	16	Adults (≥18 yrs); Median 26.7 yrs	I, III, IV, V	Any	NA	Median (IQR) 0.5 (0.2–0.5) per yr
		74	Children (<18 yrs); Median 10.7 yrs				Median (IQR) 1.1 (0.6–1.4) per yr
Lhaneche et al. (2016)	NR	52	Adults (≥20 yrs); Mean 34.96 yrs	NR	Peripheral	NA	aMean (SE) 0.949 (0.098) per yr
							bMean (SE) 0.556 (0.076) per yr
		79	Children (<20 yrs); Mean 9.77 yrs				aMean (SE) 0.84 (0.111) per yr
							bMean (SE) 0.949 (0.099) per yr
Lin et al. (2024a)	12 months	25	Adults (≥18 yrs); Mean 36.7 yrs	I, III, IV	Any	Pre-denosumab^c^	Mean (SD) 0.28 (0.30) per yr
		26	Adults (≥18 yrs); Mean 37.1 yrs			Pre-zoledronic acid^d^	Mean (SD) 0.30 (0.28) per yr
Lindahl et al. (2015)	9 yrs	79	Adults (>18 yrs); Mean 34.18 yrs	I	Any	NA	Mean (SD) 0.48 (0.47) per yr
		14	Adults (>18 yrs); Mean 36.79 yrs	III			Mean (SD) 2.44 (1.66) per yr
		26	Adults (>18 yrs); Mean 26.04 yrs	IV			Mean (SD) 1 (0.71) per yr
		71	Children (≤18 yrs); Mean 9.22 yrs	I			Mean (SD) 0.69 (0.85) per yr
		15	Children (≤18 yrs); Mean 9.27 yrs	III			Mean (SD) 4.94 (12.5) per yr
		16	Children (≤18 yrs); Mean 9.25 yrs	IV			Mean (SD) 1.91 (2.02) per yr
Xu et al. (2016)	24 months	40	Adults (>18 yrs); Mean 30.5 yrs	I, II, III	Any	Pre-alendronate	Mean (SD) 0.46 (0.5)^e^
		33	Adults (>18 yrs); Mean 30.5 yrs			Post-alendronate	Mean (SD) 0.05 (0.22) per yr
		20	Adults (>18 yrs); Mean 35.5 yrs			Pre-zoledronic acid	Mean (SD) 0.53 (0.92)^e^
		19	Adults (>18 yrs); Mean 35.5 yrs			Post-zoledronic acid	Mean (SD) 0.15 (0.49) per yr
Per 100,000 person-yrs							
Goudriaan et al. (2020))	NR	216	Adults (≥16 yrs); Mean 41 yrs	I, III, IV V	Femur	NA	Mean 651 per 100,000 person-yrs
Joshi et al. (2023)	12 yrs	44	Children (≤18 yrs); Mean 11.3 yrs	I	Any	NA	Mean 379 per 1000 person-yrs
Lee et al. (2021)	10 yrs	72	Adults (>18 yrs); Mean 41 yrs	I, III, IV	Femur	NA	Mean 651 per 100,000 person-yrs

Notes:

Abbreviations: IQR, interquartile range; NA, not applicable; NR, not reported; OI, osteogenesis imperfecta; SD, standard deviation; SE, standard error; yr, year.

a Rate only includes fractures before the age of 10 years.

b Rate includes fractures over participants' lifetime.

c 17 individuals previously received treatment (9 with alendronate, 9 with zoledronic acid and 3 with teriparatide).

d 16 individuals previously received treatment (11 with alendronate, 8 with zoledronic acid and 1 with teriparatide).

e Time period for pre-treatment NR.

**Fig. 4:**
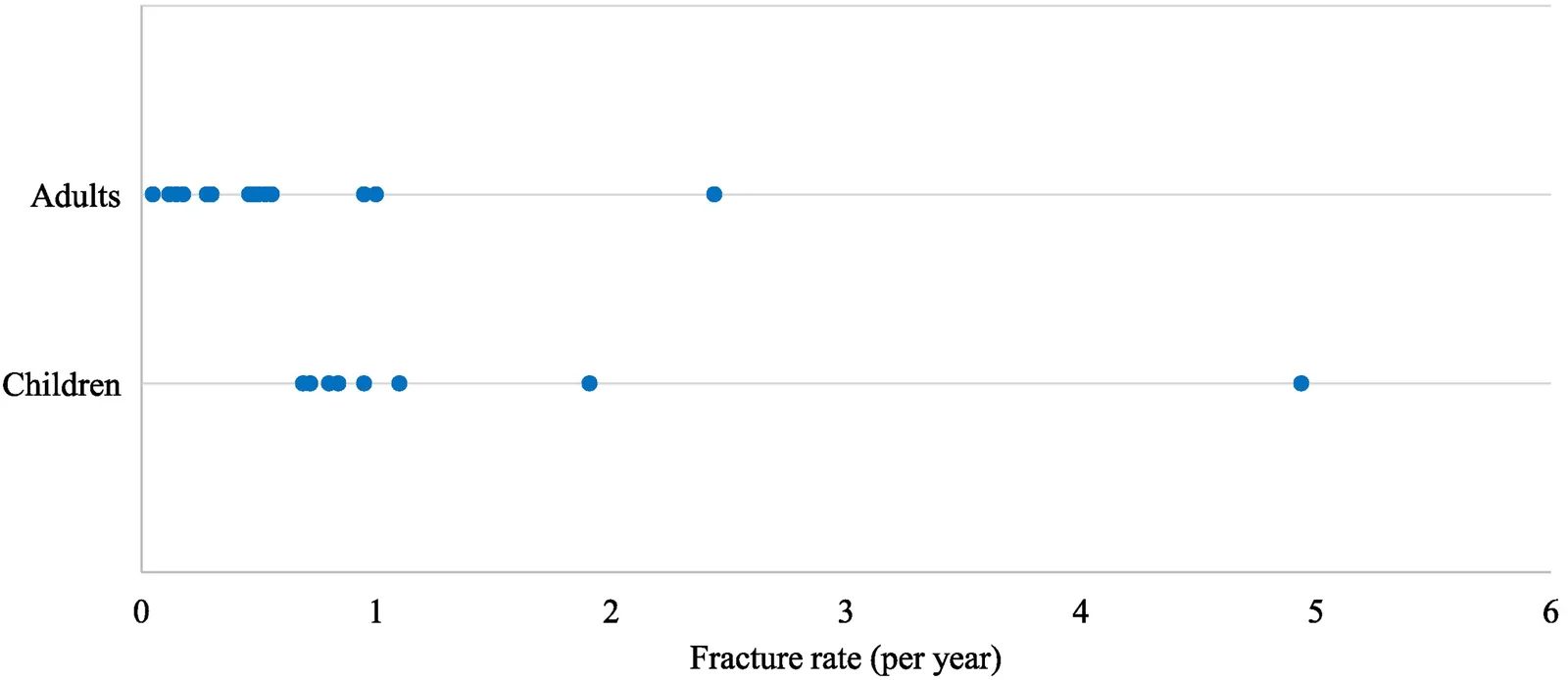
Fracture rates for adults and children per year, reported in 7 studies (Table 4)^a^ Notes: ^a^From 7 studies, 22 average rates were extracted. Due to varied reporting by the included studies, datapoints in this graph may represent means, medians or unspecified averages. Datapoints also represent a mixture of pre- and post-treatment fracture rates (please see Table 4 for details).

Average fracture rates for adults and children ranged from 0 to 2.44 and 0.69–4.94 times per year, respectively. The highest reported rates (mean 2.44 and 4.94 fractures per year in adults and children, respectively) were reported in people with OI Type III. In one of those studies, Lindahl et al. (2015), the 15 participants' mean of 4.94 fractures per year carried a high standard deviation of 12.5 fractures per year (Lindahl et al., 2015).

#### 4.2.4 Fracture location

Fifteen studies reported fracture rates by anatomic location: vertebrae (2 studies), long bones (6 studies), tibiae (1 study), femurs (3 studies), and other locations (6 studies). Study populations ranged from 10 to 216 participants, with ages averaging 14 months to 41 years at enrolment. Nine of these studies reported pre- and post-treatment fracture rates.

Most studies reported fracture rates ranging from 0 to 2 fractures per year across locations. Thirteen studies reported per-year fracture rates; these ranged from 0 to 8 fractures per year. Two studies reported fracture rates per patient-years, and two provided fracture rates for specific locations per 100,000 person-years.

The range of fracture rates was 0–1.8 fractures per year for vertebrae, 0.04–8 for long bones, 0.57–0.93 for tibiae, 0.79–1.31 for femurs and 0.12–2.3 for other locations.

While most studies reported fewer than four long bone fractures per year, Alcausin et al. (2013) reported an unusually high median rate of eight per year pre-pamidronate treatment in a group of 17 children with moderate-to-severe OI (Alcausin et al., 2013).

Overall, no clearly predominant anatomical location was observed in the reported fracture rates; however, this finding should be interpreted with caution given heterogeneity in study design and reporting (Table 5**,** Fig. 5).

**Table 5:** Fracture rate by fracture location reported in 15 studies.

Report	Study duration	n participants	Age	OI Type	Fracture location	Treatment group	Fracture rate
Per yr							
Alcausin et al. (2013)	9 yrs	17	Mean (SD) 14 (11) months	I, III, V	Long bones	Pre-pamidronate	Median (range) 8 (2–77) per yr
						Post-pamidronate^a^	Median (range) 1 (0–5) per yr
						Post-pamidronate^b^	Median 1.4 per yr
Ben Amor et al. (2013)	18 yrs	25	Median 8.8 yrs	I	Long bones	Pre-intravenous bisphosphonates	Mean 0.77 per yr
						Post-intravenous bisphosphonates	Mean 0.44 per yr
Biggin et al. (2015)	NR	17	Mean (SD) 4.8 (2.6) yrs	I, III, IV	Long bones and vertebrae	Pre-bisphosphonates	Mean (SD) 1.5 (1.1) per yr
						Post-bisphosphonates	Mean (SD) 0.7 (0.7) per yr
Bradbury et al. (2012)	24 months	27	Mean (range) 39 (18–76) yrs	I	Humerus, radius, ulna, clavicle, pelvis, femur, patella, tibia, fibula, ankle	Pre-bisphosphonates	Mean 0.12 per yr
						Post-bisphosphonates	Mean 0.18 per yr
Franzone et al. (2017)	22 yrs	24	Mean (range) 8.7 (3.1–19.2) yrs	I, III	Long bones	Pre-intramedullary rodding	Mean (SD) 0.87 (0.47) per yr
						Post-intramedullary rodding	Mean (SD) 0.1 (0.6) per yr
Huang et al. (2006)	2 yrs	20	Mean (SD) 10.6 (3.4) yrs	I, III, IV	Spine, wrist, femur	NA	Mean (SD) 0.82 (0.6) per yr
Lhaneche et al. (2016)	NR	79	Mean (SE) 9.77 (0.42) yrs	NR	Peripheral (defined by authors as any non-vertebral fracture)	NA	cMean (SE) 0.84 (0.111) per yr
							dMean (SE) 0.949 (0.099) per yr
		52	Mean (SE) 34.96 (2.07) yrs				cMean (SE) 0.949 (0.098) per yr
							dMean (SE) 0.556 (0.076) per yr
Li et al. (2019b)	2 yrs	10	Mean (SD) 8.9 (4.2) yrs	I, III, IV	Non-vertebral compression	Pre-zoledronate	Mean (range) 0.9 (0.5–2.6) per yr
		32	Mean (SD) 8.8 (3.7) yrs		Vertebral compression		Mean (range) 0.8 (0.5–1.5) per yr
Mulpuri and Joseph (2000)	10 yrs	9	Mean (SD) 6.3 (3.8) yrs^e^	NR	Long bones	Pre-elongating rod	Mean 2.63 per yr (95% CI 2.24–3.02)
		7	Mean (SD) 6.5 (3.5) yrs^e^			Pre-non-elongating rod	Mean 1.58 per yr (95% CI 1.29–1.86)
		9	Mean 6.3 (SD) (3.8) yrs^e^			Post-elongating rod	Mean 0.04 per yr (95% CI 0.02–0.09)
		7	Mean (SD) 6.5 (3.5) yrs^e^			Post-non-elongating rod	Mean 0.21 per yr (95% CI 0.14–0.27)
Poyrazoglu et al. (2008)	6 yrs	35	Mean (SD) 5.9 (4.6) yrs	I, III, IV	Long bones^f^	Pre-pamidronate	Mean (SD) 2.13 (2) per yr
						Post-pamidronate	Mean (SD) 0.82 (1.2) per yr
Saraff et al. (2018)	14 yrs	20	Mean 9.17 yrs	I, IV	Long bones	Pre-pamidronate	Mean (SD) 3.5 (1.2) per yr
		20	Mean 9.17 yrs			Post-pamidronate	Mean 1.5 per yr
		20	Mean 9.7 yrs			Pre-zoledronate	Mean (SD) 2.8 (1.49) per yr
		20	Mean 9.7 yrs			Post-zoledronate	Mean 1.0 per yr
		20	Mean 9.17 yrs		Vertebrae	Pre-pamidronate	Mean (SD) 1.8 (0.89) per yr
		20	Mean 9.17 yrs			Post-pamidronate	Mean 0 per yr
		20	Mean 9.7 yrs			Pre-zoledronate	Mean (SD) 1.75 (1.07) per yr
		20	Mean 9.7 yrs			Post-zoledronate	Mean 0 per yr
Rodriguez Celin et al. (2020)	8 yrs	70	Overall study sample: Mean (range) 12.4 (0–67.2) yrs.; subgroup ages NR	III	Femur	Rodded	Mean (range) 0.79 (0.25–2) per yr
		37^g^		III		Non-rodded	Mean (range) 1.31 (0.33–4) per yr
		99		IV		Rodded	Mean (range) 0.87 (0.25–2) per yr
		50^g^		IV		Non-rodded	Mean (range) 0.79 (0.25–1.5) per yr
		61		III	Tibiae	Rodded	Mean (range) 0.57 (0.25–1) per yr
		37^g^		III		Non-rodded	Mean (range) 0.84 (0.5–1) per yr
		64		IV		Rodded	Mean (range) 0.93 (0.33–2.5) per yr
		50^g^		IV		Non-rodded	Mean (range) 0.81 (0.25–1.5) per yr
Choi et al. (2007)	37 months	11	Mean (SD) 5.9 (8.11) yrs	I, III	Non-vertebral compression	Pre-pamidronate	Mean (SD) 2.3 (1.01) per yr
						Post-pamidronate	Mean (SD) 0.6 (0.69) per yr
Per 100,000 person-yrs							
Goudriaan et al. (2020)	NR	216	Mean (SD) 41 (15.3) yrs	I, III, IV, V	Femur	NA	Mean 651 per 100,000 person-yrs
Lee et al. (2021)	10 yrs	72	Mean (SD) 41 (15.3) yrs	I, III, IV	Femur	NA	Mean 651 per 100,000 person-yrs

Notes:

Abbreviations: CI, confidence interval; NA, not applicable; NR, not reported; OI, osteogenesis imperfecta; SD, standard deviation; SE, standard error; yr, year.

a Calculated one year after treatment.

b Calculated two years after treatment.

c Rate only includes fractures before the age of 10 years.

d Rate includes fractures over participants' lifetime.

e Age at primary surgery.

f Included patients with long bone deformities and/or recurrent fractures.

g N numbers for patients with either femur or tibia fracture.

**Fig. 5:**
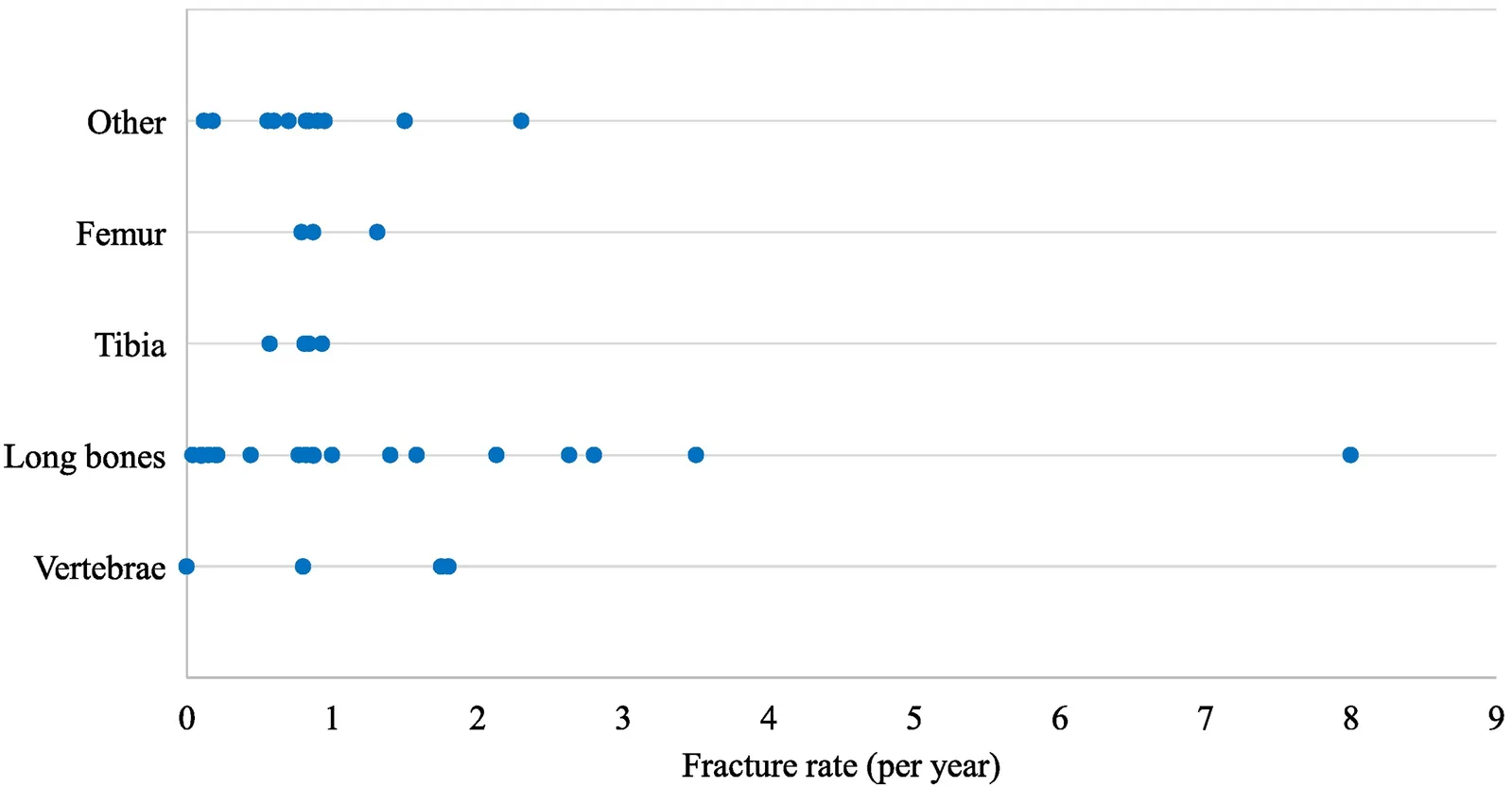
Fracture rates per year by fracture location, reported in 13 studies^a^ Notes: ^a^From 13 studies, 42 average rates were extracted. Due to varied reporting by the included studies, datapoints in this graph may represent means, medians or unspecified averages. ^b^‘Other’ includes ‘peripheral,’ ‘non-vertebral compression,’ ‘long bone and vertebral,’ ‘spine, wrist and femur,’ and ‘humerus, radius, ulna, clavicle, pelvis, femur, patella, tibia, fibula and ankle fractures’.

#### 4.2.5 Fracture risk factors

Evidence on risk factors for fracture frequency was limited. Beyond age and OI type, no additional risk factors were consistently identified across studies. Available data suggest that younger age and more severe OI types are associated with higher fracture frequency; however, these findings should be interpreted with caution given heterogeneity in study design and reporting.

### 4.3 Fracture outcomes

#### 4.3.1 Fracture healing time

Just four studies reported healing time after fractures (**Supplementary Table 15**). Sample sizes ranged from 12 to 22 participants, and the definition of healing varied across studies. Five healing times were extracted from four studies and ranged from 4 to 8 weeks (Abulsaad and Abdelrahman, 2009; Puvanesarajah et al., 2015; Bhaskar and Khurana, 2019; Johnson and Varghese, 2008). Three studies reported healing times after surgical interventions (Abulsaad and Abdelrahman, 2009; Puvanesarajah et al., 2015; Bhaskar and Khurana, 2019), while one assessed healing times without any surgical intervention in femurs, tibiae, fibulae, radii, and humeri as well as unspecified limb bones (Johnson and Varghese, 2008). In two studies of individuals treated with intramedullary rodding, the reported healing time of femurs and tibiae was 7–8 weeks (Abulsaad and Abdelrahman, 2009; Bhaskar and Khurana, 2019). Lastly, in a study reporting healing time after compressed sandwich allograft cortical struts in radii, fibulas, femurs, humeri, time to clinical union was 5.25 weeks (Puvanesarajah et al., 2015). Overall, data on healing time were limited, precluding robust conclusions.

#### 4.3.2 Downstream events

Three studies described downstream SSEs of fractures for people with OI Types I, III, or IV (Joshi et al., 2023; Zack et al., 2005; Vyskocil et al., 2005). Zack et al. (2005) contacted a random sample of 79 children with OI, mostly of Types I, III, or IV, asking them about pain associated with their most recent fracture as well as non-fracture related chronic pain (Zack et al., 2005). Of them, 35 returned the surveys, and 34 reported having experienced a total of 48 fractures. Of those fractures, 48% had occurred in the last 12 months; mean time since the last fracture was 1.72 years (standard deviation, 1.64). For each activity of daily living, the majority of those 34 participants (73–97%) reported limitations due to fracture pain (**Supplementary Table 16**).

Vyskocil et al. (2005) conducted a prospective study of alendronate in 30 individuals with OI Types I, III or IV. They found a significant negative relationship between fracture rate and mobility and a positive correlation between fracture rate and pain; the latter was significant before alendronate therapy and non-significant after 1 year of alendronate (Vyskocil et al., 2005).

A retrospective cohort study by Joshi et al. (2023) characterised fractures in children with OI at different stages of bone growth and found that 26 of 197 total fractures occurred at the same anatomical site as a previous fracture (Joshi et al., 2023). Comparative data from the general population are lacking, limiting contextual interpretation.

Overall, limited evidence suggests that fractures in individuals with OI are associated with pain and may substantially impair daily activities, including sleep, mobility, travel, occupation and leisure.

## 5 Discussion

To our knowledge, this systematic review is the first to comprehensively collate published evidence on the clinical burden of fractures among people with OI. Specifically, it captured data on the age at first fracture, fracture frequency, fracture healing time, and downstream events associated with fractures.

The evidence reported in the included studies indicates that people with OI begin fracturing early in life, with those with more severe phenotypes, particularly Type III, generally experiencing the highest fracture burden. Multiple fractures per year are common, and some occur at previously affected sites. No clearly predominant anatomical location was identified; however, this likely reflects heterogeneity in study design and reporting rather than true absence of differences. Evidence on fracture healing time remains limited. Fractures appear to be associated with substantial pain and impairment in daily functioning.

People with OI typically begin experiencing fractures in early childhood (Lin et al., 2009). Our review found that people with OI often experience their first fracture before the age of 3 years, with earlier onset observed in more severe OI types, often within the first year of life in Type III. These observations are broadly consistent with clinical experience, in which fracture rates are typically higher in childhood and in more severe phenotypes such as Type III (Marini and Dang Do, 2020). However, substantial variability exists across the lifespan and among individuals, particularly in the context of treatment and disease severity.

Although early fractures are a key clinical feature of OI, they do not always lead to early diagnosis. Individuals with milder phenotypes may experience delayed diagnosis, particularly if fractures are infrequent or occur later in life (Nguyen et al., 2022; Drari et al., 2026). Fracture patterns, such as early vertebral fractures or recurrent fractures, may help prompt clinical evaluation for OI.

Underscoring the high unmet need for effective treatment in the OI population, we confirmed that OI often results in multiple fractures annually. We found that average yearly fracture rates ranged from 0 to 10.78 across studies, with most falling between >0 and 4 fractures per person per year across all OI types. This represents a considerably higher burden than estimates in the general population, which one report put at 0.38 fractures per 100,000 people per year (Donaldson et al., 2008). Such frequent injury in people with OI has been linked to increased healthcare utilisation and economic impact (Abraham et al., 2025; Hart et al., 2024; Rauch et al., 2025).

Interestingly, fracture rates did not appear substantially different across OI types in the studies we collated. Most people with OI experience a significant number of fractures throughout their life, regardless of OI type. Still, wide variability exists; for example, people with OI Type III tend to fracture more often than those with OI Type I, and even people with the same variant or in the same family may experience different fracture rates, as many factors such as age, treatment history, activity level and anatomical characteristics can influence fracture rate. The included studies suggest that some individuals with OI types considered milder may experience fractures at least as frequently as those with OI types considered more severe. Such complexities may help to explain why fracture rates appeared roughly comparable across OI types.

With respect to fracture location, no consistent pattern emerged across studies. Although some literature suggests that long bones, particularly the femur, are frequently affected (Goudriaan et al., 2020; Ben Amor et al., 2013), this may reflect reporting bias, as more clinically significant fractures are more likely to be captured. Other ‘minor’ fractures may be underreported, as people may choose to forgo medical attention. For example, vertebral fractures are a typical manifestation of bone fragility, but appeared to be underrepresented in the included studies, possibly due to under-detection or inconsistent reporting. Disparities in study design may also affect data collection on fracture location, such as observation periods, data sources, fracture definitions, and patient characteristics.

The fracture burden in OI is lifelong. While children tend to experience fractures at higher rates than adults and many patients experience substantial alleviation of fracture burden, some people with OI continue to suffer fractures throughout their lifetimes. The highest reported fracture rates were reported among both adults (mean 2.44 fractures per year) and children (mean 4.94 fractures per year) with OI Type III. This phenomenon echoes that of the OI-unaffected general population wherein children and adolescents tend to experience more fractures than adults. However, in the general population, older adults sustain increasing fractures rates largely due to age-associated factors summarised under the umbrella term osteoporosis, and this may partially overlap with fracture rates due to OI, specifically in milder affected OI patients. Accurate data on fracture rates in very old patients with severe OI are therefore limited. In summary, fracture patterns across the lifespan may vary, and differences compared with the general population are influenced not only by disease severity, behavioural adaptations, and treatment, but also overlapping risk factors determining fracture risk in the general population. Notably, fractures in OI may occur following minimal trauma, and individuals often modify activities to reduce injury risk, which may influence observed fracture rates (Hill et al., 2019; Colombo et al., 2025). Many fractures in OI may occur due to seemingly trivial forces that would be insufficient to injure people without OI (Marini and Dang Do, 2020; Yotsuya et al., 2023).

Importantly, detecting fractures among children can pose a diagnostic challenge, with sometimes subtle findings on X-rays (George and Bixby, 2019). This suggests that reported fracture rates in children could be underestimates. Additionally, children and adolescents may not seek medical consultation for minor or non-displaced fractures, especially in severe forms of OI when individuals are used to frequent fractures. OI-associated fractures among adults may also be missed due to self-management of fractures and therefore lack of reporting, potential lack of support post-transition from paediatric services resulting in loss to follow-up, and other reasons for not attending healthcare (Picaro et al., 2025; Michalovic et al., 2020). These factors suggest that reported fracture rates may underestimate the true burden, both in children and adults.

Healing time remains unclear, with variable definitions of healing across the four studies that reported this outcome. Moreover, healing time in people with OI may depend on many factors, including OI type, age, bone type, and surgical intervention as well as obesity, endocrine conditions, and history of bisphosphonates or other treatments (Puvanesarajah et al., 2015; Shen et al., 2022; Sheen et al., 2026; Etich et al., 2020). A glimpse of possibly typical healing times is afforded by two studies of individuals treated with intramedullary rodding, whose reported healing times for femurs and tibiae were comparable at 7–8 weeks (Abulsaad and Abdelrahman, 2009; Bhaskar and Khurana, 2019). Other studies report shorter times in other limbs or with sandwich allograft struts, but overall, the heterogeneity and scarcity of data make broad conclusions difficult. Interestingly, although OI may increase susceptibility to fractures, some evidence has suggested that people with OI heal at similar or faster rates than the general population due to higher vascularity and cellularity in OI bones (Nijhuis et al., 2022).

Only three included studies reported numerical data on downstream events and SSEs among OI patients, limiting our ability to describe the frequency of such events. Still, from the modest data available, fracture in OI appears profoundly burdensome. Fracture pain may limit aspects of daily life, such as sleep, travel, occupational and leisure activities. Fracture rate was negatively correlated with mobility status, regardless of treatment. Evidence suggests that fractures affect both physical health and well-being, and they can impact many aspects of daily living (van Welzenis et al., 2024; Zack et al., 2005). In an effort to address large evidence gaps relating to the humanistic, economic, and clinical effects of OI, the IMPACT survey asked 1440 adults and 92 adolescents with OI and their caregivers (*n* = 560) and relatives (*n* = 116) about these effects. Some 84% of adults reported that OI affected their leisure activities and 74% reported that it reflected the kind of jobs they could perform (van Welzenis et al., 2024).

A fracture may recur in the same anatomical site: Joshi et al. (2023) suggest that previous fractures in people with OI could potentially predict the likelihood of a fracture reoccurring at that location (Joshi et al., 2023). Higher refracture risk is also seen in populations not affected with OI, such as among children with diaphyseal both-bone forearm fractures (Pakarinen et al., 2025).

### 5.1 Limitations

Most studies included in the review were retrospective, with data primarily derived from patient records that may be incomplete or inaccurate. Our search strategy did not attempt to capture rates of prenatal fracture or non-union, both of which can occur in OI.

The included studies may not be representative of the broader OI population. Study participation required a confirmed diagnosis, which typically depends on access to specialist care. Individuals with milder phenotypes or limited access to healthcare may therefore be underrepresented, while those with more severe disease may be overrepresented due to more frequent clinical follow-up and more comprehensive medical records. Similarly, children—who are more likely to receive regular, multidisciplinary care—may be disproportionately represented compared with adults. Many fractures may not have been captured due to self-management of less severe fractures, for which people do not seek medical help.

Fractures are difficult to record in a standardised manner across studies, which results in a major limitation to our review. Our selected studies used a variety of methods for recording fractures, including parent interviews, medical record reviews and radiographic images. This introduces potential sources of error or disparities in fracture counts. Additional heterogeneity was observed in study design, including variation in follow-up duration, fracture definitions, and reporting of treatment exposure. Some studies reported lifetime fracture counts, whereas others assessed fractures over limited time periods, further complicating comparisons. Many studies were not designed to compare fracture rates across anatomical locations, limiting confidence in conclusions regarding fracture distribution.

Certain variables outside of the primary variable of interest could not be controlled for. These included differing ongoing or past treatment, different definitions of healing time, different OI types evaluated across studies, lack of reporting on OI types, and different patient age groups evaluated. In our graphs depicting fracture rates, we did not indicate which data points represent pre-treatment versus post-treatment values, as the focus of our review was on fracture frequency rather than treatment. This makes drawing inferences from our data difficult. Moreover, each graph prioritised a single variable related to our research questions: OI type, fracture location, and age. While this was done for clarity, interactions between variables could not be assessed.

Our ability to parse risk factors was limited to age and OI type, and even about those the data permitted only broad and tentative conclusions. Although we attempted to stratify fracture rates by OI type, we do not have information on fracture rates by affected gene or gene variant. Other social factors that influence fracture rates were not accounted for: for example, boys, particularly adolescents, tend to experience more fractures than girls due to more participation in activities like contact sports and greater risk-taking behaviour (Oh et al., 2022; Moustaki et al., 2001). Older women may fracture more than older men due to post-menopausal osteoporosis (Karlamangla et al., 2018). We did not attempt to parse sex-related differences in this review. Finally, studies generally did not capture whether a fracture was traumatic or non-traumatic, nor the risk-limiting measures that some people with OI may take to avoid fracture.

This review prioritised breadth over comparability, and therefore only qualitative synthesis was possible. Future studies of OI fracture rates should include prospective observational studies that combine self-reported data with clinical records and evaluate multiple factors that could influence fracture risk.

## 6 Conclusions

Despite gaps in the literature, this review clearly confirms the substantial fracture burden experienced by people with OI, irrespective of age or OI type. The available data depict early, frequent, lifelong, and burdensome fractures in people with OI. The true burden is likely underestimated, given heterogeneity in study design, incomplete fracture ascertainment, and limited reporting of downstream outcomes. More information is needed on multiple topics, including fracture healing times, downstream events and SSEs, as well as diagnostic delay. Efforts are underway to address these knowledge gaps, including the SATURN Programme (Systematic Accumulation of Treatment practices and Utilisation, Real world evidence, and Natural history) (Sangiorgi et al., 2024) and a large longitudinal study by the Brittle Bone Disorders Consortium (Brittle Bone Disorders Consortium, 2025).

Our findings underscore the urgent need for effective, targeted OI interventions to reduce fracture risk and support fracture management, resulting in improved long-term clinical outcomes for those with OI. A more comprehensive understanding of the relationship between fracture burden and disability, chronic pain, healthcare utilisation, and psychosocial and economic impact is essential to inform clinical care, research priorities, and health policy.

## CRediT authorship contribution statement

**Lothar Seefried:** Writing – review & editing, Conceptualization. **Rosa Bou:** Writing – review & editing, Conceptualization. **Judith Bubbear:** Writing – review & editing. **Ruggero Lanzafame:** Writing – review & editing. **Thomas Zoller:** Writing – review & editing. **Samantha Prince:** Writing – review & editing, Conceptualization. **Maria Rapoport:** Writing – review & editing, Writing – original draft, Methodology, Formal analysis, Data curation, Conceptualization. **Ogün Sazova:** Writing – review & editing, Conceptualization. **Clive Whitcher:** Writing – review & editing, Conceptualization. **Franco Antoniazzi:** Writing – review & editing.

## Ethics approval and consent to participate

Not applicable.

## Funding

Funding for this research was provided by Mereo BioPharma Group, London, United Kingdom.

## Declaration of competing interest

Lothar Seefried has received grants or contracts from Alexion/AstraZeneca, Chiesi and KyowKirin, paid to his institution, and honoraria for lectures from Alesta, Alexion/AstraZeneca, Amgen, AM-Pharma, Biomarin, Chiesi, Inozyme, Ipsen, KyowaKirin, medi, Mereo, Stadapharm, Theramex, UCB and Ultragenyx. Rosa Bou received contracts for participation in clinical trials from Amgen, paid to her institution, and honoraria for lectures/support for attending meetings from Mereo BioPharma. Judith Bubbear has received personal honoraria/speaker fees from UCB and Amgen. She is an unpaid member of the medical advisory board of the Brittle Bone Society, has received support for meeting attendance from UCB and participated in an advisory board for Mereo Biopharma Group. She is a principal investigator of the MOI-A study of losartan in OI, and a sub-investigator in the TOPAZ study. For both non-commercial research studies payments were made to her institution. Thomas Zoller has received consulting fees from Mereo Biopharma Group. Ruggero Lanzafame has no conflicts to disclose. Maria Rapoport is an employee of Wickenstones Ltd. Samantha Prince was an employee of Mereo BioPharma Group when this systematic review was conceived. Ogün Sazova and Clive Whitcher are employees and shareholders of Mereo Biopharma Group. Franco Antoniazzi has received grants from Merck Serono and Sandoz as part of his work at the University of Verona; he has received personal consulting fees from Mereo BioPharma Group and support for attending meetings/travel from Kyowa Kirin and Novo Nordisk. He participated in advisory boards for Biomarin and Ascendis Pharma.

## Data Availability

No data was used for the research described in the article.
