# Supplementary material for: Fracture characteristics and outcomes in osteogenesis imperfecta: A systematic review
Source: Bone Rep. 2026 Oct 10;31:101958. doi: 10.1016/j.bonr.2026.101958 (PMC13694420; doi:10.1016/j.bonr.2026.101958)
Supplement: Supplementary file 1 [file mmc1.docx]

# Supplementary material

## Supplementary Tables

Supplementary Table 1. Search strategy for MEDLINE (via Ovid), on 18 March 2024 (date limit 1946–15 March 2024)

| 1 Osteogenesis imperfecta/ (5260) 2 ((osteogenesis adj2 (imperfecta or fragil*)) or (imperfecta adj2 ossium) or (Brittle adj3 Bone*) or (fragile adj2 bone*) or (fragility adj2 bone*) or (glass adj2 bone*) or glassbone* or "soft bone*1" or bruck syndrome or osteopsathyrosis or "periostal aplasia" or (lobstein* adj2 (disease or syndrome*)) or ((imperfecta or fragil*) adj2 (ossium* or vitera))).ti,ab,kf. (9426) 3 ((broken or break or breaking or fracture*1 or fracturing) adj5 (frequently or often or easily or easy or regular or regularly or constantly or continually or continuously routinely or repeatedly or "over and over" or always or recurrent) adj5 bone*).ti,ab,kf. (888) 4 1 or 2 or 3 (10860) | **Condition terms:** The search opens at Line 1 with the controlled indexing (MeSH) term for osteogenesis imperfecta (the / indicates that this is an indexing term).  At Lines 2−3 we utilise free-text terms. These are terms identified by the research team. They seek to cover deficiencies in indexing, to identify new or unindexed records, and to search in content fields beyond indexing.  This search uses terms for both osteogenesis imperfecta or brittle bones, as variation in naming is noted in registry reports (c.f. ^1^or ^2^). In the clinical searches, we take a sensitive search for the condition, seeking to minimise the risk of missing potentially eligible studies.  We do not distinguish between or define types of osteogenesis imperfecta (e.g., Type 1, etc.) as the underlying condition term does not change by type (c.f.^3-5^). That is, there is not a different sub-name by type, as is the case for some conditions.  Free-text terms are searched in the following fields:  ti = title  ab = abstract  kf = author generated keyword filed (these are terms chosen by authors to describe their papers).    We use proximity markers and truncation:  * proximity markers (e.g., adj2) allow us to search between search terms to a gap of one space and with the terms in either order. So we will identify ‘brittle leg bone’ or ‘bones were brittle’.  * Truncation (indicated by *) allows us to identify a word root (e.g., fragil) and then variant word endings (e.g., fragile, fragility, fragilitas).  We checked the search strategies and studies included or excluded in the two Cochrane reviews focused on osteogenesis imperfecta.^6,7^ The aim was to critique our draft condition search terms to see if any terms had been overlooked. We also scoped the literature and studies. This process did not lead us to alter our terms. |
| --- | --- |
| 5 (Randomized Controlled Trial or Controlled Clinical Trial or Pragmatic Clinical Trial or Equivalence Trial or Clinical Trial, Phase III).pt. (705918) 6 Randomized Controlled Trial/ (610088) 7 exp Randomized Controlled Trials as Topic/ (171507) 8 "Randomized Controlled Trial (topic)"/ (0) 9 Controlled Clinical Trial/ (95585) 10 exp Controlled Clinical Trials as Topic/ (177230) 11 "Controlled Clinical Trial (topic)"/ (0) 12 Randomization/ (107058) 13 Random Allocation/ (107058) 14 Double-Blind Method/ (177775) 15 Double Blind Procedure/ (0) 16 Double-Blind Studies/ (177775) 17 Single-Blind Method/ (33313) 18 Single Blind Procedure/ (0) 19 Single-Blind Studies/ (33313) 20 Placebos/ (35934) 21 Placebo/ (0) 22 Control Groups/ (2111) 23 Control Group/ (2111) 24 (random* or sham or placebo*).ti,ab,hw,kf,kw. (1889198) 25 ((singl* or doubl*) adj (blind* or dumm* or mask* or arm or arms)).ti,ab,hw,kf,kw. (288650) 26 ((tripl* or trebl*) adj (blind* or dumm* or mask*)).ti,ab,hw,kf,kw. (1787) 27 (control* adj3 (study or studies or trial* or group*)).ti,ab,kf,kw. (1283798) 28 (Nonrandom* or non random* or non-random* or nRCT or quasi-random* or quasirandom*).ti,ab,hw,kf,kw. (57451) 29 allocated.ti,ab,hw. (88046) 30 ((open label or open-label) adj5 (study or studies or trial* or extension)).ti,ab,hw,kf,kw. (48039) 31 ((sub* and (group adj2 anal*)) or (subgroup adj2 anal*)).ti,ab,kw,kf. (86799) 32 ((equivalence or superiority or non-inferiority or noninferiority) adj3 (study or studies or trial*)).ti,ab,hw,kf,kw. (13138) 33 (pragmatic study or pragmatic studies).ti,ab,hw,kf,kw. (639) 34 ((pragmatic or practical) adj3 trial*).ti,ab,hw,kf,kw. (8277) 35 ((quasiexperimental or quasi-experimental) adj3 (study or studies or trial*)).ti,ab,hw,kf,kw. (13446) 36 ("Phase 3*" or "phase3*" or "phase III*" or P3* or "PIII*" or "Phase 2*" or "phase2*" or "phase II*" or P2* or "PII*").ti,ab,kw,kf. (404939) 37 (trial or trail).ti,ab,kw,kf. (828205) 38 5 or 6 or 7 or 8 or 9 or 10 or 11 or 12 or 13 or 14 or 15 or 16 or 17 or 18 or 19 or 20 or 21 or 22 or 23 or 24 or 25 or 26 or 27 or 28 or 29 or 30 or 31 or 32 or 33 or 34 or 35 or 36 or 37 (3332618) 39 Epidemiologic studies/ (9510) 40 exp case control studies/ (1489488) 41 exp cohort studies/ (2583638) 42 Case control.tw. (160578) 43 cohort*1.ti,ab,kf. (926432) 44 (Follow up adj (study or studies)).tw. (57806) 45 observational study.pt. or (observational adj (study or studies)).tw. (263337) 46 Longitudinal.tw. (340916) 47 Retrospective.tw. (798010) 48 (Cross sectional or cross section).tw. (577910) 49 Cross-sectional studies/ (495873) 50 *"Surveys and Questionnaires"/ (50097) 51 *Registries/ or ((patient adj2 registr*) or (registr* adj2 (study or data))).ti,ab,kf. (62433) 52 39 or 40 or 41 or 42 or 43 or 44 or 45 or 46 or 47 or 48 or 49 or 50 or 51 (4307207) 53 38 or 52 (6768031) | Lines 5−37 is our search filter to limit to studies reporting randomised or controlled clinical trials. This filter is fundamentally the CADTH RCT/CCT filter.^8^  Researchers commonly amend established search filters to increase sensitivity: that is, to further decrease the risk of missing studies.^9,10^ We have done so here, as follows:  Line 25: we added ‘arm’ or ‘arms’ to aid identification of single-arm studies;  Line 28: we include nRCT;  Line 31: we added terminology for sub-groups, for instance where studies report an evaluation of a subgroup of interest;  Line 36: CADTH use an adapted version of the Cooper P3 filter. We use the full version here which we have amended to include truncation of phase.^10^ This covers identification of a study which reports as Phase 2 study or Phase 2b.  Line 37: we include a common misspelling of trial (i.e., trail).  Lines 39−51 is our search for studies using observational designs. Here, we use the Scottish Intercollegiate Guidelines Network (SIGN) search filter.^11^ We have amended this filter as follows:  Line 43: we focus only on the term cohort to ensure identification of cohort studies or where cohorts are followed by the term study is not used to define scope;  Line 50: we include indexing to identify surveys; and  Line 51: we include indexing and free-text to identify patient registry studies or data.  The search filters are combined using a Boolean OR connector. This means that all search filters are combined at this stage of the search. |
| 54 (2000* or 2001* or 2002* or 2003* or 2004* or 2005* or 2006* or 2007* or 2008* or 2009* or 2010* or 2011* or 2012* or 2013* or 2014* or 2015* or 2016* or 2017* or 2018* or 2019* or 2020* or 2021* or 2022* or 2023* or 2024*).dt,dp,ed,ep,yr. (25634510) 55 4 and 53 and 54 (2235) | Line 54 date limits the search to 2000–current.  Line 55 completes the search of MEDLINE by combining:  Line 4: condition terms  Line 53: search filters for RCT/CCT and observational studies  Line 54: date limit. |
| Abbreviations: CADTH, Canadian Agency for Drugs and Technologies in Health; CCT, controlled clinical trial; RCT, randomised clinical trial. | |

Supplementary Table 2. Search strategy for EMBASE (via Ovid), on 18 March 2024 (date limit 1974–15 March 2024)

| # | Searches | Results |
| --- | --- | --- |
| 1 | *osteogenesis imperfecta/ | 5082 |
| 2 | ((osteogenesis adj2 (imperfecta or fragil*)) or (imperfecta adj2 ossium) or (Brittle adj3 Bone*) or (fragile adj2 bone*) or (fragility adj2 bone*) or (glass adj2 bone*) or glassbone* or "soft bone*1" or bruck syndrome or osteopsathyrosis or "periostal aplasia" or (lobstein* adj2 (disease or syndrome*)) or ((imperfecta or fragil*) adj2 (ossium* or vitera))).ti,ab,kf. | 12341 |
| 3 | ((broken or break or breaking or fracture*1 or fracturing) adj5 (frequently or often or easily or easy or regular or regularly or constantly or continually or continuously routinely or repeatedly or "over and over" or always or recurrent) adj5 bone*).ti,ab,kf. | 1225 |
| 4 | 1 or 2 or 3 | 13586 |
| 5 | (Randomized Controlled Trial or Controlled Clinical Trial or Pragmatic Clinical Trial or Equivalence Trial or Clinical Trial, Phase III).pt. | 0 |
| 6 | Randomized Controlled Trial/ | 812696 |
| 7 | exp Randomized Controlled Trials as Topic/ | 270746 |
| 8 | "Randomized Controlled Trial (topic)"/ | 270620 |
| 9 | Controlled Clinical Trial/ | 472616 |
| 10 | exp Controlled Clinical Trials as Topic/ | 280140 |
| 11 | "Controlled Clinical Trial (topic)"/ | 13452 |
| 12 | Randomization/ | 99080 |
| 13 | Random Allocation/ | 93872 |
| 14 | Double-Blind Method/ | 192052 |
| 15 | Double Blind Procedure/ | 217019 |
| 16 | Double-Blind Studies/ | 174478 |
| 17 | Single-Blind Method/ | 51908 |
| 18 | Single Blind Procedure/ | 53974 |
| 19 | Single-Blind Studies/ | 53974 |
| 20 | Placebos/ | 353497 |
| 21 | Placebo/ | 410382 |
| 22 | Control Groups/ | 110758 |
| 23 | Control Group/ | 110758 |
| 24 | (random* or sham or placebo*).ti,ab,hw,kf,kw. | 2643499 |
| 25 | ((singl* or doubl*) adj (blind* or dumm* or mask* or arm or arms)).ti,ab,hw,kf,kw. | 402486 |
| 26 | ((tripl* or trebl*) adj (blind* or dumm* or mask*)).ti,ab,hw,kf,kw. | 2326 |
| 27 | (control* adj3 (study or studies or trial* or group*)).ti,ab,kf,kw. | 1788993 |
| 28 | (Nonrandom* or non random* or non-random* or nRCT or quasi-random* or quasirandom*).ti,ab,hw,kf,kw. | 73169 |
| 29 | allocated.ti,ab,hw. | 113696 |
| 30 | ((open label or open-label) adj5 (study or studies or trial* or extension)).ti,ab,hw,kf,kw. | 93456 |
| 31 | ((sub* and (group adj2 anal*)) or (subgroup adj2 anal*)).ti,ab,kw,kf. | 128892 |
| 32 | ((equivalence or superiority or non-inferiority or noninferiority) adj3 (study or studies or trial*)).ti,ab,hw,kf,kw. | 19521 |
| 33 | (pragmatic study or pragmatic studies).ti,ab,hw,kf,kw. | 993 |
| 34 | ((pragmatic or practical) adj3 trial*).ti,ab,hw,kf,kw. | 9308 |
| 35 | ((quasiexperimental or quasi-experimental) adj3 (study or studies or trial*)).ti,ab,hw,kf,kw. | 21082 |
| 36 | ("Phase 3*" or "phase3*" or "phase III*" or P3* or "PIII*" or "Phase 2*" or "phase2*" or "phase II*" or P2* or "PII*").ti,ab,kw,kf. | 635274 |
| 37 | (trial or trail).ti,ab,kw,kf. | 1209507 |
| 38 | 5 or 6 or 7 or 8 or 9 or 10 or 11 or 12 or 13 or 14 or 15 or 16 or 17 or 18 or 19 or 20 or 21 or 22 or 23 or 24 or 25 or 26 or 27 or 28 or 29 or 30 or 31 or 32 or 33 or 34 or 35 or 36 or 37 | 4762486 |
| 39 | Clinical study/ | 166079 |
| 40 | Case control study/ | 214643 |
| 41 | Family study/ | 25802 |
| 42 | Longitudinal study/ | 208874 |
| 43 | Retrospective study/ | 1586299 |
| 44 | Prospective study/ | 909571 |
| 45 | Randomized controlled trials/ | 270620 |
| 46 | 44 not 45 | 898504 |
| 47 | Cohort analysis/ | 1134324 |
| 48 | (Cohort adj (study or studies)).mp. | 506035 |
| 49 | (Case control adj (study or studies)).tw. | 174118 |
| 50 | (follow up adj (study or studies)).tw. | 75583 |
| 51 | (observational adj (study or studies)).tw. | 270954 |
| 52 | (epidemiologic$ adj (study or studies)).tw. | 124992 |
| 53 | (cross sectional adj (study or studies)).tw. | 365553 |
| 54 | or/39-43,46-53 | 4159840 |
| 55 | 38 or 54 | 7984455 |
| 56 | (2000* or 2001* or 2002* or 2003* or 2004* or 2005* or 2006* or 2007* or 2008* or 2009* or 2010* or 2011* or 2012* or 2013* or 2014* or 2015* or 2016* or 2017* or 2018* or 2019* or 2020* or 2021* or 2022* or 2023* or 2024*).yr. | 29957393 |
| 57 | 4 and 55 and 56 | 3048 |
| 58 | (conference abstract* or conference review or conference paper or conference proceeding).db,pt,su. | 5871958 |
| 59 | 57 not 58 | 2002 |
| 60 | limit 59 to embase | 1736 |

Supplementary Table 3. Search strategy for EMBASE conferences (via Ovid), on 18 March 2024 (date limit 1974–March 2024)

| # | Searches | Results |
| --- | --- | --- |
| 1 | *osteogenesis imperfecta/ | 5082 |
| 2 | ((osteogenesis adj2 (imperfecta or fragil*)) or (imperfecta adj2 ossium) or (Brittle adj3 Bone*) or (fragile adj2 bone*) or (fragility adj2 bone*) or (glass adj2 bone*) or glassbone* or "soft bone*1" or bruck syndrome or osteopsathyrosis or "periostal aplasia" or (lobstein* adj2 (disease or syndrome*)) or ((imperfecta or fragil*) adj2 (ossium* or vitera))).ti,ab,kf. | 12341 |
| 3 | ((broken or break or breaking or fracture*1 or fracturing) adj5 (frequently or often or easily or easy or regular or regularly or constantly or continually or continuously routinely or repeatedly or "over and over" or always or recurrent) adj5 bone*).ti,ab,kf. | 1225 |
| 4 | 1 or 2 or 3 | 13586 |
| 5 | (Randomized Controlled Trial or Controlled Clinical Trial or Pragmatic Clinical Trial or Equivalence Trial or Clinical Trial, Phase III).pt. | 0 |
| 6 | Randomized Controlled Trial/ | 812696 |
| 7 | exp Randomized Controlled Trials as Topic/ | 270746 |
| 8 | "Randomized Controlled Trial (topic)"/ | 270620 |
| 9 | Controlled Clinical Trial/ | 472616 |
| 10 | exp Controlled Clinical Trials as Topic/ | 280140 |
| 11 | "Controlled Clinical Trial (topic)"/ | 13452 |
| 12 | Randomization/ | 99080 |
| 13 | Random Allocation/ | 93872 |
| 14 | Double-Blind Method/ | 192052 |
| 15 | Double Blind Procedure/ | 217019 |
| 16 | Double-Blind Studies/ | 174478 |
| 17 | Single-Blind Method/ | 51908 |
| 18 | Single Blind Procedure/ | 53974 |
| 19 | Single-Blind Studies/ | 53974 |
| 20 | Placebos/ | 353497 |
| 21 | Placebo/ | 410382 |
| 22 | Control Groups/ | 110758 |
| 23 | Control Group/ | 110758 |
| 24 | (random* or sham or placebo*).ti,ab,hw,kf,kw. | 2643499 |
| 25 | ((singl* or doubl*) adj (blind* or dumm* or mask* or arm or arms)).ti,ab,hw,kf,kw. | 402486 |
| 26 | ((tripl* or trebl*) adj (blind* or dumm* or mask*)).ti,ab,hw,kf,kw. | 2326 |
| 27 | (control* adj3 (study or studies or trial* or group*)).ti,ab,kf,kw. | 1788993 |
| 28 | (Nonrandom* or non random* or non-random* or nRCT or quasi-random* or quasirandom*).ti,ab,hw,kf,kw. | 73169 |
| 29 | allocated.ti,ab,hw. | 113696 |
| 30 | ((open label or open-label) adj5 (study or studies or trial* or extension)).ti,ab,hw,kf,kw. | 93456 |
| 31 | ((sub* and (group adj2 anal*)) or (subgroup adj2 anal*)).ti,ab,kw,kf. | 128892 |
| 32 | ((equivalence or superiority or non-inferiority or noninferiority) adj3 (study or studies or trial*)).ti,ab,hw,kf,kw. | 19521 |
| 33 | (pragmatic study or pragmatic studies).ti,ab,hw,kf,kw. | 993 |
| 34 | ((pragmatic or practical) adj3 trial*).ti,ab,hw,kf,kw. | 9308 |
| 35 | ((quasiexperimental or quasi-experimental) adj3 (study or studies or trial*)).ti,ab,hw,kf,kw. | 21082 |
| 36 | ("Phase 3*" or "phase3*" or "phase III*" or P3* or "PIII*" or "Phase 2*" or "phase2*" or "phase II*" or P2* or "PII*").ti,ab,kw,kf. | 635274 |
| 37 | (trial or trail).ti,ab,kw,kf. | 1209507 |
| 38 | 5 or 6 or 7 or 8 or 9 or 10 or 11 or 12 or 13 or 14 or 15 or 16 or 17 or 18 or 19 or 20 or 21 or 22 or 23 or 24 or 25 or 26 or 27 or 28 or 29 or 30 or 31 or 32 or 33 or 34 or 35 or 36 or 37 | 4762486 |
| 39 | Clinical study/ | 166079 |
| 40 | Case control study/ | 214643 |
| 41 | Family study/ | 25802 |
| 42 | Longitudinal study/ | 208874 |
| 43 | Retrospective study/ | 1586299 |
| 44 | Prospective study/ | 909571 |
| 45 | Randomized controlled trials/ | 270620 |
| 46 | 44 not 45 | 898504 |
| 47 | Cohort analysis/ | 1134324 |
| 48 | (Cohort adj (study or studies)).mp. | 506035 |
| 49 | (Case control adj (study or studies)).tw. | 174118 |
| 50 | (follow up adj (study or studies)).tw. | 75583 |
| 51 | (observational adj (study or studies)).tw. | 270954 |
| 52 | (epidemiologic$ adj (study or studies)).tw. | 124992 |
| 53 | (cross sectional adj (study or studies)).tw. | 365553 |
| 54 | or/39-43,46-53 | 4159840 |
| 55 | 38 or 54 | 7984455 |
| 56 | (2022* or 2023* or 2024*).yr. | 4163809 |
| 57 | 4 and 55 and 56 | 563 |
| 58 | (conference abstract* or conference review or conference paper or conference proceeding).db,pt,su. | 5871958 |
| 59 | 57 and 58 | 150 |

Supplementary Table 4. Search strategy for CPCIS-S (via Clarivate), on 18 March 2024 (date limit 1990–current)

| ID | Search Hits | Results |
| --- | --- | --- |
| 1 | : ((osteogenesis NEAR/2 imperfecta) or (Brittle NEAR/3 Bone*)) (Topic) and 2024 or 2023 or 2022 (Publication Years) | 52 |
| Abbreviations: CPCIS-S, Conference Proceedings Citation Index – Science. | | |

Supplementary Table 5. Search strategy for Cochrane CENTRAL (via Wiley Interface), on 18 March 2024 (data parameter Issue 2 of 12, February 2024)

| Search Hits | Results |
| --- | --- |
| MeSH descriptor: [Osteogenesis Imperfecta] this term only | 74 |
| ((osteogenesis NEAR/2 (imperfecta)) or (Brittle NEAR/3 Bone*)):ti,ab,kw | 159 |
| ((broken or break or breaking or fracture* or fracturing) NEAR/5 (frequently or often or easily or easy or regular or regularly or constantly or continually or continuously routinely or repeatedly or "over and over" or always or recurrent) NEAR/5 bone*):ti,ab,kw | 57 |
| #1 or #2 or #3 | 211 |

Supplementary Table 6. Search strategy for APA PsycInfo (via OVID), on 18 March 2024 (date limit 1806 to Week 3 March 2024)

| # | Searches | Results |
| --- | --- | --- |
| 1 | ((osteogenesis adj2 (imperfecta or fragil*)) or (imperfecta adj2 ossium) or (Brittle adj3 Bone*) or (fragile adj2 bone*) or (fragility adj2 bone*) or (glass adj2 bone*) or glassbone* or "soft bone*1" or bruck syndrome or osteopsathyrosis or "periostal aplasia" or (lobstein* adj2 (disease or syndrome*)) or ((imperfecta or fragil*) adj2 (ossium* or vitera))).ti,ab. | 112 |
| 2 | ((broken or break or breaking or fracture*1 or fracturing) adj5 (frequently or often or easily or easy or regular or regularly or constantly or continually or continuously routinely or repeatedly or "over and over" or always or recurrent) adj5 bone*).ti,ab. | 13 |
| 3 | 1 or 2 | 122 |
| 4 | (2000* or 2001* or 2002* or 2003* or 2004* or 2005* or 2006* or 2007* or 2008* or 2009* or 2010* or 2011* or 2012* or 2013* or 2014* or 2015* or 2016* or 2017* or 2018* or 2019* or 2020* or 2021* or 2022* or 2023* or 2024*).yr. | 3707036 |
| 5 | 3 and 4 | 106 |

Supplementary Table 7. Search strategy for ClinicalTrials.gov^a^ on 18 March 2024

| # | Searches | Results |
| --- | --- | --- |
| 1 | ((osteogenesis imperfecta) OR (brittle AND bone) OR (brittle AND bones) OR (glassbone) OR (glassbones) OR (glass bone) OR (glass bones) OR (Brittle bone disease)) | 227 |
| Notes: ^a^ ClinialTrials.gov searched through: <https://classic.clinicaltrials.gov/ct2/results/refine?show_xprt=Y>. | | |

Supplementary Table 8. Search strategy for WHO ICTRP^a^ on 18 March 2024

| # | Searches | Results |
| --- | --- | --- |
| 1 | ((osteogenesis imperfecta) OR (brittle AND bone) OR (brittle AND bones) OR (glassbone) OR (glassbones) OR (glass bone) OR (glass bones) OR (Brittle bone disease)) | 170 |
| Notes. ^a^ WHO ICTRP searched through: <https://trialsearch.who.int/Default.aspx>. Abbreviations: ICTRP, Internation Clinical Trials Registry Platform; WHO, World Health Organization. | | |

Supplementary Table 9. Data extraction variables

| Information category |  | Variables |
| --- | --- | --- |
| **Primary extraction form** | | |
| General | | Authors, year of study, study title, study design, study objectives, country of study |
| Participant characteristics | | Age, gender |
| Outcomes^a^ | | Age of first fracture, fracture rate, healing time, downstream events/SSEs |
| **Secondary extraction form** | | |
| Participant characteristics | | OI type, risk factors, treatment |
| Outcome data / results^b^ | | Age of first fracture, fracture rate, healing time, downstream events/SSEs |
| Notes: ^a^ Outcomes were categorised as available/unavailable (yes/no) in the primary extraction sheet. ^b^ The secondary extraction sheet includes the actual numerical data, alongside measures of central tendencies. Abbreviations: OI, Osteogenesis imperfecta; SSEs, signs symptoms and events. | | |

Supplementary Table 10. Studies excluded at full text screening (n=296)

| **Reference** | **Reason for exclusion** |
| --- | --- |
| Ablon, J. (2003). "Personality and stereotype in osteogenesis imperfecta: behavioral phenotype or response to life's hard challenges?" Am J Med Genet Part A **122**(3): 201–214. | No outcomes of interest |
| Agarwal, V. and B. Joseph (2005). "Non-union in osteogenesis imperfecta." J Pediatr Orthop B **14**(6): 451–455. | Sample size |
| Ahn, J., et al. (2019). "Acetabular protrusio in patients with osteogenesis imperfecta: Risk Factors and Progression." J Pediatr Orthop **39**(10): e750–e754. | No outcomes of interest |
| Al-Agha, A. E., et al. (2016). "Pediatric cases of osteogenesis imperfecta and the effect of cyclic zoledronic acid therapy." Gazzetta Medica Italiana Archivio per le Scienze Mediche **175**(3): 85–96. | No outcomes of interest |
| Alhamad, A., et al. (2024). "Genotype and phenotype characteristics of osteogenesis imperfecta in Saudi Arabia." Eur J Hum Genet **32**: 146–146. | No outcomes of interest |
| Amako, M., et al. (2004). "Functional analysis of upper limb deformities in osteogenesis imperfecta." J Pediatr Orthop **24**(6): 689–694. | No outcomes of interest |
| Amarasena, S., et al. (2005). "Cyclical intravenous pamidronate therapy in children with osteogenesis imperfecta." Ceylon Med J **50**(3): 137–138. | Irrelevant publication type |
| Anam, E. A., et al. (2015). "Osteotomy healing in children with osteogenesis imperfecta Receiving Bisphosphonate Treatment." J Bone Miner Res **30**(8): 1362–1368. | No outcomes of interest |
| Andersen, J. D., et al. (2019). "Do femoral fractures in adult patients with osteogenesis imperfecta imitate atypical femoral fractures? A case series." Osteoporos Int **30**(2): 513–517. | Sample size |
| Anissipour, A. K., et al. (2014). "Behavior of scoliosis during growth in children with osteogenesis imperfecta." J Bone Joint Surg Am **96**(3): 237–243. | No outcomes of interest |
| Apolinario, A. C., et al. (2015). "Pamidronate affects the mandibular cortex of children with osteogenesis imperfecta." J Dent Res **94**(3 Suppl): 95S–102S. | No outcomes of interest |
| Apolinario, A. C., et al. (2016). "Dental panoramic indices and fractal dimension measurements in osteogenesis imperfecta children under pamidronate treatment." Dentomaxillofac Radiol **45**(4): 20150400. | No outcomes of interest |
| Arikoski, P. M. S., et al. (2004). "Intravenous pamidronate treatment in children with moderate to severe osteogenesis imperfecta: assessment of indices of dual-energy X-ray absorptiometry and bone metabolic markers during the first year of therapy." Bone **34**(3): 539–546. | No outcomes of interest |
| Artemis Doulgeraki, A. A., et al. (2022). "Effect of bisphosphonate treatment on oral health status of children and adolescents with osteogenesis imperfecta." OI Congress 2022. | No outcomes of interest |
| Ashby, E., et al. (2018). "Functional Outcome of Forearm Rodding in Children With Osteogenesis Imperfecta." J Pediatr Orthop **38**(1): 54–59. | No outcomes of interest |
| Ashby, E., et al. (2018). "Functional Outcome of Humeral Rodding in Children With Osteogenesis Imperfecta." J Pediatr Orthop **38**(1): 49–53. | No outcomes of interest |
| Ashford, R. U., et al. (2003). "Oral clodronate as treatment of osteogenesis imperfecta." Arch Dis Child **88**(10): 945. | Irrelevant publication type |
| Astrom, E., et al. (2010). "Biochemical bone markers in the assessment and pamidronate treatment of children and adolescents with osteogenesis imperfecta." Acta Paediatr **99**(12): 1834–1840. | Sample size |
| Astrom, E. and S. Soderhall (2002). "Beneficial effect of long-term intravenous bisphosphonate treatment of osteogenesis imperfecta." Arch Dis Child **86**(5): 356–364. | No outcomes of interest |
| Azevedo, C., et al. (2022). "HSD110 JAV-Raras: Comparative analysis of patient’s costs according to clinical and therapeutic protocol (PCDT) and current clinical practice for osteogenesis imperfecta (OI) in brazilian public healthcare system." Value Health **25**(7): S499. | No outcomes of interest |
| Azzam, K. A., et al. (2018). "mid-term results of femoral and tibial osteotomies and Fassier-Duval nailing in children with osteogenesis imperfecta." J Pediatr Orthop **38**(6): 331–336. | No outcomes of interest |
| Badin, D., et al. (2023). "Outcomes of Growth-friendly Instrumentation in Osteogenesis Imperfecta: A Preliminary Report." J Pediatr Orthop **43**(6): e458–e464. | No outcomes of interest |
| Bains, J., et al. (2017). "2016 Annual Meeting of the American Society for Bone and Mineral Research Atlanta, GA September 16-19, 2016." J Bone Miner Res **31**(S1): 1. | No outcomes of interest |
| Baldridge, D., et al. (2008). "CRTAP and LEPRE1 mutations in recessive osteogenesis imperfecta." Hum Mutat **29**(12): 1435–1442. | Sample size |
| Ball S, Roberts M, Saraff V, Sakka S, Shaw N, Hogler W. Is a modified version of the Childhood Health Assessment Questionnaire (CHAQ) a useful tool to identify the level of disability in children with osteogenesis imperfecta? Bone Abstracts. 2017;6. | No outcomes of interest |
| Banerjee, I. S., et al. (2002). "Osteogenesis imperfecta and intravenous pamidronate." Arch Dis Childhood **87**(6): 562–563. | No outcomes of interest |
| Becker, J., et al. (2011). "Exome Sequencing Identifies Truncating Mutations in Human SERPINF1 in Autosomal-Recessive Osteogenesis Imperfecta." Am J Hum Genet **88**(3): 362–371. | Sample size |
| Belyea, C. M. and J. B. Knox (2020). "Spinal fusion in children with osteogenesis imperfecta: A nationwide retrospective comparative cohort study over a 12-year period." Current Orthop Pract **31**(1): 72–75. | No outcomes of interest |
| Binh, H. D., et al. (2016). "The clinical features of osteogenesis imperfecta in Vietnam." Int Orthop **41**(1): 21–29. | No outcomes of interest |
| Bishop, N. (2010). "Characterising and treating osteogenesis imperfecta." Early Hum Dev **86**(11): 743–746. | Irrelevant publication type |
| Bishop, N. (2023). "Genetics and Therapy of Osteogenesis Imperfecta." Eur J Hum Genet **31**: 87–87. | No outcomes of interest |
| Bishop, N., et al. (2023). "11th International Meeting of Paediatric Endocrinology (IMPE) - Abstracts: Orbit: A randomized phase 2/3 study consisting of a phase 2 single-blind, dose-evaluation phase and a phase 3, double-blind, placebo-controlled phase to assess the efficacy and safety of setrusumab in subjects with osteogenesis imperfecta." Horm Res Paediatr **Vol. Suppl 2**(Supplement 2): 1–197. | No outcomes of interest |
| Bober M.B., et al. (2022). "Self-reported prevalence and impact of clinical signs, Symptoms, and events in adults with osteogenesis imperfecta (OI)." Value Health **25**(12). | No outcomes of interest |
| Boutaud, B. and J. M. Laville (2004). "[Elastic sliding central medullary nailing with osteogenesis imperfecta. Fourteen cases at eight years follow-up]." Rev Chir Orthop Reparatrice Appar Mot **90**(4): 304–311. | No outcomes of interest |
| Brizola, E., et al. (2017). " Características clínicas e padrão de fraturas no momento do diagnóstico de osteogênese imperfeita em crianças." Revista Paulista de Pediatria **35**(2): 171–177. | No outcomes of interest |
| Byrd J, White A, Schissel M, Ormer MV, Velasco D, Wallace M. FRI-514 Genotype-Phenotype Correlations in Osteogenesis Imperfecta: A Retrospective Study in 294 Patients. J Bone Miner Res 2023;36 (Suppl 1). | No outcomes of interest |
| Carlson, S. W., et al. (2020). "Total hip arthroplasty in patients with osteogenesis imperfecta." J Arthroplasty **35**(8): 2131–2135. | No outcomes of interest |
| Castro L, Santos C, Campos M, Gameleira K, Rorato M, Bezerra A, et al. Osteogenesis imperfecta—Clinical outcomes after a 4-year experience with cyclical intravenous pamidronate therapy. Bone. 2009;45:S59. | No outcomes of interest |
| Chagas, C. E., et al. (2012). "Do patients with osteogenesis imperfecta need individualized nutritional support?" Nutrition **28**(2): 138–142. | No outcomes of interest |
| Charpie, M., et al. (2024). "Genotype-phenotype correlation and effects of bisphosphonates in rare forms of osteogenesis imperfecta: a retrospective study." Eur J Hum Genet **32**: 143–144. | No outcomes of interest |
| Chen, P., et al. (2022). "Phenotypic spectrum and molecular basis in a Chinese cohort of osteogenesis imperfecta with mutations in Type I collagen." Front Genet **13**: 816078. | No outcomes of interest |
| Chen, P., et al. (2023). "Scoliosis in osteogenesis imperfecta: identifying the genetic and non-genetic factors affecting severity and progression from longitudinal data of 290 patients." Orphanet J Rare Dis **18**(1): 295. | No outcomes of interest |
| Cheung, M., et al. (2023). "Abstracts of the ESPE 61 Annual Society Meeting: Short term side effects of first bisphosphonate infusion in children with different underlying bone pathologies." Horm Res Paediatr **96**(Supplement 4): 1–643. | No outcomes of interest |
| Cheung, M. S., et al. (2007). "Natural history of hyperplastic callus formation in osteogenesis imperfecta type V." J Bone Miner Res **22**(8): 1181–1186. | No outcomes of interest |
| Cheung, M. S., et al. (2009). "Intravenous pamidronate in osteogenesis imperfecta type VII." Calcif Tissue Int **84**(3): 203–209. | Population not of interest |
| Cheung, M. S. A., et al. (2010). "Cranial base abnormalities in osteogenesis imperfecta: phenotypic and genotypic determinants." J Bone Miner Res **26**(2): 405–413. | Population not of interest |
| Chhabra, B. N., et al. (2023). "Pediatric hand and wrist fractures in osteogenesis imperfecta: an analysis of incidence, patient-specific risk factors, and fracture-specific characteristics." J Pediatr Orthop **43**(8): e680–e685. | No outcomes of interest |
| Chiarello, E., et al. (2012). "Conservative versus surgical treatment of osteogenesis imperfecta: a retrospective analysis of 29 patients." Clin Cases Miner Bone Metab **9**(3): 191–194. | Population not of interest |
| Cho, T. J., et al. (2015). "Locking plate placement with unicortical screw fixation adjunctive to intramedullary rodding in long bones of patients with osteogenesis imperfecta." J Bone Joint Surg Am **97**(9): 733–737. | No outcomes of interest |
| Coussens, M., et al. (2022). "Bone mass, density, geometry, and stress-strain index in adults with osteogenesis imperfecta Type I and their associations with physical activity and muscle function parameters." J Bone Miner Res **37**(12): 2456–2465. | No outcomes of interest |
| Cox, I., et al. (2020). "Combined two-centre experience of single-entry telescopic rods identifies characteristic modes of failure." Bone Joint J **102-B**(8): 1048–1055. | No outcomes of interest |
| Cronjé, L., et al. (2020). "A retrospective study to evaluate the anaesthetic choices and complications for patients with osteogenesis imperfecta at a quaternary referral hospital." South Afr J of Anaesth Analg **26**(1): 45–50. | No outcomes of interest |
| Dar S, Khalily N, Khan S, Kamwa V, Cole T, Ayuk J, et al., editors. Management of osteogenesis imperfecta in adulthood-a single centre experience. Endocrine Abstracts; 2018: Bioscientifica. | No outcomes of interest |
| Darba, J. and A. Marsa (2020). "Hospital incidence, management and direct cost of osteogenesis imperfecta in Spain: a retrospective database analysis." J Med Econ **23**(12): 1435–1440. | No outcomes of interest |
| Das, R. R. (2011). "Early treatment of osteogenesis imperfecta: which drug to use?" Early Hum Dev **87**(12): 805. | Irrelevant publication type |
| Das, R. R. (2011). "Hypocalcemia associated with zoledronic acid for osteogenesis imperfecta." Horm Res Paediatr **76**(4): 291; author reply 292. | Irrelevant publication type |
| Dashdamirova, S. and D. Gogas Yavuz (2022). "World Congress on Osteoporosis, Osteoarthritis and Musculoskeletal Diseases (WCO-IOF-ESCEO 2021): Poster Abstracts: Ten cases of osteogenesis imperfecta: Clinical features, outcomes and treatment response." Osteoporos Int **32**(S1): 119–410. | No outcomes of interest |
| de Graaff, F., et al. (2011). "Decrease in outpatient department visits and operative interventions due to bisphosphonates in children with osteogenesis imperfecta." J Child Orthop **5**(2): 121–125. | No outcomes of interest |
| Diaz Lopez, M., et al. (2020). "Osteogenesis imperfecta. Report of 15 Cases." Rheumatol Clin (Engl Ed) **16**(2 Pt 2): 165–168. | No outcomes of interest |
| Dimeglio, L. A., et al. (2005). "A comparison of oral and intravenous bisphosphonate therapy for children with osteogenesis imperfecta." J Pediatr Endocrinol Metab **18**(1): 43–53. | No outcomes of interest |
| Edmonds T, D. O., et al. (2024). "Estimating the Benefits of Reduced Fracture Incidence for Patients Living with Osteogenesis Imperfecta and for Healthcare Payers." Value Health **27**(6): S1. | No outcomes of interest |
| Edmonds T, D. O., Mumford A, Henriksen O, Waker M, Whitcher C, Pinedo-Villanueva R (2024). "Estimating the burden of osteogenesis imperfecta on patients and healthcare payers: A health economic modelling study." Value Health **27**(6): S1. | No outcomes of interest |
| El-Adl, G., et al. (2009). "Telescoping versus non-telescoping rods in the treatment of osteogenesis imperfecta." Acta Orthop Belg **75**(2): 200–208. | No outcomes of interest |
| Ella Riley, et al. (2022). "What's in a name? Prevalence of metaphyseal fractures in children with osteogenesis imperfecta in the first two years of life." International Conference on Children’s Bone Health 2022. | No outcomes of interest |
| Elżbieta Jakubowska-Pietkiewicz, J. N., et al. (2022). "Assessment of health status of newborns with severe osteogenesis imperfecta – 21 years of observations." International Conference on Children’s Bone Health 2022. | No outcomes of interest |
| Engel Espinosa, W., et al. (2011). "[Anesthetic treatment of patients with osteogenesis imperfecta]." Rev Esp Anestesiol Reanim **58**(3): 151–155. | Not in English |
| Engelbert, R. H. H. U., et al. (2000). "Osteogenesis imperfecta in childhood: Prognosis for walking." J Pediatr **137**(3): 397–402. | No outcomes of interest |
| Escribano-Rey, R. J., et al. (2014). "[Osteogenesis imperfecta: Treatment and results of a case series]." Rev Esp Cir Ortop Traumatol **58**(2): 114–119. | Not in English |
| Essawi, O. H., et al. (2020). "New insights on the clinical variability of FKBP10 mutations." Eur J Med Genet **63**(9): 103980. | No outcomes of interest |
| Fano, V., et al. (2010). "[Osteogenesis imperfecta. Clinical, functional and multidisciplinary evaluation of 65 patients]." An Pediatr (Barc) **72**(5): 324–330. | No outcomes of interest |
| Fassier, F., et al. (2008). "Results and complications of a surgical technique for correction of coxa vara in children with osteopenic bones." J Pediatr Orthop **28**(8): 799–805. | No outcomes of interest |
| Fhima, F., et al. (2023). "Role of bisphosphonates in the management of osteogenesis imperfecta." Ann Rheum Dis **82**(Supplement 1): 1993. | No outcomes of interest |
| Fiscaletti, M., et al. (2018). "Diagnosis of recurrent fracture in a pediatric cohort." Calcif Tissue Int **103**(5): 529–539. | No outcomes of interest |
| Folkestad, L. (2018). "Mortality and morbidity in patients with osteogenesis imperfecta in Denmark." Dan Med J **65**(4). | Irrelevant publication type |
| Folkestad, L., et al. (2016). "Mortality and causes of death in patients with osteogenesis imperfecta: A register-based nationwide cohort study." J Bone Miner Res **31**(12): 2159–2166. | No outcomes of interest |
| Frank Rauch, M. B. B., et al. (2022). "The IMPACT survey has produced a self-reported, 2,276-respondent dataset of individuals with OI, their caregivers and close relatives." OI Congress 2022. | No outcomes of interest |
| Franzone, J. M., et al. (2020). "Evaluation of fracture and osteotomy union in the setting of osteogenesis imperfecta: Reliability of the Modified Radiographic Union Score for Tibial Fractures (RUST)." J Pediatr Orthop **40**(1): 48–52. | Population not of interest |
| Franzone, J. M. and R. W. Kruse (2018). "Intramedullary nailing with supplemental plate and screw fixation of long bones of patients with osteogenesis imperfecta: operative technique and preliminary results." J Pediatr Orthop B **27**(4): 344–349. | Population not of interest |
| Franzone, J. M., et al. (2022). "Multicenter Series of Deformity Correction Using Guided Growth in the Setting of Osteogenesis Imperfecta." J Pediatr Orthop **42**(6): e656–e660. | No outcomes of interest |
| Galhardo Saraiva, F., et al. (2024). "An analysis from a tertiary pediatric hospital: Does physical activity play a role in the management of children and young adults with osteogenesis imperfecta?" Cureus **16**(2): e53646. | No outcomes of interest |
| Galloway P, Nixon A, Rayner L, Panchbhaya N, Collins H, Skae M, et al. Assessment of multidisciplinary care of children with osteogenesis imperfecta at The Royal Manchester Children. Bone Abstracts. 2019;7. | No outcomes of interest |
| Gargano L, M. G., et al. (2022). "JAV-Raras: Assessing and communicating value of care with patient-centered outcomes and time-driven activity-based costing method: Analysis of osteogenesis imperfecta in Brazilian public healthcare system." Value Health **25**(7): S558. | No outcomes of interest |
| Garganta, M. D., et al. (2018). "Cyclic bisphosphonate therapy reduces pain and improves physical functioning in children with osteogenesis imperfecta." BMC Musculoskelet Disord **19**(1): 344. | Irrelevant publication type |
| Gaume, M., et al. (2022). "Tibial sliding elastic nailing technique in moderate-to-severe osteogenesis imperfecta: Long-term outcomes." J Pediatr Orthop **42**(1): 47–52. | No outcomes of interest |
| Gaume, M., et al. (2023). "Bone allografting: an original method for biological osteosynthesis and bone reinforcement in children with osteogenesis imperfecta." Int Orthop **47**(7): 1863–1869. | No outcomes of interest |
| George, S., et al. (2015). "Short-term safety of zoledronic acid in young patients with bone disorders: An extensive institutional experience." J Clin Endocrinol Metab **100**(11): 4163–4171. | Population not of interest |
| Georgescu, I., et al. (2014). "Burnei's technique of femoral neck variation and valgisation by using the intramedullary rod in osteogenesis imperfecta." J Med Life **7**(4): 493–498. | No outcomes of interest |
| Georgina Williams, J. A., et al. (2022). "Skull fractures, cervical spine fractures and intracranial injuries in children with osteogenesis imperfecta: a cohort study." OI Congress 2022. | No outcomes of interest |
| Gimeno-Martos, S., et al. (2017). "[Epidemiology of imperfect osteogenesis: a rare Disease in the Valencia Region.]." Rev Esp Salud Publica **91**. | Not in English |
| Glorieux, F. H., et al. (2004). "Alendronate in the treatment of pediatric osteogenesis imperfecta." J Bone Min Res **20 Suppl**: S12. | No outcomes of interest |
| Goeller, J. K. E., et al. (2017). "Perioperative management of pediatric patients with osteogenesis imperfecta undergoing orthopedic procedures." Curr Anesthesiol Rep **7**(2): 142–149. | No outcomes of interest |
| Goiano, E. O., et al. (2023). "Treatment of osteogenesis imperfecta using the fassier-duval telescopic rod." Acta Ortop Bras **31**(spe3): e266775. | No outcomes of interest |
| Gottesman G, Semler O, Felix TM, Hui Wang. SAT-500 Cosmic: An open-label, randomized, active-controlled, phase 3 study of setrusumab compared with bisphosphonates in pediatric subjects with osteogenesis imperfecta. J Bone Miner Res. 2023;38(Suppl 2). | No outcomes of interest |
| Graff, K., et al. (2020). "Spatio-temporal parameters and body of gait and body deformations in patients with osteogenesis imperfecta." Gait & Posture **81**(NA): 113–114. | No outcomes of interest |
| Greeley, C. S., et al. (2013). "Fractures at diagnosis in infants and children with osteogenesis imperfecta." J Pediatr Orthop **33**(1): 32–36. | No outcomes of interest |
| Grissom, L. E. and H. T. Harcke (2003). "Radiographic features of bisphosphonate therapy in pediatric patients." Pediatr Radiol **33**(4): 226–229. | No outcomes of interest |
| Grossman, L. S., et al. (2018). "Initial experience with percutaneous im rodding of the humeri in children with osteogenesis imperfecta." J Pediatr Orthop **38**(9): 484–489. | No outcomes of interest |
| Gurel, R., et al. (2022). "Cementless posterior spinal fusion for the treatment of OI patients with severe spine deformity-a case series." Eur Spine J **31**(6): 1566–1572. | No outcomes of interest |
| Hadef, D., et al. (2023). "E57 Osteogenesis imperfecta: about a series of twelve cases in Batna (Algeria)." Rheumatology **62**(Supplement_3): iii26. | No outcomes of interest |
| Hagberg M, Lowing K, Astrom E. Perceived activity capability in children and adolescents with osteogenesis imperfecta. Bone Abstracts. 2013;1. | No outcomes of interest |
| Hagberg M, Lowing K, Malmgren B, Kumlien C, Eva A. Team management of young persons with osteogenesis imperfecta. Bone Abstracts. 2014;3. | No outcomes of interest |
| Hald, J. D., et al. (2017). "Health-related quality of life in adults with osteogenesis imperfecta." Calcif Tissue Int **101**(5): 473–478. | No outcomes of interest |
| Hijazi, M., et al. (2021). "Stapes surgery in osteogenesis imperfecta: retrospective analysis of 18 operated ears." Eur Arch Otorhinolaryngol **278**(12): 4697–4705. | No outcomes of interest |
| Hogler, W., et al. (2017). "The effect of whole body vibration training on bone and muscle function in children with osteogenesis imperfecta." J Clin Endocrinol Metab **102**(8): 2734–2743. | No outcomes of interest |
| Holmes, K., et al. (2020). "Fassier-Duval rod failure: Is it related to positioning in the distal epiphysis?" J Pediatr Orthop **40**(8): 448–452. | No outcomes of interest |
| Hosseini, P., et al. (2023). "Evaluation of Factors Affecting Morbidity in Patients with Osteogenesis Imperfecta." J Acad Res Med **13**(1): 9–15. | No outcomes of interest |
| Hoyer-Kuhn, H., et al. (2014). "A specialized rehabilitation approach improves mobility in children with osteogenesis imperfecta." J Musculoskelet Neuronal Interact **14**(4): 445–453. | No outcomes of interest |
| Hoyer-Kuhn, H., et al. (2017). "Correlation of bone mineral density on quality of life in patients with osteogenesis imperfecta during treatment with denosumab." Pediatr Endocrinol Rev **15**(Suppl 1): 123–129. | No outcomes of interest |
| Huang, S., et al. (2023). "EPH138 osteogenesis imperfecta: Epidemiology characteristics and disease burden in two real-world databases." Value Health **26**(6): S189. | No outcomes of interest |
| Hupin E, Edwards K, Chueng M, Allgrove J, DeVile C. Do children with mild to moderate osteogenesis imperfecta (OI) with abdominal muscle weakness have a higher incidence of pars defects? A physiotherapy pilot. Bone Abstracts. 2015;4. | No outcomes of interest |
| Ibrahim, A. G. and H. A. Crockard (2007). "Basilar impression and osteogenesis imperfecta: a 21-year retrospective review of outcomes in 20 patients." J Neurosurg Spine **7**(6): 594–600. | No outcomes of interest |
| Imajima, Y., Motohiro, T.U. (2015). "Intramedullary fixation using Kirschner wires in children with osteogenesis imperfecta." J Pediatr Orthop **35**(4): 431–434. | No outcomes of interest |
| India Cox, L. A.-M., et al. (2022). "Characteristic modes of failure in single entry telescopic rods: a combined 2 centre experience." OI Congress 2022. | No outcomes of interest |
| Ito, M., et al. (2021). "Surgical outcome of spinal fusion for osteogenesis imperfecta with scoliosis: Is the hybrid system with pedicle screws applicable to weak, tiny, and fragile vertebrae?" J Pediatr Orthop **41**(6): 368–373. | No outcomes of interest |
| Janus, G. J., et al. (2000). "Operative treatment of severe scoliosis in osteogenesis imperfecta: results of 20 patients after halo traction and posterior spondylodesis with instrumentation." Eur Spine J **9**(6): 486–491. | No outcomes of interest |
| Javaid MK, Langdahl B, Chapurlat R, De Beur SJ, Sutton VR, Poole K, et al. Changes in BMD and Bone Turnover Markers Following Discontinuation of Setrusumab After 1 Year of Treatment in Adults with Osteogenesis Imperfecta. J Bone Miner Res. 2023;38(Supp 2):39-. | No outcomes of interest |
| Javaid, M. K., et al. (2022). "Baseline patient demographics and disease characteristics in adults with osteogenesis imperfecta (OI) in the Phase 2b ASTEROID Study." J Bone Min Res **37**: 163–163. | No outcomes of interest |
| Kashii, M., et al. (2019). "Development of scoliosis in young children with osteogenesis imperfecta undergoing intravenous bisphosphonate therapy." J Bone Miner Metab **37**(3): 545–553. | No outcomes of interest |
| Keemink Y, Sakkers R, Pruijs H. Zoledronate as first line therapy for pediatric osteogenesis imperfecta? Bone Abstracts. 2015;4. | No outcomes of interest |
| Khan, A. R., et al. (2022). "Causes and treatment of birth trauma-related femoral fracture: a longitudinal study." Pak J Med Health Sci **16**(8): 642–643. | Population not of interest |
| Kiranjit Joshi, R. P., Aris Siafarikas (2022). "Natural history of fractures in children and adolescents with osteogenesis imperfecta type 1: A study from Western Australia." OI Congress 2022. | No outcomes of interest |
| Kishta, W., et al. (2017). "Hip dysplasia in children with osteogenesis imperfecta: association with collagen Type I C-propeptide mutations." J Pediatr Orthop **37**(7): 479–483. | Sample size |
| Kocijan, R., et al. (2014). "Serum sclerostin levels are decreased in adult patients with different types of osteogenesis imperfecta." J Clin Endocrinol Metab **99**(2): E311–319. | No outcomes of interest |
| Kokavec, M., et al. (2008). "[Role of an interdisciplinary approach in the healing of long bone fractures in patients with osteogenesis imperfecta]." Acta Chir Orthop Traumatol Cech **75**(3): 185–189. | Not in English |
| Kolovos S, Javaid MK, Pinedo-Villanueva R. Hospital admissions of patients with osteogenesis imperfecta in the English NHS. Osteoporos Int 2021;32(6):1207-16. | No outcomes of interest |
| Kruger, K. M., et al. (2019). "Mobility in osteogenesis imperfecta: a multicenter North American study." Genet Med **21**(10): 2311–2318. | No outcomes of interest |
| Kuurila, K., et al. (2000). "Hearing loss in children with osteogenesis imperfecta." Eur J Paediatr **159**(7): 515–519. | No outcomes of interest |
| Labuda, M. M., et al. (2002). "Osteogenesis imperfecta type VII maps to the short arm of chromosome 3." Bone **31**(1): 19–25. | No outcomes of interest |
| Land, C., et al. (2006). "Cyclical intravenous pamidronate treatment affects metaphyseal modeling in growing patients with osteogenesis imperfecta." J Bone Miner Res **21**(3): 374–379. | No outcomes of interest |
| Land, C., et al. (2006). "Effect of intravenous pamidronate therapy on functional abilities and level of ambulation in children with osteogenesis imperfecta." J Pediatr **148**(4): 456–460. | No outcomes of interest |
| Land, C., et al. (2006). "Vertebral morphometry in children and adolescents with osteogenesis imperfecta: effect of intravenous pamidronate treatment." Bone **39**(4): 901–906. | No outcomes of interest |
| Leali, P. T., et al. (2017). "Efficacy of teriparatide vs neridronate in adults with osteogenesis imperfecta type I: a prospective randomized international clinical study." Clin Cases Miner Bone Metab **14**(2): 153–156. | No outcomes of interest |
| Lee, K., et al. (2015). "Proximal migration of femoral telescopic rod in children with osteogenesis imperfecta." J Pediatr Orthop **35**(2): 178–184. | Sample size |
| Lee, R. J., et al. (2016). "Bent telescopic rods in patients with osteogenesis imperfecta." J Pediatr Orthop **36**(6): 656–660. | No outcomes of interest |
| Lee, Y. S., et al. (2001). "Cyclic pamidronate infusion improves bone mineralisation and reduces fracture incidence in osteogenesis imperfecta." Eur J Pediatr **160**(11): 641–644. | Sample size |
| Lewiecki EM, Wang H, Gottesman G. LB SAT-650 Evaluating Setrusumab for the Treatment of Osteogenesis Imperfecta: Phase 2 Data from the Phase 2/3 ORBIT Study. J Bone Miner Res. 2023;38(Suppl 2). | No outcomes of interest |
| Li, W. C., et al. (2015). "Femoral non-elongating rodding in osteogenesis imperfecta - the importance of purchasing epiphyseal plate." Biomed J **38**(2): 143–147. | Sample size |
| Li, Y. H., et al. (2000). "The Sofield-Millar operation in osteogenesis imperfecta. A modified technique." J Bone Joint Surg Br **82**(1): 11–16. | No outcomes of interest |
| Lim, A., et al. (2020). "Outcomes of zoledronic acid use in paediatric conditions." Horm Res Paediatr **93**(7-8): 442–452. | Population not of interest |
| Lindgren, P., et al. (2023). "Poster abstracts of the ISPD 26th International Conference on Prenatal Diagnosis and Therapy, Montréal, Canada, 20–22 June 2022." Prenatal Diagnosis **43**(S1): 19–119. | No outcomes of interest |
| Letocha AD, Cintas HL, Troendle JF, et al. Controlled trial of pamidronate in children with types III and IV osteogenesis imperfecta confirms vertebral gains but not short-term functional improvement. *J Bone Miner Res:* 2005; 20: 977-986. | Duplicate |
| Low SA, Nielsen J, Lu X, Shen A, Chen C, Neidraur M. Fracture-Targeted Anabolic Therapy of Osteogenesis Imperfecta. J Bone Min Res; 2022. | No outcomes of interest |
| Lowing, K., et al. (2007). "Effect of intravenous pamidronate therapy on everyday activities in children with osteogenesis imperfecta." Acta Paediatr **96**(8): 1180–1183. | No outcomes of interest |
| Lv, F., et al. (2018). "Zoledronic acid versus alendronate in the treatment of children with osteogenesis imperfecta: A 2-year clinical study." Endocr Pract **24**(2): 179–188. | No outcomes of interest |
| Madhuri, V., et al. (2023). "An evaluation of safety and efficacy of multiple intravenous and intraosseous injections of fetal liver-derived MSCs in children with Osteogenesis Imperfecta (BOOST to BRITTLE BONES) - An exploratory open-label phase I/ II clinical trial." J Bone Miner Res **38**: 325–325. | Sample size |
| Maelle Charpie, P. B., et al. (2022). "Genotype-phenotype correlation and effects of bisphosphonates in rare forms of osteogenesis imperfecta: a retrospective study." OI Congress 2022. | No outcomes of interest |
| Malina, S. N., et al. (2023). "Access to care among patients with osteogenesis imperfecta during the COVID-19 pandemic." Arch Osteoporos **18**(1): 143. | No outcomes of interest |
| Gaume M, Duprot E, De Tienda M, Finidori G, Zagorka P, Langlais T. P39 Tibia sliding elastic nailing technique in moderate-to-severe osteogenesis imperfecta: long-term outcomes. OI Congress 2022; 30 August to 2 September 2022; Sheffield, UK. 2022. | No outcomes of interest |
| McAllion, S. J. P., Colin R. (2002). "Musculo-skeletal problems associated with pregnancy in women with osteogenesis imperfecta." J Obstet Gynaecol **22**(2): 169–172. | No outcomes of interest |
| McKiernan FE. Musculoskeletal manifestations of mild osteogenesis imperfecta in the adult. Osteoporos Int. 2005;16(12):1698-702. | No outcomes of interest |
| Mercier A, Gineyts E, Millet M, Borel O, Sornay-Rendu E, Auroux M, et al., P20 Variation in circulating levels of periostin in osteogenesis imperfecta. World Conference on Osteoporosis, Osteoarthritis and Musculoskeletal Disease; 2024; London, United Kingdom. | No outcomes of interest |
| Michalus, I., et al. (2019). "Pamidronate administration may result in anaemia in children with osteogenesis imperfecta." Arch Dis Child **104**(9): 906–907. | No outcomes of interest |
| Michalus I, Nowicka Z, Pietras W, Nowicka M, Byrwa A, Albińska P, et al. Anemia-novel clinically significant finding during intravenous pamidronate therapy of children diagnosed with osteogenesis imperfecta. Bone Abstracts. 2019;7. | No outcomes of interest |
| Mills L, Clark R, Birch L, Burren CP. Dietary behaviours and compromised nutritional intakes in children with Osteogenesis Imperfecta. Bone Abstracts. 2019;7. | No outcomes of interest |
| Mirko Rehberg, S. M., et al. (2022). "Treatment response in osteogenesis imperfecta - Two-year retrospective analysis of paediatric patients treated with bisphosphonates." OI Congress 2022. | No outcomes of interest |
| Mocritcaia, A., et al. (2024). "Structural heart disease in patients with osteogenesis imperfecta." International Osteoperosis Foundation. | No outcomes of interest |
| Mohd Nawawi, N., et al. (2018). "Genotype-phenotype correlation among Malaysian patients with osteogenesis imperfecta." Clin Chim Acta **484**: 141–147. | No outcomes of interest |
| Mohsenzade, P., et al. (2021). "Bone density, fractures and the associated factors in iranian children and adolescent with Osteogenesis Imperfecta." BMC Pediatr **21**(1): 37. | No outcomes of interest |
| Montpetit, K., et al. (2015). "Multidisciplinary treatment of severe osteogenesis imperfecta: Functional outcomes at skeletal maturity." Arch Phys Med Rehabil **96**(10): 1834–1839. | No outcomes of interest |
| Moreira, C. L. M., et al. (2015). "Physiotherapy and patients with osteogenesis imperfecta: an experience report." Fisioterapia em Movimento **28**(2): 307–317. | No outcomes of interest |
| Moshkovich, O. B., et al. (2017). "Development of The OIQoL-A: A health-related quality of life measure for adults with osteogenesis imperfecta." Value Health **20**(9): A538–NA. | No outcomes of interest |
| Munns, C. F., et al. (2005). "Effects of intravenous pamidronate treatment in infants with osteogenesis imperfecta: clinical and histomorphometric outcome." J Bone Miner Res **20**(7): 1235–1243. | No outcomes of interest |
| Murphy A, Howard A, Sochett E, Harrington J. Financial burden in families of children with osteogenesis imperfecta (OI). Bone Abstracts. 2017;6. | No outcomes of interest |
| Musielak, B. J., et al. (2021). "Problems, complications, and factors predisposing to failure of Fassier-Duval rodding in children with osteogenesis imperfecta: A double-center study." J Pediatr Orthop **41**(4): e347–e352. | No outcomes of interest |
| Nagamani, S., et al. (2013). "Teriparatide, the first anabolic agent for treatment of osteogenesis imperfecta improves bone mineral density at the hip and spine: a randomized, blinded, placebo-controlled trial." Proceedings of the 63rd Annual Meeting of the American Society of Human Genetics; 2013 Oct 22-26; Boston, USA.: 27. | No outcomes of interest |
| Nalini M.S., et al. (2022). "Phenotypic and genotype spectrum in a cohort of patients with Osteogenesis Imperfecta." International Conference on Children’s Bone Health 2022. | No outcomes of interest |
| Narayanan, V. K. D., et al. (2012). "A survey of current care for children and adults with osteogenesis imperfecta in Glasgow." Arch Dis Childhood **97**(Suppl 1): A102.103–A103. | No outcomes of interest |
| Ndour, O., et al. (2011). "Osteogenesis imperfecta in a pediatric surgical center in dakar, senegal: clinical and radiological aspects." J West Afr Coll Surg **1**(3): 1–14. | No outcomes of interest |
| Nguyen, T. H., et al. (2017). "AB107. Study of functional independence of patients with osteogenesis imperfecta." Ann Transl Med **5**(S2): AB107–AB107. | No outcomes of interest |
| Nicholas Bishop, E. R., et al. (2022). "Orbit: a randomized, double-blind, placebo-controlled, Phase 2/3 study to assess the efficacy and safety of setrusumab in pediatric and young adult participants with osteogenesis imperfecta." OI Congress 2022. | No outcomes of interest |
| Nicholas Bohannon, E. V. D. Z., et al. (2022). "Effects of neuraxial anesthesia on postoperative pain scores in children with osteogenesis imperfecta." OI Congress 2022. | No outcomes of interest |
| Nicolaou, N., et al. (2012). "Changing pattern of femoral fractures in osteogenesis imperfecta with prolonged use of bisphosphonates." J Child Orthop **6**(1): 21–27. | No outcomes of interest |
| Nicolaou, N., et al. (2011). "Use of the Sheffield telescopic intramedullary rod system for the management of osteogenesis imperfecta: clinical outcomes at an average follow-up of nineteen years." J Bone Joint Surg Am **93**(21): 1994–2000. | No outcomes of interest |
| Ohata, Y., et al. (2023). "Association of trabecular bone score and bone mineral apparent density with the severity of bone fragility in children and adolescents with osteogenesis imperfecta: A cross-sectional study." PLoS One **18**(8): e0290812. | No outcomes of interest |
| Oheim, R., et al. (2022). "Genetic diagnostics in routine osteological assessment of adult low bone mass disorders." J Clin Endocrinol Metab **107**(7): e3048–e3057. | No outcomes of interest |
| Okawa, R., et al. (2017). "Oral manifestations of Japanese patients with osteogenesis imperfecta." Pediatr Dental J **27**(2): 73–78. | No outcomes of interest |
| Onay, T., et al. (2023). "Long-term functional consequences of surgically treated pediatric and adolescent olecranon fractures: Does the presence of associated fractures and surgical procedure affect the results?" J Pediatr Orthop **43**(3): 135–142. | Population not of interest |
| Orlando, G., et al. (2021). "Physical function in UK adults with osteogenesis imperfecta: a cross-sectional analysis of the RUDY study." Osteoporos Int **32**(1): 157–164. | No outcomes of interest |
| Orwoll, E. S., et al. (2014). "Evaluation of teriparatide treatment in adults with osteogenesis imperfecta." J Clin Invest **124**(2): 491–498. | No outcomes of interest |
| O'Sullivan, E. S., et al. (2014). "Osteogenesis imperfecta in adults: phenotypic characteristics and response to treatment in an Irish cohort." Ir J Med Sci **183**(2): 225–230. | Sample size |
| Otaify, G. A., et al. (2016). "Zoledronic acid in children with osteogenesis imperfecta and Bruck syndrome: a 2-year prospective observational study." Osteoporos Int **27**(1): 81–92. | No outcomes of interest |
| Ozan Ali Erdal, B. G., et al. (2022). "Results of telescopic nailing in children with osteogenesis imperfecta after minimum five-year follow-up." OI Congress 2022. | No outcomes of interest |
| Ozturk, H., et al. (2008). "Extreme complications of Fixion nail in treatment of long bone fractures." Arch Orthop Trauma Surg **128**(3): 301–306. | Sample size |
| Pai, P. and K. Armon (2014). "Safe tolerance rate of disodium pamidronate infusion in children." Rheumatology **53**: iii16–iii17. | No outcomes of interest |
| Panigrahi, I., et al. (2009). "Zolendronate in osteogenesis imperfecta." J Pediatr Endocrinol Metab **22**(8): 763. | Irrelevant publication type |
| Panigrahi, I., et al. (2010). "Response to zolendronic acid in children with type III osteogenesis imperfecta." J Bone Min Res **28**(4): 451–455. | Irrelevant publication type |
| Pargas, C., et al. (2020). "Cervical kyphosis: A predominant feature of patients with osteogenesis imperfecta type 5." Bone Rep **13**: 100735. | No outcomes of interest |
| Payet, J. C., C. (2013). "FRI0375 Clinical characteristics and vitamin D insufficiency in a population of 54 adults with osteogenesis imperfecta." Ann Rheum Dis **71**(Suppl 3): 441–441. | No outcomes of interest |
| Peikai Chen, et al. (2022). "A retrospective review of 496 orthopedic surgeries in 223 Chinese patients of osteogenesis imperfecta." OI Congress 2022. | No outcomes of interest |
| Persiani, P., et al. (2017). "Association between spondylolisthesis and L5 fracture in patients with Osteogenesis Imperfecta." Eur Spine J **26**(12): 3106–3111. | No outcomes of interest |
| Persiani, P., et al. (2019). "Elastic intramedullary nailing of the femur fracture in patients affected by osteogenesis imperfecta type 3: Indications, limits and pitfalls." Injury **50 Suppl 2**: S52–S56. | No outcomes of interest |
| Persiani, P., et al. (2018). "Intraoperative bleeding in patients with osteogenesis imperfecta Type III treated by Fassier-Duval femoral rodding: analysis of risk factors." J Pediatr Orthop B **27**(4): 338–343. | No outcomes of interest |
| Persiani, P., et al. (2017). "Isolated olecranon fractures in children affected by osteogenesis imperfecta Type I treated with single screw or tension band wiring system: Outcomes and pitfalls in relation to bone mineral density." Medicine (Baltimore) **96**(20): e6766. | No outcomes of interest |
| Persiani, P., et al. (2019). "Treatment of tibial deformities with the Fassier-Duval telescopic nail and minimally invasive percutaneous osteotomies in patients with osteogenesis imperfecta Type III." J Pediatr Orthop B **28**(2): 179–185. | No outcomes of interest |
| Piantoni, L., et al. (2017). "Surgical treatment with pedicle screws of scoliosis associated with osteogenesis imperfecta in children." Spine Deform **5**(5): 360–365. | No outcomes of interest |
| Pichard, C. P., et al. (2009). "Surgical blood loss during femoral rodding in children with osteogenesis imperfecta." J Child Orthop **3**(4): 301–305. | No outcomes of interest |
| Plaiasu, V., et al. (2024). "TGF- β signalopathies between clinics and laboratory: case series with overlapping features of Marfan, Loeys-Dietz, Ehlers Danlos and osteogenesis imperfecta syndrome." Eur J Hum Genet **32**: 140–140. | No outcomes of interest |
| Plante, L., et al. (2016). "Effect of high-dose Vitamin D supplementation on bone density in youth with osteogenesis imperfecta: A randomized controlled trial." Bone **86**: 36–42. | No outcomes of interest |
| Pogorelic, Z., et al. (2017). "Flexible intramedullary nailing for treatment of proximal humeral and humeral shaft fractures in children: A retrospective series of 118 cases." Orthop Traumatol Surg Res **103**(5): 765–770. | Population not of interest |
| Popkov, D., et al. (2020). "Combined technique of titanium telescopic rods and external fixation in osteogenesis imperfecta patients: First 12 consecutive cases." J Orthop **22**: 316–325. | No outcomes of interest |
| Popkov, D., et al. (2019). "Use of sliding transphyseal flexible intramedullary nailing in pediatric osteogenesis imperfecta patients." Acta Orthop Belg **85**(1): 1–11. | No outcomes of interest |
| International Clinical Trials Registry Platform. (2019). "Effect of vitamin C supplementation on children with Osteogenesis Imperfecta (RBR-65q4hg)." Retrieved 25 July 2025, from https://trialsearch.who.int/Trial2.aspx?TrialID=RBR-65q4hg. | No outcomes of interest |
| Rabau, O., et al. (2023). "Scoliosis in osteogenesis imperfecta: results of posterior spinal fusion in 39 patients." Eur Spine J **32**(4): 1146–1152. | No outcomes of interest |
| Raimann, A., et al. (2020). "Health-related quality of life in paediatric patients with Osteogenesis imperfecta." Bone Reports **13**(NA): 100705–NA. | No outcomes of interest |
| Ramos-Mejia, R., et al. (2023). "Acetabular protrusion in a cohort of patients with osteogenesis imperfecta evaluated in a pediatric hospital." J Pediatr Genet **12**(1): 48–52. | No outcomes of interest |
| Rauch, F., et al. (2007). "Long-bone changes after pamidronate discontinuation in children and adolescents with osteogenesis imperfecta." Bone **40**(4): 821–827. | No outcomes of interest |
| Rauch, F., Francis H. (2004). "Osteogenesis imperfecta." Lancet (London, England) **363**(9418): 1377–1385. | No outcomes of interest |
| Rauch, F. and F. H. Glorieux (2006). "Treatment of children with osteogenesis imperfecta." Curr Osteoporos Rep **4**(4): 159–164. | No outcomes of interest |
| Rauch, F., et al. (2003). "Bone mass, size, and density in children and adolescents with osteogenesis imperfecta: effect of intravenous pamidronate therapy." J Bone Miner Res **18**(4): 610–614. | No outcomes of interest |
| Rauch, F., et al. (2010). "Genotype-phenotype correlations in nonlethal osteogenesis imperfecta caused by mutations in the helical domain of collagen type I." Eur J Hum Genet **18**(6): 642–647. | No outcomes of interest |
| Rauch, F., et al. (2010). "Relationship between genotype and skeletal phenotype in children and adolescents with osteogenesis imperfecta." J Bone Miner Res **25**(6): 1367–1374. | Irrelevant publication type |
| Rauch, F., et al. (2002). "The effects of intravenous pamidronate on the bone tissue of children and adolescents with osteogenesis imperfecta." J Clin Invest **110**(9): 1293–1299. | No outcomes of interest |
| Rauch, F., et al. (2006). "Pamidronate in children with osteogenesis imperfecta: histomorphometric effects of long-term therapy." J Clin Endocrinol Metab **91**(2): 511–516. | No outcomes of interest |
| Riaz, M., et al. (2022). "Safety and Efficacy of Zoledronic Acid in children with Osteogenesis Imperfecta." J Coll Physicians Surg Pak **32**(9): 1165–1169. | No outcomes of interest |
| Riley, E., et al. (2022). "Espr 2022." Pediatr Radiol **52**(Suppl 2): 129–179. | No outcomes of interest |
| Ross, K. E., et al. (2020). "Perioperative Considerations in Osteogenesis Imperfecta: A 20-Year Experience with the Use of Blood Pressure Cuffs, Arterial Lines, and Tourniquets." Children (Basel) **7**(11). | No outcomes of interest |
| Rothschild, L., et al. (2018). "Anesthesia in children with osteogenesis imperfecta: Retrospective chart review of 83 patients and 205 anesthetics over 7 years." Paediatr Anaesth **28**(11): 1050–1058. | No outcomes of interest |
| Rousseau, J.C., et al. (2009). "Increased cartilage type II collagen degradation in patients with osteogenesis imperfecta used as a human model of bone type I collagen alterations." Bone **46**(4): 897–900. | No outcomes of interest |
| Ruck, J., et al. (2011). "Fassier-Duval femoral rodding in children with osteogenesis imperfecta receiving bisphosphonates: functional outcomes at one year." J Child Orthop **5**(3): 217–224. | No outcomes of interest |
| Rusinska A, Michalus I, Jakubowska-Pietkiewicz E, Beska K, Adamiecka P, Chlebna-Sokol D. Difficulties in diagnostics and clinical classification of osteogenesis imperfecta in Poland. Bone Abstracts. 2017;6. | No outcomes of interest |
| Rykunov, A., et al. (2023). "P76 5-years experience of telescopic rods surgery for osteogenesis imperfecta patients in Russia." JBMR Plus **7**(S1): e10718 | No outcomes of interest |
| S. Auckburally, et al. (2022). "Does body mass index influence the risk of vertebral fractures in children with osteogenesis imperfecta?" International Conference on Children’s Bone Health 2022. | No outcomes of interest |
| Salehpour, S., et al. (2018). "57th Annual Meeting of the European Society for Paediatric Endocrinology (ESPE)." Horm Res Paediatr **90 Suppl 1**: 1–680. | No outcomes of interest |
| Salter, L., et al. (2018). "Elevated platelet counts in a cohort of children with moderate-severe osteogenesis imperfecta suggest that inflammation is present." Arch Dis Child **103**(8): 767–771. | No outcomes of interest |
| Sarikaya, I., et al. (2019). "Using a corkscrew-tipped telescopic nail in the treatment of osteogenesis imperfecta: a biomechanical study and preliminary results of 17 consecutive cases." J Pediatr Orthop B **28**(2): 173–178. | Sample size |
| Scheres, L. J. J., et al. (2018). "Adults with osteogenesis imperfecta: Clinical characteristics of 151 patients with a focus on bisphosphonate use and bone density measurements." Bone Rep **8**: 168–172. | No outcomes of interest |
| Semler O, Hoyer-Kuhn H, Stark C, Schoenau E. Results of a specialized rehabilitation approach in osteogenesis imperfecta. Bone Abstracts. 2015;4. | Sample size |
| Semler, O., et al. (2011). "Reshaping of vertebrae during treatment with neridronate or pamidronate in children with osteogenesis imperfecta." Horm Res Paediatr **76**(5): 321–327. | No outcomes of interest |
| Semler, O., et al. (2012). "A Mutation in the 5′-UTR of IFITM5 Creates an In-Frame Start Codon and Causes Autosomal-Dominant Osteogenesis Imperfecta Type V with Hyperplastic Callus." Am J Hum Genet **91**(2): 349–357. | No outcomes of interest |
| Senthilnathan, S., et al. (2008). "Two doses of pamidronate in infants with osteogenesis imperfecta." Arch Dis Child **93**(5): 398–400. | No outcomes of interest |
| Senturk L, Gulec C, Sarac Sivrikoz T, Kayserili H, Kalelioglu IH, Avci S, et al. Association of Antenatal Evaluations with Postmortem and Genetic Findings in the Series of Fetal Osteogenesis Imperfecta. Fetal Diagn Ther. 2024;51(3):285-99. | No outcomes of interest |
| Sepúlveda, A. M. T., et al. 2017). "Fracturas vertebrales en niños con osteogénesis imperfecta tipo I." Revista Chilena de Pediatria **88**(3): 348–353. | No outcomes of interest |
| Shapiro, J. R. (2014). Clinical and genetic classification of osteogenesis imperfecta and epidemiology. **NA:** 15–22. | Irrelevant publication type |
| Shapiro, J. R. (2014). SERPINH1 and Osteogenesis Imperfecta. **NA:** 159–165. | Irrelevant publication type |
| Shapiro JR, Brennen F-S. Chapter 55 - Osteogenesis Imperfecta: Maintenance of Adult Bone Health. In: Shapiro JR, Byers PH, Glorieux FH, Sponseller PD, editors. Osteogenesis Imperfecta. San Diego: Academic Press; 2014. p. 509-18. | Irrelevant publication type |
| Shapiro JR, Byers PH, Glorieux FH, Sponsellor P. Osteogenesis Imperfecta: A Translational Approach to Brittle Bone Disease: Elsevier Inc.; 2014. | Irrelevant publication type |
| Shin, C. H., et al. (2018). "Dual interlocking telescopic rod provides effective tibial stabilization in children with osteogenesis imperfecta." Clin Orthop Relat Res **476**(11): 2238–2246. | No outcomes of interest |
| Sienko, S., et al. (2023). "Surgical outcomes for spinal deformity in osteogenesis imperfecta." Spine Deform **11**(2): 391–398. | No outcomes of interest |
| Singh Kocher, M. and L. Dichtel (2011). "Osteogenesis imperfecta misdiagnosed as child abuse." J Pediatr Orthop B **20**(6): 440–443. | No outcomes of interest |
| Sithambaran, S., et al. (2022). "Bisphosphonate treatment alters the skeletal response to mechanical stimulation in children with osteogenesis imperfecta: A pilot study." JBMR Plus **6**(3): e10592. | No outcomes of interest |
| Song, Y., et al. (2019). "Health-related quality of life in children with osteogenesis imperfecta: a large-sample study." Osteoporos Int **30**(2): 461–468. | No outcomes of interest |
| Sousa, T., et al. (2014). "Musculoskeletal functional outcomes in children with osteogenesis imperfecta: associations with disease severity and pamidronate therapy." J Pediatr Orthop **34**(1): 118–122. | No outcomes of interest |
| Spahn, K. M., et al. (2019). "Fassier-Duval rods are associated with superior probability of survival compared with static implants in a cohort of children with osteogenesis imperfecta deformities." J Pediatr Orthop **39**(5): e392–e396. | No outcomes of interest |
| Steinle, J., et al. (2022). "Next-generation sequencing and analysis of consecutive patients referred for connective tissue disorders." Am J Med Genet A **188**(10): 3016–3023. | No outcomes of interest |
| Stephan, C., et al. (2022). "A comparison of anabolic and anti-resorptive treatment efficacy across varying severities of osteogenesis imperfecta." The American Society for Bone and Mineral Research. | No outcomes of interest |
| Sterian, A. G. and A. Ulici (2020). "Revision rates for osteogenesis imperfecta patients treated with telescopic nails. A follow-up study after a 7-year experience." J Med Life **13**(4): 543–547. | No outcomes of interest |
| Storoni, S., et al. (2022). "Prevalence and hospital admissions in patients with osteogenesis imperfecta in the Netherlands: A nationwide registry study." Front Endocrinol (Lausanne) **13**: 869604. | No outcomes of interest |
| Storoni, S., et al. (2023). "From genetics to clinical implications: A study of 675 Dutch osteogenesis imperfecta patients." Biomolecules **13**(2). | No outcomes of interest |
| Sugiyama, T. and S. Kawai (2001). "Changes in bone biochemical markers after high-dose cerivastatin treatment in a woman with osteogenesis imperfecta." J Bone Miner Metab **19**(6): 382–384. | Sample size |
| Sulko, J. (2004). "[Osteogenesis imperfecta–upper limb in osteogenesis imperfecta]." Chir Narzadow Ruchu Ortop Pol **69**(6): 399–402. | Not in English |
| Sullivan, B. T., et al. (2019). "Incidence of fractures from perioperative blood pressure cuff use, tourniquet use, and patient positioning in osteogenesis imperfecta." J Pediatr Orthop **39**(1): e68–e70. | No outcomes of interest |
| Suresh, K. V., et al. (2021). "Interlocking fixation in Fassier-Duval rods: Performance and success factors." J Pediatr Orthop **41**(8): 525–529. | No outcomes of interest |
| Suskauer, S. J., et al. (2003). "Temperament and physical performance in children with osteogenesis imperfecta." Pediatrics **111**(2): E153–161. | No outcomes of interest |
| Swinnen, F. K., et al. (2012). "Stapes surgery in osteogenesis imperfecta: retrospective analysis of 34 operated ears." Audiol Neurootol **17**(3): 198–206. | No outcomes of interest |
| Szczepaniak-Kubat, A., et al. (2012). "Assessment of quality of life of parents of children with osteogenesis imperfecta." Adv Clin Exp Med **21**(1): 99–104. | No outcomes of interest |
| Takeda, R., et al. (2022). "Clinical and molecular features of patients with COL1-related disorders: Implications for the wider spectrum and the risk of vascular complications." Am J Med Genet A **188**(9): 2560–2575. | No outcomes of interest |
| Tayne, S. and P. A. Smith (2019). "Olecranon fractures in pediatric patients with osteogenesis imperfecta." J Pediatr Orthop **39**(7): e558–e562. | No outcomes of interest |
| Tessaris, D., et al. (2022). "60th Annual Meeting of the European Society for Paediatric Endocrinology (ESPE): Clinical-molecular assistance pathway for primary bone fragility: a pediatric monocentric experience." Horm Res Paediatr **95**(Suppl. 2): 1–616. | No outcomes of interest |
| Thomas, I. H. D., Linda A. (2016). "Advances in the classification and treatment of osteogenesis imperfecta." Curr Osteopor Rep **14**(1): 1–9. | Irrelevant publication type |
| Topouchian, V., et al. (2004). "[Posterior spinal fusion for kypho-scoliosis associated with osteogenesis imperfecta: long-term results]." Rev Chir Orthop Reparatrice Appar Mot **90**(6): 525–532. | Not in English |
| Tosi L, Floor M, Holland R, Goerlich C, Hart T, Cuthbertson D, et al. Web-based surveys using Patient-Reported Outcome Measurement Information System (PROMIS) instruments allow documentation of important components of the disease experience among individuals with Osteogenesis Imperfecta. Bone Abstracts. 2017;6. | No outcomes of interest |
| Tosi, L. L., et al. (2015). "Initial report of the osteogenesis imperfecta adult natural history initiative." Orphanet J Rare Dis **10**: 146. | No outcomes of interest |
| Trehan, S. K., et al. (2015). "Acetabular protrusio and proximal femur fractures in patients with osteogenesis imperfecta." J Pediatr Orthop **35**(6): 645–649. | No outcomes of interest |
| Trejo, P., et al. (2017). "diaphyseal femur fractures in osteogenesis imperfecta: characteristics and relationship with bisphosphonate treatment." J Bone Miner Res **32**(5): 1034–1039. | Irrelevant publication type |
| Trejo P, Rauch F. Osteogenesis imperfecta in children and adolescents-new developments in diagnosis and treatment. Osteoporos Int. 2016;27(12):3427-37. | No outcomes of interest |
| Trigaux, C., et al. (2022). "Descemet membrane endothelial keratoplasty in a patient with descemet membrane detachment and Rieger-like anomaly associated with osteogenesis imperfecta and mutation in COL1A1." Cornea **41**(12): e24. | Irrelevant publication type |
| Tripkovic, B., et al. (2014). "[Characteristics of anesthesia in patients with osteogenesis imperfecta undergoing orthopedic surgical procedures]." Lijec Vjesn **136**(9-10): 291–295. | Not in English |
| Tsimicalis, A., et al. (2018). "Pain and quality of life of children and adolescents with osteogenesis imperfecta over a bisphosphonate treatment cycle." Eur J Pediatr **177**(6): 891–902. | No outcomes of interest |
| Vahabi, A., et al. (2024). "Characteristics of femoral neck fractures in osteogenesis imperfecta: Series of four-teen consecutive hips in twelve patients." Injury **55**(4): 111390. | No outcomes of interest |
| Veilleux, L.N.L., et al. (2015). "The functional muscle-bone unit in patients with osteogenesis imperfecta type I." Bone **79**(NA): 52–57. | No outcomes of interest |
| Verdonk, S. J. E., et al. (2023). "Medical care use among patients with monogenic osteoporosis due to rare variants in LRP5, PLS3, or WNT1." Calcif Tissue Int **113**(2): 186–194. | No outcomes of interest |
| Verra, W. C., et al. (2009). "Prevalence of vertebral pars defects (spondylolysis) in a population with osteogenesis imperfecta." Spine (Phila Pa 1976) **34**(13): 1399–1401. | No outcomes of interest |
| Violas, P., et al. (2002). "Acetabular protrusion in osteogenesis imperfecta." J Ped Orthop **22**(5): 622–625. | No outcomes of interest |
| Vitale, M. G., et al. (2007). "Osteogenesis imperfecta: determining the demographics and the predictors of death from an inpatient population." J Pediatr Orthop **27**(2): 228–232. | No outcomes of interest |
| Vuorimies, I., et al. (2017). "Bisphosphonate treatment and the characteristics of femoral fractures in children with osteogenesis imperfecta." J Clin Endocrinol Metab **102**(4): 1333–1339. | No outcomes of interest |
| Wadanamby S, Connolly D, Arundel P, Bishop NJ, Offiah A. Monitoring skull base abnormalities in children with osteogenesis imperfecta. Bone Abstracts. 2019;7. | No outcomes of interest |
| Wadanamby, S., et al. (2022). "Monitoring skull base abnormalities in children with osteogenesis imperfecta – review of current practice and a suggested clinical pathway." Bone **154**: 116235. | No outcomes of interest |
| Ward, L. M., et al. (2005). "Single-dose pharmacokinetics and tolerability of alendronate 35- and 70-milligram tablets in children and adolescents with osteogenesis imperfecta type I." J Clin Endocrinol Metab **90**(7): 4051–4056. | Sample size |
| Ward, L. M. and F. Rauch (2013). "Oral bisphosphonates for paediatric osteogenesis imperfecta?" Lancet **382**(9902): 1388–1389. | Irrelevant publication type |
| Ward, L. M. R., et al. (2002). "Osteogenesis imperfecta type VII: An autosomal recessive form of brittle bone disease." Bone **31**(1): 12–18. | No outcomes of interest |
| Wekre, L. L., et al. (2011). "Bone mass, bone markers and prevalence of fractures in adults with osteogenesis imperfecta." Arch Osteoporos **6**(1): 31–38. | No outcomes of interest |
| Wekre, L. L., et al. (2014). "Spinal deformities and lung function in adults with osteogenesis imperfecta." Clin Respir J **8**(4): 437–443. | No outcomes of interest |
| Wu, Q., et al. (2015). "Diagnosis of fetal osteogenesis imperfecta by multidisciplinary assessment: a retrospective study of 10 cases." Fetal Pediatr Pathol **34**(1): 57–64. | No outcomes of interest |
| Xi, L., et al. (2021). "Clinical and genetic analysis in 185 Chinese probands of osteogenesis imperfecta." J Bone Miner Metab **39**(3): 416–422. | No outcomes of interest |
| Yang E, C. O., et al. (2024). "Manifestations and comorbid conditions among patients with osteogenesis imperfecta (OI): A US retrospective claims database analysis." Value Health **27**(6): S1. | No outcomes of interest |
| Yang E, McCue J, Guo Y. Burden of Fractures for Individuals Living with Osteogenesis Imperfecta: Integrating Real-world Claims Data in the United States and Caregiver Panel Interviews. J Bone Miner Res. 2023;38(Suppl 2). | No outcomes of interest |
| Yang, H., et al. (2023). "Which is the best femoral implant in children with osteogenesis imperfecta? a retrospective cohort study of 783 procedures." BMC Musculoskelet Disord **24**(1): 110. | No outcomes of interest |
| Yilmaz, G., et al. (2014). "Surgical treatment of scoliosis in osteogenesis imperfecta with cement-augmented pedicle screw instrumentation." J Spinal Disord Tech **27**(3): 174–180. | No outcomes of interest |
| Yonko, E., et al. (2020). "Respiratory impairment impacts QOL in osteogenesis imperfecta independent of skeletal abnormalities." Arch Osteoporos **15**(1): 153–NA. | No outcomes of interest |
| Zack, P., et al. (2005). "Fracture and non‐fracture pain in children with osteogenesis imperfecta." Acta Paediatrica (Oslo, Norway: 1992) **94**(9): 1238–1242. | No outcomes of interest |
| Zambrano, M. B., et al. (2019). "Calcium intake improvement after nutritional intervention in paediatric patients with osteogenesis imperfecta." J Hum Nutr Diet **32**(5): 619–624. | No outcomes of interest |
| Zeitlin, L., et al. (2003). "Height and weight development during four years of therapy with cyclical intravenous pamidronate in children and adolescents with osteogenesis imperfecta types I, III, and IV." Pediatrics **111**(5 Pt 1): 1030–1036. | No outcomes of interest |
| Zhu, W., et al. (2023). "The patient-related factors in revision procedures on tibia of patients with osteogenesis imperfecta treated with the Peter-Williams nail." J Orthop Surg Res **18**(1): 532. | No outcomes of interest |
| Zhytnik L, Maasalu K, Duy BH, Prans E, Reimann E, Koks S, et al. Rib cage anomalies in a cohort of osteogenesis imperfecta patients. Bone Abstracts. 2019;7. | No outcomes of interest |
| Zurita, A. L. P., et al. (2023). "The European Registries for Rare Bone and Mineral Conditions (EuRR-Bone): Results of a survey on osteogenesis imperfecta and fibrous dysplasia mccune albright syndrome." J Bone Min Res **38**: 170–170. | No outcomes of interest |
| ZX Dong, P. C., et al. (2022). "Clinical and functional characterization of osteogenesis imperfecta Type XV: Single-centre Chinese cohort." OI Congress 2022. | No outcomes of interest |

Supplementary Table 11. Study and patient details of included studies (n=87)

| No. | Author (Year) | Country | Study design (N participants) | Study objectives | OI type |
| --- | --- | --- | --- | --- | --- |
| 1 | Abulsaad (2009)^1^ | Egypt | Prospective cohort (N=14) | Study effectiveness of intramedullary rodding | I, III, IV |
| 2 | Akcay (2008)^2^ | Turkey | Retrospective cohort (N=12) | Study effectiveness of alendronate | I, III, IV |
| 3 | Alcausin (2013)^3^ | Australia | Retrospective cohort (N=17) | Study effectiveness of IV pamidronate | I, III, V |
| 4 | Alharbi (2009)^4^ | France | Prospective cohort (N=27) | Study effectiveness and long-term safety of pamidronate | I, III, IV, V |
| 5 | Andiran (2008)^5^ | Turkey | Prospective cohort (N=14) | Study effectiveness of cyclic IV pamidronate | NR |
| 6 | Arponen (2015)^6^ | Finland | Retrospective cohort (N=39) | Study effectiveness of bisphosphonates in preventing the development of cranial base pathology | I, III, IV, VI, VII |
| 7 | Aslan (2016)^7^ | Turkey | Retrospective cohort (N=28) | Evaluate patients diagnosed with OI to make recommendations for future treatment | I, III, IV |
| 8 | Aubry-Rozier (2020)^8^ | Switzerland | Prospective cohort (N=50) | Describe the impact of the implementation of a multidisciplinary care approach for OI | I, III, IV, V |
| 9 | Bajpai (2007)^9^ | India | Prospective cohort (N=20) | Study the effectiveness of IV pamidronate | III, IV |
| 10 | Barros (2012)^10^ | Brazil | RCT (N=23) | Compare the efficacy of zoledronic acid and pamidronate | I, III, IV |
| 11 | Ben Amor (2013)^11^ | Canada | Retrospective cohort (N=25) | Describe the characteristics of patients with *COL1A1* haploinsufficiency variants | I |
| 12 | Biggin (2015)^12^ | Australia | Retrospective cohort (N=17) | Assess the effects of transition from active treatment to low dose maintenance | I, III, IV |
| 13 | Bin-Abbas (2004)^13^ | Saudi Arabia | Retrospective cohort (N=10) | Assess the effect of bisphosphonate treatment on radiological features | NR |
| 14 | Bishop (2013)^14^ | 13 countries across Europe, North and South America, Africa and Australia | RCT (N=147) | Study the safety and efficacy of risedronate | NR |
| 15 | Bradbury (2012)^15^ | UK | Prospective cohort (N=32) | Study effectiveness of risedronate | I |
| 16 | Caudevilla Lafuente (2020)^16^ | Spain | Retrospective cohort (N=40) | Describe the clinical and genetic features and treatments of patients with OI | NR |
| 17 | Chen (2022)^17^ | China | Prospective cohort (N=90) | Describe the phenotypes and genotypes of patients with OI | I, III, IV, V |
| 18 | Choi (2022)^18^ | Korea | Retrospective cohort (N=24) | Study the effectiveness of pamidronate and investigate genotype-phenotype correlation in OI | I, III, IV |
| 19 | De Wouters (2022)^19^ | Belgium | Prospective cohort (N=22) | Identify biomechanical gait deviations in children with OI | I, III, IV, IV |
| 20 | el-Sobky (2006)^20^ | Egypt | Retrospective and prospective cohorts (N=40) | Assess the effectiveness of pamidronate | I, III, IV, V, VII |
| 21 | Erbas (2022)^21^ | Turkey | Retrospective cohort (N=29) | Study patient characteristics and assess bisphosphonate treatment effectiveness | I, III, IV |
| 22 | Eren (2011)^22^ | Turkey | Retrospective cohort (N=21) | Study the clinical characteristics and genetic characteristics, as well as treatment outcomes of children and adolescents with OI | I, III, IV |
| 23 | Folkestad (2017)^23^ | Denmark | Retrospective cohort (N=416) | Compare fracture rates across the lifespan of patients with OI with a reference population | NR |
| 24 | Forin (2005)^24^ | France | Prospective cohort (N=29) | Study effectiveness of pamidronate | I, III, IV |
| 25 | Franzone (2017)^25^ | USA | Retrospective cohort (N=24) | Study effectiveness of intramedullary rodding | I, III |
| 26 | Goudriaan (2020)^26^ | Netherlands | Retrospective cohort (N=216) | Study incidence of femur fractures and non-unions | I, III, IV, V |
| 27 | Grissom (2005)^27^ | USA | Retrospective cohort (N=38) | Study effectiveness of pamidronate | NR |
| 28 | Harrington (2021)^28^ | Canada | Retrospective cohort (N=87) | Assess the utility of next-generation sequencing for the investigation of children with a clinically significant fracture history | All |
| 29 | Herdea (2021)^29^ | Romania | Retrospective cohort (N=15) | Study outcomes of intramedullary rodding | I, III |
| 30 | Huang (2006)^30^ | USA | Retrospective cohort (N=20) | Study relationship between bone mineral density, surgery, fractures and functional outcomes | I, III, IV |
| 31 | Johnson (2008)^31^ | Oman | Retrospective cohort (N=22) | Estimate the prevalence and describe clinical characteristics of OI | NR |
| 32 | Joshi (2023)^32^ | Australia | Retrospective cohort (N=44) | Characterise fracture and fracture prevalence in people with OI Type I | I |
| 33 | Kizilcan Cetin (2023)^33^ | Turkey | Retrospective cohort (N=21) | Describe clinical characteristics associated with OI and assess effectiveness and safety of bisphosphonates | I, III, IV, VI |
| 34 | Kumar (2023)^34^ | India | Prospective cohort (N=10) | Study the effectiveness of zoledronic acid in children | III, IV |
| 35 | Kumar (2016)^35^ | India | Retrospective cohort (N=26) | Study the effectiveness and safety of zoledronate | I, II, III, IV, VII, VIII |
| 36 | Kusumi (2015)^36^ | USA | Retrospective cohort (N=18) | Study the effectiveness and safety of IV pamidronate in children | I, III, IV |
| 37 | Land (2007)^37^ | Canada | Retrospective cohort (N=10) | Study the effectiveness of cyclical IV pamidronate in patients with OI Type VI | VI |
| 38 | Lee (2021)^38^ | Taiwan | Retrospective cohort (N=72) | Investigate incidence of femoral fractures and non-union | I, III, IV |
| 39 | Lhaneche (2016)^39^ | France | Retrospective cohort (N=131) | Characterize genotype and phenotype associated with changes in *SOST* gene expression in people with OI | NR |
| 40 | Li (2019)^40^ | China | Retrospective cohort (N=161) | Investigate *COL1A1* variant spectrum | I, III, IV |
| 41 | Li (2019)^41^ | China | Prospective cohort (N=42) | Study the impact of zoledronate on vertebral shape | I, III, IV |
| 42 | Li (2011)^42^ | China | Prospective cohort (N=30) | Study the effectiveness of ibandronate | I, III, IV, V |
| 43 | Lin (2008)^43^ | Taiwan | Retrospective cohort (N=26) | Study the effectiveness of IV pamidronate | I, III, IV |
| 44 | Lin (2009)^44^ | Taiwan | Retrospective cohort (N=48) | Describe clinical characteristics of people with OI | I, III, IV |
| 45 | Lin (2024)^45^ | China | Prospective cohort (N=51) | Compare effectiveness of denosumab and zoledronic acid | I, III, IV |
| 46 | Lin (2024)^46^ | China | Retrospective cohort (N=414) | Describe genotypic-phenotype correlation in OI | I, III, IV, V |
| 47 | Lindahl (2015)^47^ | Sweden | Retrospective cohort (N=223) | Describe genotypic-phenotype correlation in OI | I, III, IV |
| 48 | Lv (2016)^48^ | China | Prospective cohort (N=91) | Assess effectiveness of alendronate in paediatric patients | I, III, IV |
| 49 | Marini (2003)^49^ | USA | Prospective cohort (N=26) | Assess effectiveness of growth hormone treatment in OI | III, IV |
| 50 | Mei (2022)^50^ | China | Retrospective cohort (N=135) | Describe genotype and phenotype of people with OI | I, III, IV |
| 51 | Mulpuri (2000)^51^ | India | Retrospective cohort (N=16) | Describe outcomes of intramedullary rodding | NR |
| 52 | Ozturk (2022)^52^ | Turkey | Retrospective cohort (N=83) | Evaluate growth and pubertal characteristics of children with OI | I, III, IV, V |
| 53 | Poyrazoglu (2008)^53^ | Turkey | Prospective cohort (N=35) | Assess effectiveness of pamidronate in children | I, III, IV |
| 54 | Puvanesarajah (2015)^54^ | USA | Retrospective cohort (N=12) | Assess outcomes of sandwich technique for treating long bone non-unions | I, III, IV |
| 55 | Rodriguez Celin (2023)^55^ | USA/Canada | Prospective cohort (N=861) | Describe pain characteristics and treatments used in OI | I, III, IV |
| 56 | Rodriguez Celin (2020)^56^ | USA/Canada | Prospective cohort (N=558) | Assess the effectiveness of intramedullary rodding | III, IV |
| 57 | Saraff (2018)^57^ | UK | Retrospective cohort (N=40) | Compare the effectiveness of zoledronate and pamidronate | I, IV |
| 58 | Seikaly (2005)^58^ | USA | Prospective cohort (N=20) | Assess the effectiveness of alendronate | I, III, IV |
| 59 | Sepulveda (2017)^59^ | Chile | Retrospective cohort (N=67) | Describe the clinical characteristics of people with Type I OI | I |
| 60 | Shah (2007)^60^ | India | Retrospective cohort (N=11) | Assess effectiveness of oral bisphosphonate | I, III |
| 61 | Sun (2022)^61^ | China | Retrospective cohort (N=201) | Assess the correlation between causative variants and responses to zoledronic acid | NR |
| 62 | Tan (2023)^62^ | China | Retrospective cohort (N=28) | Characterize skeletal phenotypes of Type V OI | V |
| 63 | Thorby-Lister (2018)^63^ | UK | Retrospective cohort (N=106) | Estimate risks from radiation exposure in imaging processes; examine long bone characteristics in people with OI | NR |
| 64 | Trejo (2017)^64^ | Canada | Retrospective cohort (N=13) | Characterize the phenotype of OI Type VI | VI |
| 65 | Tuysuz (2022)^65^ | Turkey | Retrospective cohort (N=150) | Describe the variant spectrum and investigate the genotype-phenotype correlation | I, II, III, IV |
| 66 | Vallo (2006)^66^ | Spain/Mexico | Retrospective cohort (N=10) | Assess the effectiveness of long-term IV pamidronate | I, III, IV |
| 67 | Viapiana (2017)^67^ | Italy | Prospective cohort (N=114) | Assess the long-term effectiveness and safety of neridronate | I, III, IV |
| 68 | Vuorimies (2011)^68^ | Finland | Retrospective cohort (N=17) | Assess the effectiveness of zoledronic acid in children | I |
| 69 | Wei (2022)^69^ | China | Prospective cohort (N=116) | Study the genotype-phenotype correlation in people with OI | I, III, IV |
| 70 | Xu (2016)^70^ | China | Prospective cohort (N=60) | Describe the clinical phenotypes in adult OI | I, III, IV |
| 71 | Yazan (2021)^71^ | Turkey | Retrospective cohort (N=65) | Assess long-term effectiveness of bisphosphonate treatment | I, III, IV |
| 72 | Yuksel Ulker (2021)^72^ | Turkey | Prospective cohort (N=12) | Assess effectiveness of bisphosphonate in people with OI Type XI | XI |
| 73 | Zacharin (2004)^73^ | Australia/UK | Prospective cohort (N=18) | Assess effectiveness of pamidronate in children with mild OI | NR |
| 74 | Zack (2005)^74^ | UK | Prospective survey (N=35) | Describe pain experience in children with OI | I, III, IV |
| 75 | Zeitlin (2006)^75^ | Canada | Retrospective cohort (N=11) | Effects of pamidronate in children with OI Type V | V |
| 76 | Zerfu (2022)^76^ | Canada | Retrospective cohort (N=113) | Study genotype-phenotype correlation in OI | NR |
| 77 | Zheng (2022)^77^ | China | Retrospective cohort (N=21) | Investigate the clinical features and impacts of hyperplastic callus in people with Type V OI | V |
| 78 | Vyskocil (2005)^78^ | Czech Republic | Prospective cohort (N=30) | Assess effectiveness of orally administered alendronate in children | I, III, IV |
| 79 | Choi (2007)^79^ | Korea | Prospective cohort (N=11) | Assess short-term effectiveness of pamidronate treatment | I, III |
| 80 | Salehpour (2010)^80^ | Iran | Prospective cohort (N=64) | Assess effectiveness of pamidronate treatment | NR |
| 81 | Atta (2014)^81^ | Pakistan | Prospective cohort (N=72) | Assess effectiveness of IV pamidronate in children | NR |
| 82 | Lindahl (2016)^82^ | Sweden | Retrospective cohort (N=79) | Assess effectiveness of pamidronate treatment | I, III, IV |
| 83 | Oduah (2017)^83^ | South Africa | Retrospective cohort (N=78) | Review clinical presentation and management of fractures in children with OI | I, III, IV |
| 84 | Bhaskar (2019)^84^ | India | Retrospective cohort (N=21) | Assess the effectiveness of intramedullary rodding on refracture prevention | I, III, IV |
| 85 | Ohata (2019)^85^ | Japan | Retrospective cohort (N=53) | Study the genotype-phenotype relationship in OI | I, III, IV |
| 86 | Zhytnik (2019)^86^ | Ukraine | Retrospective cohort (N=143) | Assess the clinical and molecular characteristics in Ukrainian OI population | I, III, IV, V |
| 87 | Yang E (2024)^87^ | Canada | Retrospective cohort (N=565) | Estimate the prevalence of OI in Ontario | NR |

Abbreviations: IV, intravenous; NR, not reported; OI, osteogenesis imperfecta; RCT, randomised controlled trial; UK, United Kingdom; USA, United States of America.

Supplementary Table 12. Studies that met inclusion criteria but for which data extraction was not conducted (n=55)

**Reference**

Adami S, Gatti D, Colapietro F, et al. Intravenous neridronate in adults with osteogenesis imperfecta. *J Bone Min Res* 2003; 18: 126−130. 2003/01/04. DOI: 10.1359/jbmr.2003.18.1.126.

Antoniazzi F, Monti E, Venturi G, et al. GH in combination with bisphosphonate treatment in osteogenesis imperfecta. *Eur J Endocrinol* 2010; 163: 479−487. 2010/07/02. DOI: 10.1530/EJE-10-0208.

Antoniazzi F, Zamboni G, Lauriola S, et al. Early bisphosphonate treatment in infants with severe osteogenesis imperfecta. *J Pediatr* 2006; 149: 174−179. 2006/08/05. DOI: 10.1016/j.jpeds.2006.03.013.

Astrom E, Jorulf H and Soderhall S. Intravenous pamidronate treatment of infants with severe osteogenesis imperfecta. *Arch Dis Child* 2007; 92: 332−338. 2006/11/23. DOI: 10.1136/adc.2006.096552.

Bains JS, Carter EM, Citron KP, et al. A multicenter observational cohort study to evaluate the effects of bisphosphonate exposure on bone mineral density and other health outcomes in osteogenesis imperfecta. *JBMR Plus* 2019; 3: e10118. 2019/05/28. DOI: 10.1002/jbm4.10118.

Bellur S, Jain M, Cuthbertson D, et al. Cesarean delivery is not associated with decreased at-birth fracture rates in osteogenesis imperfecta. *Genet Med* 2016; 18: 570−576. 2015/10/02. DOI: 10.1038/gim.2015.131.

Bishop N, Harrison R, Ahmed F, et al. A randomized, controlled dose-ranging study of risedronate in children with moderate and severe osteogenesis imperfecta. *J Bone Min Res* 2010; 25: 32−40. 2009/07/08. DOI: 10.1359/jbmr.090712.

Brown JJ and Zacharin MR. Safety and efficacy of intravenous zoledronic acid in paediatric osteoporosis. *J Ped Endocrinol Met* 2009; 22: 55−63. 2009/04/07. DOI: 10.1515/jpem.2009.22.1.55.

Chevrel G, Schott AM, Fontanges E, et al. Effects of oral alendronate on BMD in adult patients with osteogenesis imperfecta: a 3-year randomized placebo-controlled trial. *Journal of bone and mineral research: the official journal of the American Society for Bone and Mineral Research* 2006; 21: 300−306. 2006/01/19. DOI: 10.1359/JBMR.051015.

Cubert R, Cheng EY, Mack S, et al. Osteogenesis imperfecta: mode of delivery and neonatal outcome. *Obstet Gynecol* 2001; 97: 66−69. 2001/01/12. DOI: 10.1016/s0029-7844(00)01100-5.

DiMeglio LA and Peacock M. Two-year clinical trial of oral alendronate versus intravenous pamidronate in children with osteogenesis imperfecta. *J Bone Min Res* 2006; 21: 132−140. 2005/12/16. DOI: 10.1359/JBMR.051006.

Engelbert RH, Uiterwaal CS, Gerver WJ, et al. Osteogenesis imperfecta in childhood: impairment and disability. A prospective study with 4-year follow-up. *Arch Phys Med Rehabil* 2004; 85: 772−778. 2004/05/07. DOI: 10.1016/j.apmr.2003.08.085.

Escobar C, Malveiro D, Salgado A, et al. Osteogenesis imperfecta–Experience of Dona Estefania's Hospital orthopedics' department. *Acta Medica Portuguesa* 2013; 26: 5−11.

Eylon S, Kornitzer E, Wever C, et al. Reducing fracture incidence in children with osteogenesis imperfecta: contribution of orthotics to bisphosphonates treatment. *Disabil Rehabil* 2024; 46: 1416−1421. 2023/04/18. DOI: 10.1080/09638288.2023.2198259.

Fassio A, Idolazzi L, Viapiana O, et al. THU0412 Treatment with neridronate in children and adults with osteogenesis imperfecta: data from open-label, not controlled, three-year Italian study. *Ann Rheum Dis* 2017; 76: 363. DOI: 10.1136/annrheumdis-2017-eular.2717.

Feehan AG, Zacharin MR, Lim AS, et al. A comparative study of quality of life, functional and bone outcomes in osteogenesis imperfecta with bisphosphonate therapy initiated in childhood or adulthood. *Bone* 2018; 113: 137-143. 2018/05/23. DOI: 10.1016/j.bone.2018.05.021.

Fralinger DJ, Kraft DB, Rogers KJ, et al. The fate of the bent rod in children with osteogenesis imperfecta. *J Pediatr Orthop* 2023; 43: e465-e470. 2023/04/08. DOI: 10.1097/BPO.0000000000002409.

Gatti D, Antoniazzi F, Prizzi R, et al. Intravenous neridronate in children with osteogenesis imperfecta: a randomized controlled study. *J Bone Min Res* 2005; 20: 758−763. Journal article 2005/04/13. DOI: 10.1359/JBMR.041232.

Gatti D, Rossini M, Viapiana O, et al. Teriparatide treatment in adult patients with osteogenesis imperfecta type I. *Calcif Tissue Int* 2013; 93: 448−452. 2013/08/03. DOI: 10.1007/s00223-013-9770-2.

Glorieux FH, Devogelaer JP, Durigova M, et al. BPS804 Anti-sclerostin antibody in adults with moderate osteogenesis imperfecta: Results of a randomized Phase 2a trial. *J Bone Min Res* 2017; 32: 1496−1504. 2017/04/04. DOI: 10.1002/jbmr.3143.

Hogler W, Scott J, Bishop N, et al. The effect of whole-body vibration training on bone and muscle function in children with osteogenesis imperfecta. *J Clin Endocrinol Metab* 2017; 102: 2734−2743. 2017/05/05. DOI: 10.1210/jc.2017-00275.

Hong WK, Lee DJ, Chung H, et al. Patterns of femoral neck fracture and its treatment methods in patients with osteogenesis imperfecta. *J Pediatr Orthop B* 2022; 31: e114−e121. 2021/06/03. DOI: 10.1097/BPB.0000000000000887.

Hoseinbeyki M, Moradifard S, Mirkhani F, Ehsani P, Saghiri R, Karimipoor M, et al. A preliminary data of a prospective study on Iranian patients with osteogenesis imperfecta. Bone Abstracts. 2019;7.

Hoyer-Kuhn H, Franklin J, Allo G, et al. Safety and efficacy of denosumab in children with osteogenesis imperfect--a first prospective trial. *J Musculoskelet Neuronal Interact* 2016; 16: 24−32. 2016/03/06.

Hoyer-Kuhn H, Rehberg M, Netzer C, et al. Individualized treatment with denosumab in children with osteogenesis imperfecta - follow up of a trial cohort. *Orphanet J Rare Dis* 2019; 14: 219. 2019/09/20. DOI: 10.1186/s13023-019-1197-z.

Huang S, Yang E, Krolczyk S, et al. EPH138 Osteogenesis imperfecta: epidemiology characteristics and disease burden in two real-world databases. *Value Health* 2023; 26: S189. DOI: 10.1016/j.jval.2023.03.1020.

Kiranjit Joshi RPAS. Natural history of fractures in children and adolescents with osteogenesis imperfecta type 1: A study from Western Australia. *OI Congress* 2022.

Kok DH, Sakkers RJ, Janse AJ, et al. Quality of life in children with osteogenesis imperfecta treated with oral bisphosphonates (olpadronate): a 2-year randomized placebo-controlled trial. *Eur J Pediatr* 2007; 166: 1155−1161. 2007/02/20. DOI: 10.1007/s00431-006-0399-2.

Koumakis E, Cormier-Daire V, Dellal A, et al. Osteogenesis imperfecta: characterization of fractures during pregnancy and post-partum. *Orphanet J Rare Dis* 2022; 17: 22. 2022/01/30. DOI: 10.1186/s13023-021-02148-x.

Koumakis E, Dellal A, Debernardi M, et al. Osteogenesis imperfecta: Fracture characteristics during pregnancy and post-partum. *Bone Reports* 2020; 13: 100319. DOI: 10.1016/j.bonr.2020.100319.

Letocha AD, Cintas HL, Troendle JF, et al. Controlled trial of pamidronate in children with types III and IV osteogenesis imperfecta confirms vertebral gains but not short-term functional improvement. *J Bone Min Res* 2005; 20: 977−986. 2005/05/11. DOI: 10.1359/JBMR.050109.

Li B, Yang H, Zhu W, et al. Surgical treatment of osteogenesis imperfecta: a summary of the incidence of femoral implant-related complications in children with Sillence Type I, III and IV. *Int Orthop* 2024; 48: 1645−1655. 2024/02/22. DOI: 10.1007/s00264-024-06113-8.

Li S, Cao Y, Wang H, et al. Genotypic and phenotypic analysis in chinese cohort with autosomal recessive osteogenesis imperfecta. *Front Genet* 2020; 11: 984. 2020/10/24. DOI: 10.3389/fgene.2020.00984.

Lin HY, Chuang CK, Su YN, et al. Genotype and phenotype analysis of Taiwanese patients with osteogenesis imperfecta. *Orphanet J Rare Dis* 2015; 10: 152. 2015/12/03. DOI: 10.1186/s13023-015-0370-2.

Liu J, Lin X, Sun L, et al. Safety and efficacy of denosumab in children with osteogenesis imperfecta-the first prospective comparative study. *J Clin Endocrinol Metab* 2024; 109: 1827−1836. 2024/01/10. DOI: 10.1210/clinem/dgad732.

Maasalu K, Haviko T and Martson A. Treatment of children with osteogenesis imperfecta in Estonia. *Acta Paediatr* 2003; 92: 452−455. 2003/06/13. DOI: 10.1111/j.1651-2227.2003.tb00577.x.

Munk SA, Harsevoort GJ, Gooijer K, et al. Incidence and nonunion rates of tibial fractures in adults with osteogenesis imperfecta: a retrospective cohort study of 402 patients with 42 fractures at an expert clinic. *BMC Musculoskelet Dis* 2022; 23: 1077. 2022/12/10. DOI: 10.1186/s12891-022-05966-7.

Munns CF, Rauch F, Zeitlin L, et al. Delayed osteotomy but not fracture healing in pediatric osteogenesis imperfecta patients receiving pamidronate. *J Bone Min Res* 2004; 19: 1779−1786. 2004/10/13. DOI: 10.1359/JBMR.040814.

Palomo T, Fassier F, Ouellet J, et al. Intravenous bisphosphonate therapy of young children with osteogenesis imperfecta: Skeletal findings during follow up throughout the growing years. *J Bone Min Res* 2015; 30: 2150−2157. 2015/06/11. DOI: 10.1002/jbmr.2567.

Palomo T, Andrade MC, Peters BS, et al. Evaluation of a Modified Pamidronate Protocol for the Treatment of Osteogenesis Imperfecta. *Calcif Tissue Int* 2016; 98: 42−48. 2015/09/22. DOI: 10.1007/s00223-015-0061-y.

Pavon de Paz I, Iglesias Bolanos P, Duran Martinez M, et al. [Effects of zoledronic acid in adults with osteogenesis imperfecta]. *Endocrinol Nutr* 2010; 57: 245−250. 2010/06/01. DOI: 10.1016/j.endonu.2010.03.017.

Pavon de Paz I, Rosado Sierra JA, Perez Blanco C, et al. Acute and long-term effects of zoledronate in adult patients with osteogenesis imperfecta. An observational Spanish study with five years of follow-up. *Endocrinol Diabetes Nutr (Engl Ed)* 2019; 66: 108−116. 2018/08/06. DOI: 10.1016/j.endinu.2018.05.015.

Peddada KV, Sullivan BT, Margalit A, et al. Fracture Patterns Differ Between Osteogenesis Imperfecta and Routine Pediatric Fractures. *J Pediatr Orthop* 2018; 38: e207−e212. 2018/01/23. DOI: 10.1097/BPO.0000000000001137.

Pinheiro B, Zambrano MB, Vanz AP, et al. Cyclic pamidronate treatment for osteogenesis imperfecta: Report from a Brazilian reference center. *Genet Mol Biol* 2019; 42: 252−260. 2019/05/09. DOI: 10.1590/1678-4685-GMB-2018-0097.

Plante L, Veilleux L, Weiler H, et al. Effect of high-dose vitamin D supplementation on youth with osteogenesis imperfecta: a randomized controlled trial. *Can J Diet Pract Res* 2015; 76: e2.

Rauch F, Munns CF, Land C, et al. Risedronate in the treatment of mild pediatric osteogenesis imperfecta: a randomized placebo-controlled study. *J Bone Min Res* 2009; 24: 1282−1289. 2009/03/05. DOI: 10.1359/jbmr.090213.

Rauch F, Travers R and Glorieux FH. Pamidronate in children with osteogenesis imperfecta: histomorphometric effects of long-term therapy. *J Clin Endocrinol Metab* 2006; 91: 511−516. 2005/11/18. DOI: 10.1210/jc.2005-2036.

Ruiter-Ligeti J, Czuzoj-Shulman N, Spence AR, et al. Pregnancy outcomes in women with osteogenesis imperfecta: a retrospective cohort study. *J Perinatol* 2016; 36: 828−831. 2016/07/22. DOI: 10.1038/jp.2016.111.

Sakkers R, Kok D, Engelbert R, et al. Skeletal effects and functional outcome with olpadronate in children with osteogenesis imperfecta: a 2-year randomised placebo-controlled study. Lancet 2004; 363: 1427−1431. 2004/05/04. DOI: 10.1016/S0140-6736(04)16101-1.

Shapiro JR, Thompson CB, Wu Y, et al. Bone mineral density and fracture rate in response to intravenous and oral bisphosphonates in adult osteogenesis imperfecta. *Calcif Tissue Int* 2010; 87: 120−129. 2010/06/15. DOI: 10.1007/s00223-010-9383-y.

Van Eenenaam DP, Jr., Houlihan N, Heyer JH, et al. When is an isolated olecranon fracture pathognomonic for osteogenesis imperfecta? *J Pediatr Orthop* 2022; 42: e515−e519. 2022/02/25. DOI: 10.1097/BPO.0000000000002100.

Ward LM, Rauch F, Whyte MP, et al. Alendronate for the treatment of pediatric osteogenesis imperfecta: a randomized placebo-controlled study. *J Clin Endocrinol Metab* 2011; 96: 355−364. 2010/11/26. DOI: 10.1210/jc.2010-0636.

Zacharin M and Bateman J. Pamidronate treatment of osteogenesis imperfecta--lack of correlation between clinical severity, age at onset of treatment, predicted collagen mutation and treatment response. *J Ped Endocrinol Met* 2002; 15: 163−174. 2002/03/05. DOI: 10.1515/jpem.2002.15.2.163.

Zhytnik L, Duy BH, Eekhoff M, et al. Phenotypic variation in vietnamese osteogenesis imperfecta patients sharing a recessive P3H1 pathogenic variant. *Genes (Basel)* 2022; 13 2022/03/26. DOI: 10.3390/genes13030407.

Zionts LE and Moon CN. Olecranon apophysis fractures in children with osteogenesis imperfecta revisited. *J Pediatr Orthop* 2002; 22: 745−750. 2002/11/01. DOI: 10.1097/01241398-200211000-00010.

Supplementary Table 13. Risk of bias assessment of cohort studies using the IHE Quality Appraisal Checklist (n=85)

| Study | Was the hypothesis/ aim/ objective of the study clearly stated? | Was the study conducted prospective-ly? | Were the cases collected in more than one centre? | Were patients recruited consecutive-ly? | Were the characteristics of the patients included in the study described? | Were the eligibility criteria for entry into the study clearly stated? | Were relevant outcome measures established a priority? | Changed to: Were the measures clearly described? | Was follow-up long enough for important events and outcomes to occur? | Were losses to follow-up reported? | Did the study provide estimates of random variability in the data analysis of relevant outcomes? | Were the conclusions of the study supported by results? | Were both competing interests and sources of support for the study reported? | Assessment of quality^a^ |
| --- | --- | --- | --- | --- | --- | --- | --- | --- | --- | --- | --- | --- | --- | --- |
| Abulsaad and Abdelrahman 2009 | Yes | Yes | No | No | Yes | No | No | No | Yes | No | No | Yes | No | 38% |
| Akcay, Turan et al. 2008 | Yes | No | No | Unclear | Yes | No | Yes | No | Yes | No | Yes | Yes | Yes | 54% |
| Alcausin, Briody et al. 2013 | Yes | No | No | Yes | Yes | Yes | Yes | Yes | Yes | No | Yes | Yes | No | 69% |
| Alharbi, Pinto et al. 2009 | Yes | Yes | No | Unclear | Yes | No | Yes | Yes | Yes | No | Yes | Yes | No | 62% |
| Andiran, Alikasifoglu et al. 2008 | Yes | Yes | Unclear | Unclear | Yes | No | Yes | No | Yes | Yes | Yes | Yes | No | 62% |
| Arponen, Vuorimies et al. 2015 | Yes | No | No | Unclear | Yes | Yes | No | No | Yes | Yes | Yes | Yes | No | 54% |
| Aslan, Eren et al. 2016 | Yes | No | No | Unclear | Yes | No | Yes | No | Yes | No | Yes | Yes | Yes | 54% |
| Aubry-Rozier, Richard et al. 2020 | Yes | Yes | No | No | Yes | Yes | No | No | NA | NA | No | Yes | No | 45% |
| Bajpai, Kabra et al. 2007 | Yes | Yes | No | Unclear | Yes | No | Yes | Yes | Yes | No | Yes | Yes | No | 62% |
| Ben Amor, Roughley et al. 2013 | Yes | No | No | Yes | Yes | Yes | Yes | Yes | Unclear | No | No | Yes | No | 54% |
| Biggin, Zheng et al. 2015 | Yes | No | Unclear | Unclear | Yes | Yes | Yes | Yes | Yes | No | Yes | Yes | Yes | 69% |
| Bin-Abbas, Al-Ashwal et al. 2004 | Yes | No | Yes | Unclear | Yes | No | Yes | Yes | Yes | No | Yes | Yes | No | 62% |
| Bradbury, Barlow et al. 2012 | Yes | Yes | Yes | Unclear | Yes | Yes | Yes | Yes | Yes | Yes | No | Yes | Yes | 85% |
| Caudevilla Lafuente, de Arriba Munoz et al. 2020 | Yes | No | No | Unclear | Yes | No | Yes | No | Unclear | No | Yes | Yes | Yes | 46% |
| Chen, Tan et al. 2022 | Yes | No | No | Yes | Yes | No | No | No | No | No | Yes | Yes | Yes | 46% |
| Choi, Hwang et al. 2022 | Yes | No | No | Unclear | Yes | No | Yes | No | Yes | Yes | Yes | Yes | Yes | 62% |
| De Wouters, Detrembleur et al. 2022 | Yes | Yes | No | No | Yes | No | Yes | Yes | NA | NA | No | Yes | No | 55% |
| el-Sobky, Hanna et al. 2006 | Yes | No | No | Unclear | Yes | No | No | No | NA | NA | No | Yes | No | 27% |
| Erbas, Ilgun Gurel et al. 2022 | Yes | No | Yes | Unclear | Yes | Yes | Yes | No | Yes | Yes | Yes | Yes | Yes | 77% |
| Eren, Sincar et al. 2011 | Yes | No | Unclear | Unclear | Yes | No | Yes | No | Yes | No | No | Yes | No | 38% |
| Folkestad 2017 | Yes | No | Yes | Yes | Yes | Yes | Yes | Yes | Yes | Yes | Yes | Yes | Yes | 92% |
| Forin 2005 | Yes | Yes | No | Unclear | Yes | Yes | Yes | Yes | Yes | No | Yes | Yes | No | 69% |
| Franzone, Bober et al. 2017 | Yes | No | No | Yes | Yes | Yes | Yes | Yes | Yes | Yes | Yes | Yes | No | 77% |
| Goudriaan, Harsevoort et al. 2020 | Yes | No | No | Yes | Yes | Yes | Yes | Yes | Unclear | No | No | Yes | Yes | 62% |
| Grissom, Kecskemethy et al. 2005 | Yes | No | Unclear | Unclear | Yes | No | No | Yes | Yes | Yes | No | Yes | No | 46% |
| Harrington, AlSubaihin et al. 2021 | Yes | No | No | Unclear | Yes | Yes | Yes | Yes | NA | NA | Yes | Yes | No | 64% |
| Herdea, Ulici et al. 2021 | Yes | No | No | No | Yes | Yes | Yes | No | NA | NA | No | Yes | Yes | 55% |
| Huang, Ambrose et al. 2006 | Yes | No | Unclear | Yes | Yes | Yes | Yes | Yes | Unclear | No | Yes | Yes | No | 62% |
| Johnson and Varghese 2008 | Yes | No | No | Yes | Yes | Yes | Yes | Yes | Yes | No | No | Yes | No | 62% |
| Joshi, Siafarikas et al. 2023 | Yes | No | No | Unclear | Yes | Yes | Yes | Yes | Unclear | No | Yes | Yes | No | 54% |
| Kizilcan Cetin, Siklar et al. 2023 | Yes | No | Unclear | Unclear | Yes | Yes | Yes | Yes | Yes | No | Yes | Yes | Yes | 69% |
| Kumar, Saikia et al. 2023 | Yes | Yes | No | Unclear | Yes | Yes | Yes | No | Yes | No | Yes | Yes | Yes | 69% |
| Kumar, Panigrahi et al. 2016 | Yes | No | No | Unclear | Yes | No | Yes | No | Yes | No | No | Yes | Yes | 46% |
| Kusumi, Ayoob et al. 2015 | Yes | No | No | Yes | Yes | Yes | Yes | Yes | Yes | No | Yes | Yes | No | 69% |
| Land, Rauch et al. 2007 | Yes | No | No | No | Yes | Yes | Yes | Yes | Yes | No | Yes | Yes | No | 62% |
| Lee, Liu et al. 2021 | Yes | No | No | Unclear | Yes | Yes | Yes | Yes | Unclear | No | No | Yes | No | 46% |
| Lhaneche, Hald et al. 2016 | Yes | No | Unclear | Unclear | Yes | Yes | No | Yes | Unclear | No | Yes | Yes | Yes | 54% |
| Li, Lyu et al. 2019 | Yes | No | No | Unclear | Yes | No | No | No | Yes | No | Yes | Yes | Yes | 46% |
| Li, Zheng et al. 2019 | Yes | Yes | No | Unclear | Yes | Yes | Yes | Yes | Yes | Yes | Yes | Yes | Yes | 85% |
| Li, Xia et al. 2011 | Yes | Yes | No | Unclear | Yes | Yes | Yes | Yes | Yes | No | Yes | Yes | No | 69% |
| Lin, Lin et al. 2008 | Yes | No | No | Unclear | Yes | Yes | Yes | Yes | Yes | Yes | Yes | Yes | No | 69% |
| Lin, Lin et al. 2009 | Yes | No | No | Unclear | Yes | No | Yes | Yes | Unclear | No | Yes | Yes | No | 46% |
| Lin, Hu et al. 2024 | Yes | Yes | No | Unclear | Yes | Yes | No | Yes | Yes | Yes | Yes | Yes | Yes | 77% |
| Lin, Hu et al. 2024 | Yes | No | No | Unclear | Yes | No | Yes | Yes | NA | NA | No | Yes | Yes | 55% |
| Lindahl, Astrom et al. 2015 | Yes | No | Yes | Unclear | Yes | Yes | Yes | Yes | Yes | No | Yes | Yes | Yes | 77% |
| Lv, Liu et al. 2016 | Yes | Yes | No | Unclear | Yes | No | Yes | Yes | Yes | Yes | Yes | Yes | No | 69% |
| Marini, Hopkins et al. 2003 | Yes | Yes | No | Unclear | Yes | Yes | No | No | Yes | No | Yes | Yes | No | 54% |
| Mei, Zhang et al. 2022 | Yes | No | No | Unclear | Yes | Yes | Yes | No | Unclear | No | Yes | Yes | No | 46% |
| Mulpuri and Joseph 2000 | Yes | No | No | Unclear | Yes | Yes | Yes | No | Yes | No | Yes | Yes | No | 54% |
| Ozturk, Dudakli et al. 2022 | Yes | No | No | Unclear | Yes | No | No | No | NA | NA | No | Yes | No | 27% |
| Poyrazoglu, Gunoz et al. 2008 | Yes | Yes | No | Unclear | Yes | Yes | Yes | No | Yes | No | Yes | Yes | No | 62% |
| Puvanesarajah, Shapiro et al. 2015 | Yes | No | No | Unclear | Yes | Yes | Yes | Yes | Yes | No | No | Yes | No | 54% |
| Rodriguez Celin, Kruger et al. 2023 | Yes | No | Yes | Unclear | Yes | Yes | No | No | Unclear | No | Yes | Yes | No | 46% |
| Rodriguez Celin, Kruger et al. 2020 | Yes | No | Yes | Yes | Yes | Yes | Yes | Yes | Yes | Yes | Yes | Yes | No | 85% |
| Saraff, Sahota et al. 2018 | Yes | No | No | Unclear | Yes | Yes | Yes | Yes | Yes | No | Yes | Yes | No | 62% |
| Seikaly, Kopanati et al. 2005 | Yes | Yes | No | Unclear | Yes | Yes | Yes | Yes | Yes | Yes | Yes | Yes | No | 77% |
| Sepúlveda 2017 | Yes | No | No | Unclear | Yes | Yes | Yes | Yes | NA | NA | No | Yes | No | 55% |
| Shah and Johari 2007 | Yes | No | Unclear | Unclear | Yes | No | Yes | No | No | Yes | Yes | Yes | No | 46% |
| Sun, Hu et al. 2022 | Yes | No | No | Unclear | Yes | Yes | Yes | Yes | Yes | No | Yes | Yes | Yes | 69% |
| Tan, Shek et al. 2023 | Yes | No | No | Unclear | Yes | Yes | No | No | NA | NA | No | Yes | Yes | 45% |
| Thorby-Lister, Hogler et al. 2018 | Yes | No | No | Unclear | Yes | Yes | Yes | No | Yes | No | No | Yes | No | 46% |
| Trejo, Palomo et al. 2017 | Yes | No | No | Yes | Yes | Yes | No | No | NA | NA | No | Yes | No | 45% |
| Tuysuz, Elkanova et al. 2022 | Yes | No | No | Unclear | Yes | No | No | No | Unclear | No | No | Yes | No | 23% |
| Vallo, Rodriguez-Leyva et al. 2006 | Yes | No | No | Unclear | Yes | No | Yes | No | Yes | No | No | Yes | No | 38% |
| Viapiana, Idolazzi et al. 2017. | Yes | No | No | Unclear | Yes | No | Yes | No | Yes | Yes | Yes | Yes | No | 54% |
| Vuorimies, Toiviainen-Salo et al. 2011 | Yes | No | No | Unclear | Yes | Yes | Yes | No | Yes | Yes | Yes | Yes | No | 62% |
| Wei, Yao et al. 2022 | Yes | No | No | Unclear | Yes | No | Yes | No | Yes | Yes | Yes | Yes | No | 54% |
| Xu, Ma et al. 2016 | Yes | Yes | No | Unclear | Yes | Yes | Yes | Yes | Yes | Yes | Yes | Yes | Yes | 85% |
| Yazan, Gunes et al. 2021 | Yes | No | No | Unclear | Yes | Yes | Yes | Yes | Yes | No | Yes | Yes | Yes | 69% |
| Yuksel Ulker 2021 | Yes | No | Unclear | Unclear | Yes | No | Yes | No | Yes | Yes | Yes | Yes | Yes | 62% |
| Zacharin and Kanumakala 2004 | Yes | Yes | Unclear | Unclear | Yes | No | Yes | No | Yes | Yes | Yes | Yes | No | 62% |
| Zack, Franck et al. 2005 | Yes | Yes | No | No | Yes | Yes | Yes | Yes | No | No | Yes | Yes | No | 62% |
| Zeitlin, Rauch et al. 2006 | Yes | No | No | No | Yes | Yes | Yes | Yes | Yes | No | No | Yes | No | 54% |
| Zerfu, Yong et al. 2022 | Yes | No | No | Unclear | Yes | Yes | Yes | Yes | NA | NA | Yes | Yes | No | 64% |
| Zheng, Hu et al. 2022 | Yes | No | No | Unclear | Yes | Yes | Yes | No | NA | NA | No | Yes | Yes | 55% |
| Vyskocil, Pikner et al. 2005 | Yes | Yes | No | Unclear | Yes | Yes | Yes | Yes | Yes | No | No | Yes | No | 62% |
| Choi 2007 | Yes | Yes | Unclear | Unclear | Yes | No | Yes | Yes | Yes | No | Yes | Yes | No | 62% |
| Salehpour 2010 | Yes | Yes | No | Yes | Yes | Yes | Yes | No | Yes | No | Yes | Yes | Yes | 77% |
| Atta 2014 | Yes | Yes | No | Unclear | Yes | Yes | Yes | Yes | Yes | No | Yes | Yes | No | 69% |
| Lindahl 2016 | Yes | No | Yes | Yes | Yes | Yes | Yes | Yes | Yes | No | Yes | Yes | No | 77% |
| Oduah 2017 | Yes | No | No | Unclear | Yes | No | Yes | No | Yes | No | Yes | Yes | No | 46% |
| Bhaskar 2019 | Yes | No | No | No | Yes | Yes | Yes | Yes | Yes | No | No | Yes | Yes | 62% |
| Ohata 2019 | Yes | No | Yes | Unclear | Yes | Yes | Yes | No | Yes | No | Yes | Yes | Yes | 69% |
| Zhytnik 2019 | Yes | No | No | Unclear | Yes | Yes | No | Yes | Yes | No | Yes | Yes | No | 54% |
| Yang E 2024 | Yes | No | Yes | Unclear | Yes | Yes | Yes | No | Yes | No | Yes | Yes | No | 62% |

Notes: ^a^ Green highlighting indicates high study quality with a percentage greater than 66.66%. Yellow highlighting indicates medium study quality with a percentage between 33.33% and 66.66%. Red highlighting indicates low study quality with a percentage lower than 33.33%. Most of the cohort studies were conducted retrospectively, did not provide clear eligibility criteria, and were primarily single-centre studies. Low-quality studies generally lacked a precise description of participant selection and did not clearly define outcome measures.

Supplementary Table 14. Risk of bias assessment of randomised controlled trials using the CASP RCT Checklist (n=2)

| Study | Did the study address a clearly focused research question? | Was the assignment of participants to interventions randomised? | Were all participants who entered the study accounted for at its conclusion? | Were the participants ‘blind’ to intervention they were given? | Were the investigators ‘blind’ to the intervention they were giving to participants? | Were the people assessing/ analysing outcomes ‘blinded’? | Were the study groups similar at the start of the randomised controlled trial? | Apart from the experimental intervention, did each study group receive the same level of care? | Were the effects of intervention reported comprehensive-ly? | Was the precision of the estimate of the intervention or treatment effect reported? | Assessment of quality^a^ |
| --- | --- | --- | --- | --- | --- | --- | --- | --- | --- | --- | --- |
| Barros, Saraiva et al. 2012 | Yes | Yes | Yes | Unclear | Unclear | Unclear | Yes | Yes | Yes | Yes | 70% |
| Bishop, Adami et al. 2013 | Yes | Yes | Yes | Yes | Yes | Yes | Yes | Yes | Yes | Yes | 100% |

Notes: ^a^ Green highlighting indicates high study quality with a percentage greater than 66.66%. Yellow highlighting indicates medium study quality with a percentage between 33.33% and 66.66%. Red highlighting indicates low study quality with a percentage lower than 33.33%.

Supplementary Table 15. Healing time and patient characteristics reported in 4 studies

| Study | N participants | Study type | Age at study enrolment | OI type | Fractured bones | Interventions | Healing definition | Mean healing time |
| --- | --- | --- | --- | --- | --- | --- | --- | --- |
| Abulsaad and Abdelrahman (2009)^1^ | 14 | Prospective cohort | Mean 5.92 years | I, III, IV | Femurs, tibiae | Intramedullary rodding | Clinical union | 7 weeks |
| Johnson and Varghese (2008)^31^ | 22 | Retrospective cohort | NR^a^ | NR | Upper limbs | None | NR | 1 month |
|  |  |  |  |  | Lower limbs | None |  | 6 weeks |
| Puvanesarajah, Shapiro (2015)^54^ | 12 | Retrospective cohort | Median 39 years | I, III, IV | Radii, fibulas, femurs, humeri | Compressed sandwich allograft cortical struts | Clinical union | 5.25 weeks |
| Bhaskar (2019)^84^ | 21 | Retrospective cohort | Mean 8.74 years | I, III, IV | Femurs, tibiae | Intramedullary rodding | Clinical union | 8 weeks |

Notes: ^a^While ages at study enrolment were not reported, the author classified these patients as ‘children.’ Abbreviations: NR, not reported; OI, osteogenesis imperfecta.

Supplementary Table 16. Limitations in activities of daily living due to fracture pain reported in 1 study of 34 individuals

| Activity | Individuals reporting limitations, n (%) |
| --- | --- |
| Sleeping | 29 (85) |
| Getting to sleep | 33 (97) |
| Self-care | 32 (94) |
| Travel | 26 (77) |
| School or work | 27 (79) |
| Fun activities at home | 29 (85) |
| Fun activities away from home | 29 (85) |
| Use of analgesics for pain | 22 (73) |

Data from Zack et al., 2005, of 34/79 participants who reported fractures.^74^

# References

1.	Abulsaad M, Abdelrahman A. Modified Sofield-Millar operation: less invasive surgery of lower limbs in osteogenesis imperfecta. *Int Orthop* 2009; **33**(2): 527–32.

2.	Akcay T, Turan S, Guran T, Bereket A. Alendronate treatment in children with osteogenesis imperfecta. *Indian Pediatr* 2008; **45**(2): 105–9.

3.	Alcausin MB, Briody J, Pacey V, et al. Intravenous pamidronate treatment in children with moderate-to-severe osteogenesis imperfecta started under three years of age. *Horm Res Paediatr* 2013; **79**(6): 333–40.

4.	Alharbi M, Pinto G, Finidori G, et al. Pamidronate treatment of children with moderate-to-severe osteogenesis imperfecta: a note of caution. *Horm Res* 2009; **71**(1): 38–44.

5.	Andiran N, Alikasifoglu A, Gonc N, Ozon A, Kandemir N, Yordam N. Cyclic pamidronate therapy in children with osteogenesis imperfecta: results of treatment and follow-up after discontinuation. *Journal of pediatric endocrinology & metabolism : JPEM* 2008; **21**(1): 63–72.

6.	Arponen H, Vuorimies I, Haukka J, Valta H, Waltimo-Siren J, Makitie O. Cranial base pathology in pediatric osteogenesis imperfecta patients treated with bisphosphonates. *J Neurosurg Pediatr* 2015; **15**(3): 313–20.

7.	Aslan MT, Eren E, Sağlam H, Tarım Ö. Retrospective evaluation of patients diagnosed with osteogenesis imperfecta. *Güncel Pediatri* 2017; **15**(1): 1–5.

8.	Aubry-Rozier B, Richard C, Unger S, et al. Osteogenesis imperfecta: towards an individualised interdisciplinary care strategy to improve physical activity and quality of life. *Swiss Med Wkly* 2020; **150**(28): w20285.

9.	Bajpai A, Kabra M, Gupta N, Sharda S, Ghosh M. Intravenous pamidronate therapy in osteogenesis imperfecta: response to treatment and factors influencing outcome. *J Pediatr Orthop* 2007; **27**(2): 225–7.

10.	Barros ER, Saraiva GL, de Oliveira TP, Lazaretti-Castro M. Safety and efficacy of a 1-year treatment with zoledronic acid compared with pamidronate in children with osteogenesis imperfecta. *Journal of pediatric endocrinology & metabolism : JPEM* 2012; **25**(5-6): 485–91.

11.	Ben Amor IM, Roughley P, Glorieux FH, Rauch F. Skeletal clinical characteristics of osteogenesis imperfecta caused by haploinsufficiency mutations in COL1A1. *Journal of bone and mineral research : the official journal of the American Society for Bone and Mineral Research* 2013; **28**(9): 2001–7.

12.	Biggin A, Zheng L, Briody JN, Coorey CP, Munns CF. The long-term effects of switching from active intravenous bisphosphonate treatment to low-dose maintenance therapy in children with osteogenesis imperfecta. *Horm Res Paediatr* 2015; **83**(3): 183–9.

13.	Bin-Abbas BS, Al-Ashwal AA, Al-Zayed ZS, Sakati NA. Radiological features of bisphosphonate therapy in children with osteogenesis imperfecta. *Saudi Med J* 2004; **25**(11): 1772–3.

14.	Bishop N, Adami S, Ahmed SF, et al. Risedronate in children with osteogenesis imperfecta: a randomised, double-blind, placebo-controlled trial. *Lancet* 2013; **382**(9902): 1424–32.

15.	Bradbury LA, Barlow S, Geoghegan F, et al. Risedronate in adults with osteogenesis imperfecta type I: increased bone mineral density and decreased bone turnover, but high fracture rate persists. *Osteoporos Int* 2012; **23**(1): 285–94.

16.	Caudevilla Lafuente P, Arriba Muñoz AD, Izquierdo Álvarez S, Ferrer Lozano M, Medrano San Ildefonso M, Labarta Aizpún JI. Osteogénesis imperfecta: análisis de 40 pacientes. *Med clín (Ed impr)* 2020: 512–8.

17.	Chen P, Tan Z, Qiu A, et al. Patient-reported outcomes in a Chinese cohort of osteogenesis imperfecta unveil psycho-physical stratifications associated with clinical manifestations. *Orphanet J Rare Dis* 2022; **17**(1): 249.

18.	Choi Y, Hwang S, Kim GH, Lee BH, Yoo HW, Choi JH. Genotype-phenotype correlations and long-term efficacy of pamidronate therapy in patients with osteogenesis imperfecta. *Ann Pediatr Endocrinol Metab* 2022; **27**(1): 22–9.

19.	De Wouters S, Detrembleur C, Durnez A, et al. Quantitative gait analysis in children with osteogenesis imperfecta: relationship between gait deviations and clinical features. *Acta Orthop Belg* 2022; **88**(2): 255–62.

20.	el-Sobky MA, Hanna AA, Basha NE, Tarraf YN, Said MH. Surgery versus surgery plus pamidronate in the management of osteogenesis imperfecta patients: a comparative study. *J Pediatr Orthop B* 2006; **15**(3): 222–8.

21.	Erbas IM, Ilgun Gurel D, Manav Kabayegit Z, et al. Clinical, genetic characteristics and treatment outcomes of children and adolescents with osteogenesis imperfecta: a two-center experience. *Connect Tissue Res* 2022; **63**(4): 349–58.

22.	Eren E, Sincar S, Cakir EDP, Saglam H, Tarim O. Efficacy of biphosphonates in patients with osteogenesis imperfecta/Osteogenezis imperfektali hastalarda bifosfonatlarin etkinligi. *The Journal of Current Pediatrics* 2011: 122–7.

23.	Folkestad L, Hald JD, Ersboll AK, et al. Fracture rates and fracture sites in patients with osteogenesis imperfecta: a nationwide register-based cohort study. *Journal of bone and mineral research : the official journal of the American Society for Bone and Mineral Research* 2017; **32**(1): 125–34.

24.	Forin V, Arabi A, Guigonis V, Filipe G, Bensman A, Roux C. Benefits of pamidronate in children with osteogenesis imperfecta: an open prospective study. *Joint Bone Spine* 2005; **72**(4): 313–8.

25.	Franzone JM, Bober MB, Rogers KJ, McGreal CM, Kruse RW. Re-alignment and intramedullary rodding of the humerus and forearm in children with osteogenesis imperfecta: revision rate and effect on fracture rate. *J Child Orthop* 2017; **11**(3): 185–90.

26.	Goudriaan WA, Harsevoort GJ, van Leeuwen M, Franken AA, Janus GJM. Incidence and treatment of femur fractures in adults with osteogenesis imperfecta: an analysis of an expert clinic of 216 patients. *Eur J Trauma Emerg Surg* 2020; **46**(1): 165–71.

27.	Grissom LE, Kecskemethy HH, Bachrach SJ, McKay C, Harcke HT. Bone densitometry in pediatric patients treated with pamidronate. *Pediatr Radiol* 2005; **35**(5): 511–7.

28.	Harrington J, AlSubaihin A, Dupuis L, Kannu P, Mendoza-Londono R, Howard A. Diagnostic utility of next-generation sequence genetic panel testing in children presenting with a clinically significant fracture history. *Arch Osteoporos* 2021; **16**(1): 88.

29.	Herdea A, Ulici A, Qirjako D, et al. Evaluating the functional outcomes and the quality of life for pediatric patients with osteogenesis imperfecta after fracture treatment with intramedullary rodding. *Children (Basel, Switzerland)* 2021; **8**(11).

30.	Huang RP, Ambrose CG, Sullivan E, Haynes RJ. Functional significance of bone density measurements in children with osteogenesis imperfecta. *J Bone Joint Surg Am* 2006; **88**(6): 1324–30.

31.	Johnson SC, Varghese TP. A retrospective study of osteogenesis imperfecta in Dakhliya region, Sultanate of Oman. *Oman Med J* 2008; **23**(1): 40–1.

32.	Joshi KK, Siafarikas A, Prince R. The natural history of symptomatic fractures in children and adolescents with osteogenesis imperfecta type 1: A cohort study from Western Australia. *JBMR plus* 2023; **7**(9): e10782.

33.	Kizilcan Cetin S, Siklar Z, Aycan Z, et al. Clinical characteristics and treatment outcomes of children with primary osteoporosis. *Turk Arch Pediatr* 2023; **58**(3): 314–21.

34.	Kumar A, Saikia UK, Bhuyan AK, Baro A, Prasad SG. Zoledronic acid treatment in infants and toddlers with osteogenesis imperfecta is safe and effective: A tertiary care centre experience. *Indian J Endocrinol Metab* 2023; **27**(3): 255–9.

35.	Kumar C, Panigrahi I, Somasekhara Aradhya A, Meena BL, Khandelwal N. Zoledronate for osteogenesis imperfecta: evaluation of safety profile in children. *Journal of pediatric endocrinology & metabolism : JPEM* 2016; **29**(8): 947–52.

36.	Kusumi K, Ayoob R, Bowden SA, Ingraham S, Mahan JD. Beneficial effects of intravenous pamidronate treatment in children with osteogenesis imperfecta under 24 months of age. *J Bone Miner Metab* 2015; **33**(5): 560–8.

37.	Land C, Rauch F, Travers R, Glorieux FH. Osteogenesis imperfecta type VI in childhood and adolescence: effects of cyclical intravenous pamidronate treatment. *Bone* 2007; **40**(3): 638–44.

38.	Lee CL, Liu SC, Yang CY, Chuang CK, Lin HY, Lin SP. Incidence and treatment of adult femoral fractures with osteogenesis imperfecta: An analysis of a center of 72 patients in Taiwan. *Int J Med Sci* 2021; **18**(5): 1240–6.

39.	Lhaneche L, Hald JD, Domingues A, et al. Variations of SOST mRNA expression in human bone are associated with DNA polymorphism and DNA methylation in the SOST gene. *Bone* 2016; **92**: 107–15.

40.	Li LJ, Lyu F, Song YW, et al. Genotype-phenotype relationship in a large cohort of osteogenesis imperfecta patients with COL1A1 mutations revealed by a new scoring system. *Chin Med J (Engl)* 2019; **132**(2): 145–53.

41.	Li LJ, Zheng WB, Zhao DC, et al. Effects of zoledronic acid on vertebral shape of children and adolescents with osteogenesis imperfecta. *Bone* 2019; **127**: 164–71.

42.	Li M, Xia WB, Xing XP, et al. Benefit of infusions with ibandronate treatment in children with osteogenesis imperfecta. *Chin Med J (Engl)* 2011; **124**(19): 3049–53.

43.	Lin HY, Lin SP, Chuang CK, Chen MR, Chang CY. Intravenous pamidronate therapy in Taiwanese patients with osteogenesis imperfecta. *Pediatr Neonatol* 2008; **49**(5): 161–5.

44.	Lin HY, Lin SP, Chuang CK, Chen MR, Chang CY, Niu DM. Clinical features of osteogenesis imperfecta in Taiwan. *J Formos Med Assoc* 2009; **108**(7): 570–6.

45.	Lin X, Hu J, Zhou B, et al. Efficacy and safety of denosumab vs zoledronic acid in OI adults: A prospective, open-label, randomized study. *J Clin Endocrinol Metab* 2024; **109**(7): 1873–82.

46.	Lin X, Hu J, Zhou B, et al. Genotype-phenotype relationship and comparison between eastern and western patients with osteogenesis imperfecta. *J Endocrinol Invest* 2024; **47**(1): 67–77.

47.	Lindahl K, Astrom E, Rubin CJ, et al. Genetic epidemiology, prevalence, and genotype-phenotype correlations in the Swedish population with osteogenesis imperfecta. *Eur J Hum Genet* 2015; **23**(8): 1042–50.

48.	Lv F, Liu Y, Xu X, et al. Effects of long-term alendronate Treatment on a large sample of pediatric patients with osteogenesis imperfecta. *Endocr Pract* 2016; **22**(12): 1369–76.

49.	Marini JC, Hopkins E, Glorieux FH, et al. Positive linear growth and bone responses to growth hormone treatment in children with types III and IV osteogenesis imperfecta: high predictive value of the carboxyterminal propeptide of type I procollagen. *Journal of bone and mineral research : the official journal of the American Society for Bone and Mineral Research* 2003; **18**(2): 237–43.

50.	Mei Y, Zhang H, Zhang Z. Comparing clinical and genetic characteristics of de novo and inherited COL1A1/COL1A2 variants in a large chinese cohort of osteogenesis imperfecta. *Frontiers in endocrinology* 2022; **13**: 935905.

51.	Mulpuri K, Joseph B. Intramedullary rodding in osteogenesis imperfecta. *J Pediatr Orthop* 2000; **20**(2): 267–73.

52.	Ozturk AP, Dudakli A, Ozturan EK, Poyrazoglu S, Bas F, Darendeliler F. Growth and pubertal features in a cohort of 83 patients with osteogenesis imperfecta. *Klin Padiatr* 2022; **234**(4): 199–205.

53.	Poyrazoglu S, Gunoz H, Darendeliler F, et al. Successful results of pamidronate treatment in children with osteogenesis imperfecta with emphasis on the interpretation of bone mineral density for local standards. *J Pediatr Orthop* 2008; **28**(4): 483–7.

54.	Puvanesarajah V, Shapiro JR, Sponseller PD. Sandwich allografts for long-bone nonunions in patients with osteogenesis imperfecta: a retrospective study. *J Bone Joint Surg Am* 2015; **97**(4): 318–25.

55.	Rodriguez Celin M, Kruger KM, Caudill A, et al. A multicenter study to evaluate pain characteristics in osteogenesis imperfecta. *Am J Med Genet A* 2023; **191**(1): 160–72.

56.	Rodriguez Celin M, Kruger KM, Caudill A, et al. A multicenter study of intramedullary rodding in osteogenesis imperfecta. *JB JS Open Access* 2020; **5**(3): e20.00031.

57.	Saraff V, Sahota J, Crabtree N, Sakka S, Shaw NJ, Hogler W. Efficacy and treatment costs of zoledronate versus pamidronate in paediatric osteoporosis. *Arch Dis Child* 2018; **103**(1): 92–4.

58.	Seikaly MG, Kopanati S, Salhab N, et al. Impact of alendronate on quality of life in children with osteogenesis imperfecta. *J Pediatr Orthop* 2005; **25**(6): 786–91.

59.	Sepulveda AM, Terrazas CV, Saez J, Reyes ML. [Vertebral fractures in children with Type I Osteogenesis imperfecta]. *Rev Chil Pediatr* 2017; **88**(3): 348–53.

60.	Shah I, Johari A. Oral bisphosphonate treatment for osteogenesis imperfecta—an Indian perspective. *Annals of tropical paediatrics* 2007; **27**(1): 39–43.

61.	Sun L, Hu J, Liu J, et al. Relationship of pathogenic mutations and responses to zoledronic acid in a cohort of osteogenesis imperfecta children. *J Clin Endocrinol Metab* 2022; **107**(9): 2571–9.

62.	Tan Z, Shek HT, Dong Z, et al. Retrospective analyses of clinical features in 28 Chinese patients with type V osteogenesis imperfecta: new perspectives in an old issue. *Osteoporos Int* 2023; **34**(2): 369–77.

63.	Thorby-Lister A, Hogler W, Hodgson K, et al. Cumulative radiation exposure from medical imaging and associated lifetime cancer risk in children with osteogenesis imperfecta. *Bone* 2018; **114**: 252–6.

64.	Trejo P, Palomo T, Montpetit K, et al. Long-term follow-up in osteogenesis imperfecta type VI. *Osteoporos Int* 2017; **28**(10): 2975–83.

65.	Tuysuz B, Elkanova L, Uludag Alkaya D, et al. Osteogenesis imperfecta in 140 Turkish families: Molecular spectrum and, comparison of long-term clinical outcome of those with COL1A1/A2 and biallelic variants. *Bone* 2022; **155**: 116293.

66.	Vallo A, Rodriguez-Leyva F, Rodriguez Soriano J. Osteogenesis imperfecta: anthropometric, skeletal and mineral metabolic effects of long-term intravenous pamidronate therapy. *Acta Paediatr* 2006; **95**(3): 332–9.

67.	Viapiana O, Idolazzi L, Fassio A, et al. Long-term effects of neridronate in adults with osteogenesis imperfecta: An observational three-year italian study. *Calcif Tissue Int* 2017; **100**(4): 341–7.

68.	Vuorimies I, Toiviainen-Salo S, Hero M, Makitie O. Zoledronic acid treatment in children with osteogenesis imperfecta. *Horm Res Paediatr* 2011; **75**(5): 346–53.

69.	Wei S, Yao Y, Shu M, et al. Genotype-phenotype relationship and follow-up analysis of a chinese cohort with osteogenesis imperfecta. *Endocr Pract* 2022; **28**(8): 760–6.

70.	Xu XJ, Ma DD, Lv F, et al. The clinical characteristics and efficacy of bisphosphonates in adult patients with osteogenesis impergecta. *Endocr Pract* 2016; **22**(11): 1267–76.

71.	Yazan H, Gunes N, Akpinar E, Ozyalvac ON, Uludag Akkaya D, Tuysuz B. Effects of long-term pamidronate treatment on bone density and fracture rate in 65 osteogenesis imperfecta patients. *Turk Arch Pediatr* 2021; **56**(5): 474–8.

72.	Yuksel Ulker A, Uludag Alkaya D, Elkanova L, et al. Long-term follow-up outcomes of 19 patients with osteogenesis imperfecta Type XI and Bruck syndrome Type I caused by FKBP10 variants. *Calcif Tissue Int* 2021; **109**(6): 633–44.

73.	Zacharin M, Kanumakala S. Pamidronate treatment of less severe forms of osteogenesis imperfecta in children. *Journal of pediatric endocrinology & metabolism : JPEM* 2004; **17**(11): 1511–7.

74.	Zack P, Franck L, Devile C, Clark C. Fracture and non-fracture pain in children with osteogenesis imperfecta. *Acta Paediatr* 2005; **94**(9): 1238–42.

75.	Zeitlin L, Rauch F, Travers R, Munns C, Glorieux FH. The effect of cyclical intravenous pamidronate in children and adolescents with osteogenesis imperfecta type V. *Bone* 2006; **38**(1): 13–20.

76.	Zerfu T, Yong B, Harrington J, Howard A. Does the skeletal phenotype of osteogenesis imperfecta differ for patients with non-COL1A1/2 mutations? A retrospective study in 113 patients. *J Pediatr Orthop* 2022; **42**(5): e507–e14.

77.	Zheng WB, Hu J, Zhang J, et al. Specific characteristic of hyperplastic callus in a larger cohort of osteogenesis imperfecta Type V. *Calcif Tissue Int* 2022; **110**(4): 451–63.

78.	Vyskocil V, Pikner R, Kutilek S. Effect of alendronate therapy in children with osteogenesis imperfecta. *Joint Bone Spine* 2005; **72**(5): 416–23.

79.	Choi JH, Shin YL, Yoo HW. Short-term efficacy of monthly pamidronate infusion in patients with osteogenesis imperfecta. *J Korean Med Sci* 2007; **22**(2): 209–12.

80.	Salehpour S, Tavakkoli S. Cyclic pamidronate therapy in children with osteogenesis imperfecta. *Journal of pediatric endocrinology & metabolism : JPEM* 2010; **23**(1-2): 73–80.

81.	Atta I, Iqbal F, Lone SW, Ibrahim M, Khan YN, Raza J. Effect of intravenous pamidronate treatment in children with osteogenesis imperfecta. *J Coll Physicians Surg Pak* 2014; **24**(9): 653–7.

82.	Lindahl K, Kindmark A, Rubin CJ, et al. Decreased fracture rate, pharmacogenetics and BMD response in 79 Swedish children with osteogenesis imperfecta types I, III and IV treated with Pamidronate. *Bone* 2016; **87**(NA): 11–8.

83.	Oduah GO, Firth GB, Pettifor JM, Thandrayen K. Management of osteogenesis imperfecta at the Chris Hani Baragwanath Hospital. *SA Orthopaedic Journal* 2017; **16**(2): 19–25.

84.	Bhaskar AR, Khurana D. Results of rodding and impact on ambulation and refracture in osteogenesis imperfecta: Study of 21 children. *Indian J Orthop* 2019; **53**(4): 554–9.

85.	Ohata Y, Takeyari S, Nakano Y, et al. Comprehensive genetic analyses using targeted next-generation sequencing and genotype-phenotype correlations in 53 Japanese patients with osteogenesis imperfecta. *Osteoporos Int* 2019; **30**(11): 2333–42.

86.	Zhytnik L, Maasalu K, Pashenko A, et al. COL1A1/2 pathogenic variants and phenotype characteristics in ukrainian osteogenesis imperfecta patients. *Front Genet* 2019; **10**(NA): 722.

87.	Yang E, Kainth P, Nair S, Nightingale N, Rodopoulou S. EPH65 Burden of Illness of Osteogenesis Imperfecta in Ontario, Canada. *Value in Health* 2024; **27**(6): S165.
